# Integrated Transcriptomics and Proteomics to Reveal Regulation Mechanism and Evolution of *SmWRKY61* on Tanshinone Biosynthesis in *Salvia miltiorrhiza* and *Salvia castanea*

**DOI:** 10.3389/fpls.2021.820582

**Published:** 2022-03-03

**Authors:** Yue Chen, Yanting Wang, Juan Guo, Jian Yang, Xiaodan Zhang, Zixuan Wang, Ying Cheng, Zewei Du, Zhechen Qi, Yanbo Huang, Mans Dennis, Yukun Wei, Dongfeng Yang, Luqi Huang, Zongsuo Liang

**Affiliations:** ^1^College of Life Sciences and Medicine, Key Laboratory of Plant Secondary Metabolism and Regulation in Zhejiang Province, Zhejiang Sci-Tech University, Hangzhou, China; ^2^State Key Laboratory of Dao-di Herbs, National Resource Center for Chinese Materia Medica, China Academy of Chinese Medical Sciences, Beijing, China; ^3^Eastern China Conservation Centre for Wild Endangered Plant Resources, Shanghai Chenshan Botanical Garden, Shanghai, China; ^4^Faculty of Medical Sciences, Anton de Kom University of Suriname, Paramaribo, Suriname

**Keywords:** secondary metabolism, *Salvia miltiorrhiza*, *Salvia* Linn, proteome, transcriptome, *WRKYs*

## Abstract

Tanshinones found in *Salvia* species are the main active compounds for the treatment of cardiovascular and cerebrovascular diseases, but their contents are hugely different in different species. For example, tanshinone IIA content in *Salvia castanea* Diels f. *tomentosa* Stib. is about 49 times higher than that in *Salvia miltiorrhiza* Bunge. The molecular mechanism responsible for this phenomenon remains largely unknown. To address this, we performed comparative transcriptomic and proteomic analyses of *S. miltiorrhiza* and *S. castanea*. A total of 296 genes in *S. castanea* and 125 genes in *S. miltiorrhiza* were highly expressed at both the transcriptional and proteome levels, including hormone signal regulation, fungus response genes, transcription factors, and CYP450. Among these differentially expressed genes, the expression of *SmWRKY61* was particularly high in *S. castanea*. Overexpression of *SmWRKY61* in *S. miltiorrhiza* could significantly increase the content of tanshinone I and tanshinone IIA, which were 11.09 and 33.37 times of the control, respectively. Moreover, *SmWRKY61* had a strong regulatory effect, elevating the expression levels of tanshinone pathway genes such as *DXS2, CMK, HMGS2, 1, KSL1, KSL2, CYP76AH1*, and *CYP76AK3*. For the WRKY family, 79 *SmWRKY*s were originally obtained and classified into three main groups. Collinearity analysis indicated a more specific extension of *WRKY* gene family in *Salvia* genus. In 55 *Salvia* species, only 37 species contained the *WRKY61* sequence, and high *SmWRKY61* expression in some *Salvia* L. species was often accompanied by high tanshinone accumulation. The above results suggest that *SmWRKY61* is a highly effective regulator of tanshinone accumulation and may be a key factor resulting in high tanshinone accumulation in *S. castanea*.

## Introduction

*Salvia miltiorrhiza* Bunge (*S. miltiorrhiza*), a perennial herb in *Salvia* L. (*Lamiaceae: Nepetoideae: Mentheae: Salviinae*) (Xu et al., 2016), has high medicinal and economic value (Ma et al., 2012). It is known for its pharmacologically effective chemical components (Fang et al., 2018), primarily diterpene quinone compounds (e.g., tanshinone IIA, cryptotanshinone, dihydrotanshinone I, isocryptotanshinone, and przewaquinone) and polyphenolic compounds (e.g., rosmarinic acid, caffeic acid, and salvianolic acid) (Kai et al., 2011). Tanshinones are important bioactive terpenoids distributed in *Salvia* L., and the biosynthesis of tanshinone depends on the mevalonate pathway (MVA) in the cytoplasm and the 2-C-methyl-D-erythritol 4-phosphate (MEP) pathway in the plasmid (Rohmer et al., 1993; Newman and Chappell, 1999). MVA and MEP pathways result in the formation of intermediates, isopentenyl diphosphate (IPP) and dimethylallyl diphosphate (DMAPP) (Shi et al., 2016). IPP and DMAPP are catalyzed to produce the precursor of geranylgeranyl diphosphate (GGPP) by geranylgeranyl diphosphate synthase (GGPPS) (Kai et al., 2010). GGPP can be synthesized to ferruginol by copalyl diphosphate synthase (CPS), ent-kaurene synthase-like (KSL), and P450 family protein *CYP76AH1* (Kai et al., 2010). The CPS and KSL may act as important key enzymes for the formation of various diterpenoids in *S. miltiorrhiza*, including tanshinones, gibberellins, and ent-13-epi-manoyl oxide (Gao et al., 2009). Many downstream steps of the tanshinone biosynthetic pathway may be catalyzed by cytochrome P450 (CYP450s), including *CYP76AHs*, *CYP76AKs*, and *CYP71D*s (Guo et al., 2013; Zi et al., 2014; Guo et al., 2016; Ma et al., 2021).

*Salvia castanea* Diels f. *tomentosa* Stib, distributed in Tibet at an altitude of 2,500–3,750 m, is used as a substitute for *S. miltiorrhiza* for the treatment of various cardiovascular diseases (Li et al., 2008). *S. castanea* produces high contents of rosmarinic acid (9.5 times of *S. miltiorrhiza*) and high contents of tanshinone IIA (4.7 times of *S. miltiorrhiza*), but little salvianolic acid B (0.02 times of *S. miltiorrhiza*) (Yang et al., 2009). Caffeic acid and rosmarinic acid are the main phenolic compounds in *S. castanea* (Yang et al., 2009, 2012). Its leaves are densely covered with gray downy hairs, purplish-brown corolla, and oblique incomplete pubescent rings on the inner surface (Supplementary Figures 1A,B). *S. castanea* has thick, twisted, and purplish-brown striped roots, which are generally unbranched and form four prisms (Supplementary Figures 1C,D). Crosssections of the roots show that *S. castanea* roots are twisted together, whereas *S. miltiorrhiza* has only one root with gray yellow or purplish brown xylem and yellow and white vessels (Supplementary Figures 1E,F). The high accumulation of secondary metabolites and the peculiar morphological characteristics of *S. castanea* may make it adaptable to various environment and climate changes. These differences manifest that *S. miltiorrhiza* and *S. castanea* differ in secondary metabolism and morphological features, but the mechanisms underlying this difference are still unknown.

Transcriptomics data and isobaric tags for relative and absolute quantification (iTRAQ)-based quantitative proteomics analysis brings new insight to reveal some new genes of tanshinone biosynthesis. With the rapid development of sequencing technology, a high-quality reference genome of *S. miltiorrhiza* information was obtained by combining PacBio sequencing technologies. Comparative transcriptomics techniques can be used to understand the differential expression of genes in specific tissues at specific times, to discover genes associated with specific physiological functions, and to infer the physiological functions of unknown genes (Gao et al., 2009). Proteomics has become one of the main approaches used to analyze and understand biological systems. The study of the omeics-related differentially expressed genes in *S. miltiorrhiza* and *S. castanea* may provide the basis for further study on transcriptional regulation of secondary metabolism.

The present study was about to elucidate the accumulation mechanisms of tanshinones and polyphenolic acids in *S. castanea* and *S. miltiorrhiza* by comparatively analyzing the transcriptome and proteome. We identified dozens of differentially expressed transcription factors, in which we found that *SmWRKY61* in *S. castanea* was 4.12 times higher than that in *S. miltiorrhiza* at the transcriptional level. Quantitative real-time PCR (qRT-PCR) was performed to determine the expression of *WRKYs*. *SmWRKY61* was expressed 5,564 times higher than *S. miltiorrhiza*, indicating that *SmWRKY61* was an important regulatory factor for high tanshinone accumulation in *S. castanea*. Overexpression of *SmWRKY61* in *S. miltiorrhiza* could significantly increase the contents of tanshinone I and tanshinone IIA. Subsequently, analyses of chromosome distribution, gene duplications, phylogeny, synteny analysis, global expression, phylogenetic and motif compositions analyses were further performed to identify the molecular evolution of specific *WRKY61* in 55 species of *Salvia* L.

## Results

### Accumulation of Tanshinones and Polyphenolic Acids in *Salvia castanea* and *Salvia miltiorrhiza* Hairy Roots

**FIGURE 1:**
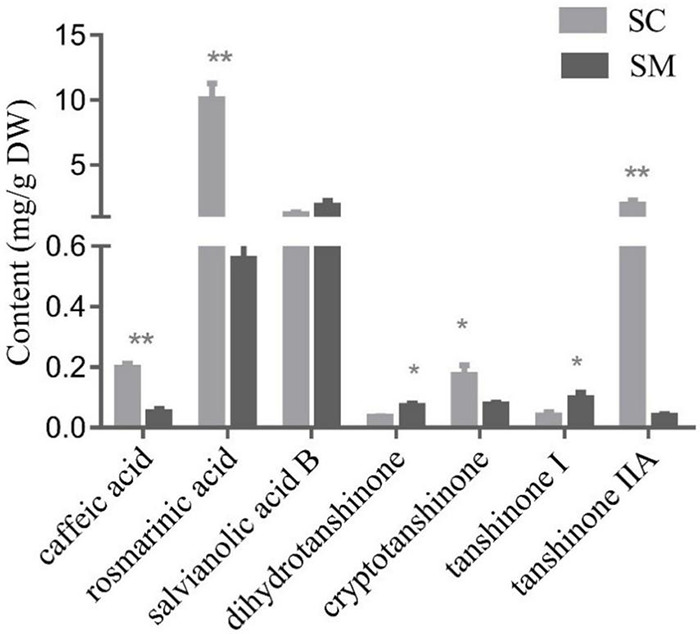
Contents of tanshinones and polyphenolic compounds in *Salvia miltiorrhiza* and *Salvia castanea* hairy roots. HPLC analysis of phenolic acids and tanshinones in *S. miltiorrhiza* and *S. castanea* hairy roots. Error bars represent the standard deviation (SD, *n* = 3 biologically independent samples; “*” represents 0.01 < *p* < 0.05, “**” represents *p* < 0.01).

In the present study, phenolic acid compounds (caffeic acid, rosmarinic acid, and salvianolic acid B) and lipid-soluble tanshinone compounds (dihydrotanshinone, cryptotanshinone, tanshinone I, and tanshinone IIA) were examined in the hairy roots of *S. miltiorrhiza* and *S. castanea*. In general, the contents of tanshinone and phenolic acid in *S. castanea* were higher than those in *S. miltiorrhiza.* Specifically, the rosmarinic acid and caffeic acid levels in *S. castanea* were found to be 17.98 and 3.83 times higher than those in *S. miltiorrhiza*, respectively (Figure 1). Similarly, the tanshinone IIA and cryptotanshinone contents were 49.28 and 2.28 times higher than *S. castanea*, respectively (Figure 1). In contrast, the levels of dihydrotanshinone (3.32 times higher) and tanshinone I (3.07 times higher) were higher in *S. miltiorrhiza* than *S. castanea* (Figure 1). Collectively, these results demonstrated that although the active compounds in the two species were similar, but their relative abundances were different, which was consistent with our previous research (Fang et al., 2018).

### Comparative Transcriptomics Analysis of *Salvia miltiorrhiza* and *Salvia castanea* to Identify Differentially Expressed Biosynthetic Genes and Transcription Factors

To obtain an overview of the *S. miltiorrhiza* and *S. castanea* transcriptomes, total RNA samples were obtained after 24 days of hairy root growth. A total of 29,644,212 sequence reads from the *S. miltiorrhiza* transcriptome and 35,421,130 sequence reads from the *S. castanea* transcriptome were generated. Among them, 6,897 sequences were in *S. castanea* and 5,506 were upregulated in *S. miltiorrhiza* (Supplementary Table 1). At the transcriptional level, 12,403 significantly differentially expressed genes were annotated into 32 metabolic pathways (Supplementary Figure 8).

Hormone-related signaling molecules are the key plant-specific signaling molecules, which can respond to various environmental stress and are involved in the synthesis of secondary metabolites, such as jamonates signaling, abscisic acid pathway, and gibberellin signaling (Supplementary Figure 8). Further analysis revealed that 12 genes were significantly differentially expressed and related to jasmonic acid signaling (Supplementary Table 2). Lipoxygenase (*LOX*), a key gene involved in jasmonates signaling pathway, acting as a potent signaling molecule in plants, was related to important secondary metabolites. The sequence similarity between *comp22754_c0_seq1* and lipoxygenase was up to 73%, indicating that *comp22754_c0_seq1* may regulate the synthesis of tanshinone or salvianolic acid by affecting the jasmonic acid signaling pathway. Likewise, 116 sequences belonged to abscisic acid response genes (Supplementary Table 3), of which 39 sequences were significantly differentially expressed. *ApNAC1* can regulate biosynthesis of androstenone in *Andrographis paniculate* (Jian et al., 2017). Our study found that the *comp8895_c0* sequence was similar to the *ApNAC1* sequence, indicating that *comp8895_c0* may be related to the synthesis of tanshinone (Jian et al., 2017). Twenty-seven sequences belonged to gibberellin response genes (Supplementary Table 4), of which 11 sequences were significantly differential expressed.

Research has revealed that transcription factors can regulate multiple genes involved in all kinds of biosynthetic pathways. Our research found that 80 MYB transcription factors were differentially expressed in *S. miltiorrhiza* and *S. castanea* (Supplementary Table 6). MYB transcription factors are possibly involved in regulating the phenolic acids pathway in *S. miltiorrhiza*. A recent study showed that *SmMYB1* activated some genes involved in the anthocyanin biosynthesis pathway (Zhou et al., 2021), which was significantly upregulated in *S. castanea.* Therefore, we speculated that *SmMYB1* can regulate the contents of phenolic acids. The bHLH (basic helix–loop–helix) family is the most widely present transcription factors family, which may play a key role in regulating the biosynthesis of secondary metabolites (Zhang et al., 2020). Correspondingly, 68 bHLH transcription factors were differentially expressed in *S. miltiorrhiza* and *S. castanea* (Supplementary Table 7). Overexpression of *SmbHLH3* decreased both the polyphenolic compounds and tanshinone contents (Zhang et al., 2020), which were significantly upregulated in *S. castanea*. The overexpression of *SmbHLH148* significantly increased phenolic acid and tanshinone components (Xing et al., 2018a), which were downregulated in *S. castanea*. The WRKY family is a large transcription factors family that can regulate secondary metabolite biosynthesis, and 25 WRKY transcription factors (Supplementary Table 8) were differentially expressed in *S. castanea*. Typically, overexpression of *SmWRKY9* can significantly stimulate rosmarinic acid accumulation, which may directly and positively regulate the expression of *SmRAS1* and *SmCYP98A14* to promote the biosynthesis of rosmarinic acid (Zhang, 2019). *SmWRKY61* showed a significantly differential expression, and its RPKM in *S. castanea* was 32.23, which was 4.12 times higher in *S. miltiorrhiza.* It is reasonable to assume that *SmWRKY9* and *SmWRKY61* could activate key enzyme genes in downstream pathway, regulating the accumulation of tanshinone and salvianolic acid. These transcription factors may be one of the reasons for the high accumulation of tanshinone IIA and low accumulation of salvianolic acid.

Post-modification enzymes cytochrome P450s (CYP450s) were involved in plant secondary metabolic pathways. With the discovery of the terpenoid biosynthesis pathway, CYP450s widely took part in the downstream reaction of tanshinone biosynthetic steps. We found 139 cytochrome P450 (CYP450) genes (Supplementary Table 9) differentially expressed between *S. miltiorrhiza* and *S. castanea*. CYP71D subfamily members were functionally involved in indole alkaloids and flavonoids biosynthesis, as well as in terpenoid biosynthesis. In this work, many differentially expressed *CYP71Ds* were found, including *CYP71D10, CYP71D11, CYP71D12, CYP71D15, CYP71D55, CYP71D9*, and *CYP71D95*, which were significantly different (Supplementary Table 9). The above results indicated that these genes might be candidate genes involved in tanshinone and salvianolic acid biosynthesis.

**FIGURE 2:**
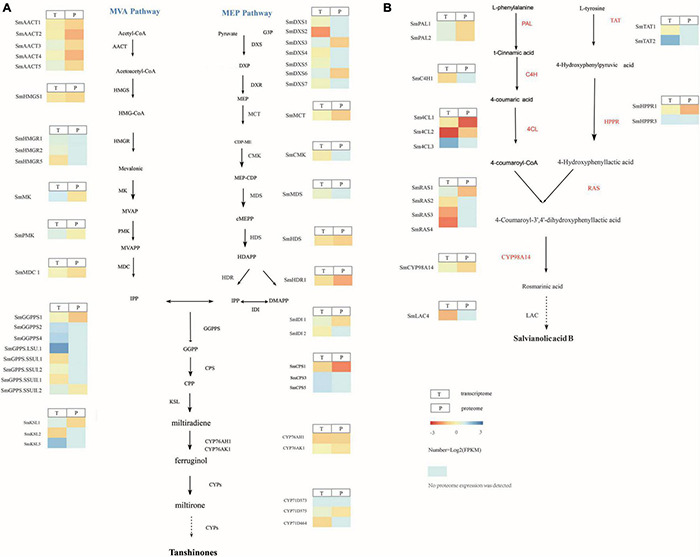
Gene and protein expression in *S. miltiorrhiza* and *S. castanea*. **(A)** Gene expression heatmap for the tanshinone pathways. **(B)** Gene expression heatmap for the salvianolic acid pathways, displaying relative expression levels at the transcriptome (T) and proteome (P) levels. The expression level means Z-score of log2FC (*S. castanea*/*S. miltiorrhiza*) of transcriptome and quant ratio of the proteome (*S. castanea*/*S. miltiorrhiza*). Source data underlying this Figure are provided in Supplementary Tables 16, 17.

Transcriptome analysis showed that the tanshinone synthesis-related genes *SmDXS2*, *SmHDR1*, *SmGPPS.SSUII.1*, *SmCPS1*, and *SmKSL2* were upregulated in *S. castanea* at 9.53, 2.56, 4.27, 3.60, and 4.34 times, higher than that in *S. miltiorrhiza*, respectively (Figure 2A). *CYP71D375*, *CYP76AK1, SmMK*, and *SmPMK* were downregulated in *S. castanea* at 0.697, 0.705, 0.739, and 0.548 times in *S. miltiorrhiza*, respectively, compared with *S. miltiorrhiza.* As shown in Figure 2B, the phenolic acid synthesis-related gene *PAL1* was upregulated in *S. miltiorrhiza*, which was 15.1 times that in *S. castanea*. The expression of *4CL1* in *S. castanea* was 4.05 times higher than that in *S. miltiorrhiza*. Several laccase genes (*LAC4*) encode proteins that may catalyze the oxidative reaction from rosmarinic acid to salvianolic acid B, and their expression was 2.02 times differ from *S. miltiorrhiza.* The difference in gene expression has revealed the mechanism of high accumulation of tanshinone and rosmarinic acid and low accumulation of salvianolic acid B in *S. castanea*.

### Proteome Properties Revealed the Differential Expression Mechanisms of *Salvia miltiorrhiza* and *Salvia castanea*

Isobaric tags for relative and absolute quantification analysis was carried on *S. castanea* and *S. miltiorrhiza*. A total of 812 differentially expressed proteins were identified, 348 of which were upregulated in *S. castanea*, and 469 of which were upregulated in *S. miltiorrhiza* (Supplementary Table 10). These differentially expressed proteins were enriched in phenylpropanoid biosynthesis, glutathione metabolism, and steroid synthesis (Supplementary Figure 8). Further analysis showed that 5 sequences were related to jasmonic acid signaling (Supplementary Table 11), 14 sequences were related to abscisic acid response genes (Supplementary Table 12), and 9 sequences were related to fungal response genes (Supplementary Table 13). CYP450s and 2OGDs are involved in the downstream step of tanshinone biosynthesis, following the hydroxylation of CYP450s (Xu and Song, 2017). Functional analysis revealed that 2OGD5 contributes to miltirone biosynthesis, regulating the oxidation function after the hydroxylation of CYP450s in miltirone, cryptotanshinone, and tanshinone IIA biosynthesis (Kawai et al., 2014). After combined transcriptome and proteome analysis, 58 2OGDs were identified in *S. castanea* and *S. miltiorrhiza* (Supplementary Table 15), of which five 2OGDs were significantly differentially expressed, indicating that 2OGDs participated in flavonol synthase and regulated the accumulation of tanshinone and salvianolic acid.

Proteomic analysis showed that 45 CYP450 proteins (Supplementary Table 14) and 12 proteins were significantly differentially expressed in *S. castanea* and *S. miltiorrhiza*. These genes included *SmPMK*, *SmMK, SmHDR1, SmCPS1*, *CYP71D375*, and *CYP76AK1*, which were 0.548, 0.739, 1.259, 1.53, 0.697, and 0.705 times different in *S. miltiorrhiza*, respectively (Supplementary Table 16). The expression level of *SmC4H1* in *S. miltiorrhiza* was higher at 0.83 times that of *S. castanea*. *SmC4H* may be related to the accumulation of salvianolic acid B in *S. castanea* (Supplementary Table 17).

To illustrate the differential mechanism of secondary metabolism between *S. castanea* and *S. miltiorrhiza*, we analyzed the expression of tanshinone and salvianolic acid biosynthesis-related genes at the transcriptional and proteomic levels. The results showed that 296 genes in *S. castanea* were upregulated at both the transcriptional and proteomic levels, while 125 genes in *S. miltiorrhiza* were significantly upregulated at both the transcriptional and protein levels. Further study revealed that the tanshinone synthesis-related genes *SmHDR1* (*comp19443_c0*) and *SmCPS1* (*comp22134_c0*) in *S. castanea* expressed 2.23- and 1.26-fold higher transcriptional and proteomic levels, respectively. *SmCPS1* expressed 3.61 and 1.53 times at transcriptional and proteomic levels as *S. miltiorrhiza* (Figure 2). Six differential CYP450 sequences were obtained through the combined analysis of the transcriptome and proteome. For instance, the expression levels of *CYP76AK1* in *S. miltiorrhiza* were both high at the transcriptional level and proteomic level, but *CYP71D411* catalyzed upstream hydroxylation at C20, and was 0.697 times downregulated in *S. castanea.*

**FIGURE 3:**
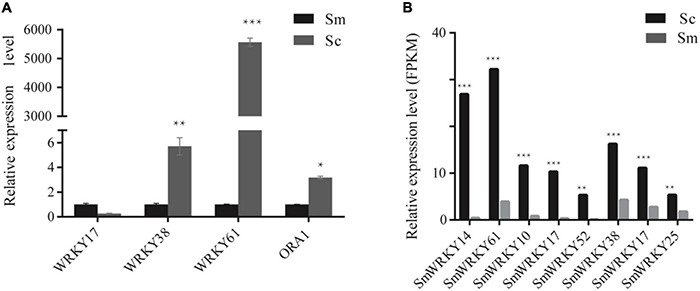
Relative expression level of *WRKYs* in *S. miltiorrhiza* and *S. castanea.* **(A)** RT-QPCR levels of WRKY transcription factors in hairy roots of *S. miltiorrhiza* and *S. castanea.* **(B)** Expression level of WRKY (*S. castanea*/*S. miltiorrhiza*) in *S. miltiorrhiza* and *S. castanea* transcriptomes. “*” represents 0.01 < *P* < 0.05, “**” represents *P* < 0.01, “***” represents *P* < 0.001.

Differentially expressed genes may be an important reason for the high accumulation of tanshinone in *S. castanea*. Among the above differentially expressed genes, we performed RT-qPCR analysis. The expression levels of four MYB genes such as *SmMYB42*, *SmMYB34*, *SmMYB107*, and *SmMYB85* were higher in *S. castanea*, which were 2.45, 4.24, 5.95, and 114.90 times higher than that in *S. miltiorrhiza*, respectively (Wang, 2019). The expression of *SmbHLH77* in *S. castanea* is 155.41 times higher than that in *S. miltiorrhiza* (Wang, 2019). At the same time, we also determined the expression of *WRKYs* by RT-qPCR. Among them, *SmWRKY17* was down expressed in *S. castanea*, and *SmWRKY38* was 5.7 times higher than *S. miltiorrhiza*. *SmWRKY61* was expressed 5,000 times more higher (Figure 3) in *S. castanea*, indicating that *SmWRKY61* was probably an important regulatory factor for high tanshinone accumulation in *S. castanea*. Moreover, *SmWRKY61* was highly expressed in the stem, and methyl jasmonate (MJ) can upregulate the expression of *SmWRKY61*, indicating that *SmWRKY61* participated in regulating tanshinone synthesis in *S. castanea*. Therefore, we infer that *SmWRKY61* is a potential important factor regulating the accumulation of tanshinone in *S. castanea*.

### Overexpression of *SmWRKY61* Can Promote Tanshinone Accumulation and Upregulate the Downstream Tanshinone Pathway Genes

**FIGURE 4:**
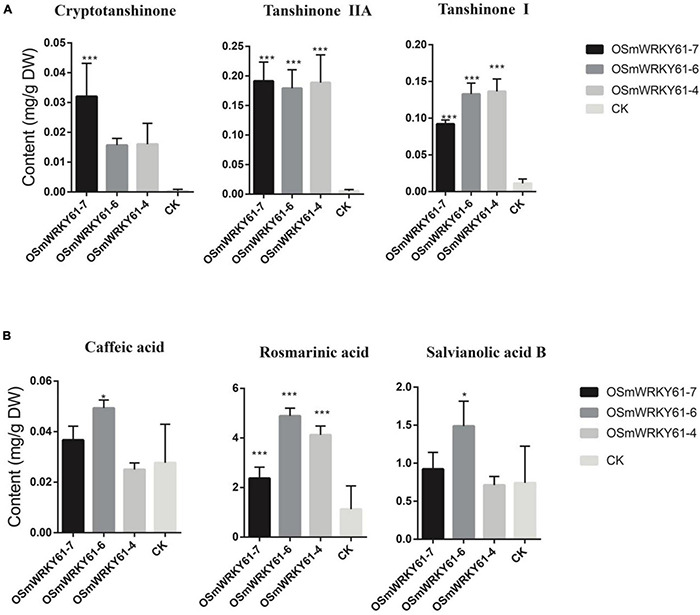
Tanshinones and polyphenolic compounds contents of hairy roots in *S. miltiorrhiza* and *S. castanea*. **(A)** HPLC analyse of tanshinones (cryptotanshinone, tanshinone IIA, and tanshinone I) in *S. miltiorrhiza* hairy roots. **(B)** HPLC analyse of phenolic acids (caffeic acid, rosmarinic acid, and salvianolic acid). “CK” represent *S. miltiorrhiza*, “*” represents 0.01 < *P* < 0.05, “**” represents *P* < 0.01, “***” represents *p* < 0.001. All data are the means of three replicates; error bars represent the indicate SD.

To reveal the role of *SmWRKY61* on tanshinone biosynthesis, we cloned and obtained the full-length sequence of *SmWRKY61*, and obtained *SmWRKY61* transgenic hairy root lines by overexpression. The leaves of the plants were infected with *Agrobacterium rhizogenes* ATCC15834, and the hairy roots were sampled after sterilization. The plant expression vector PK7WG2R was used to construct the overexpression vector by gateway technology to explore the function of the gene. PCR results showed that all the overexpressed strains were correctly inserted into the target gene (Supplementary Figure 5). In the *SmWRKY61*-overexpression hairy roots (O*SMWRKY61*), the weight of the hairy roots significantly increased at regular intervals (Supplementary Figures 6, 7). To analyze the role of *SmWRKY61* in secondary metabolism, the contents of three kinds of tanshinones (tanshinone I, cryptotanshinone, and tanshinone IIA) and three kinds of salvianolic acids (rosmarinic acid, caffeic acid, and salvianolic acid B) were analyzed by HPLC. The overexpression of *SmWRKY61* significantly promoted the accumulation of rosmarinic acid, tanshinone I, and tanshinone IIA, while cryptotanshinone was decreased (Figure 4A). Compared with the control, the rosmarinic acid in the O*SMWRKY61*-6 lines was increased by 3.33 times (control 1.13 mg.g^–1^,DW), the content of tanshinone IIA in the O*SMWRKY61*-4 lines was increased by 33.37 times (control 0.01 mg.g^–1^, DW), and the content of tanshinone I in the O*SMWRKY61*-7 lines was increased by 11.09 times (control 0.05 mg.g^–1^, DW). However, the contents of caffeic acid and salvianolic acid B were not significantly different. These results indicated that *SmWRKY61* significantly regulated the biosynthesis of tanshinone and rosmarinic acid. However, it seems that *SmWRKY61* was not involved in the biosynthesis of caffeic acid or salvianolic acid B.

**FIGURE 5:**
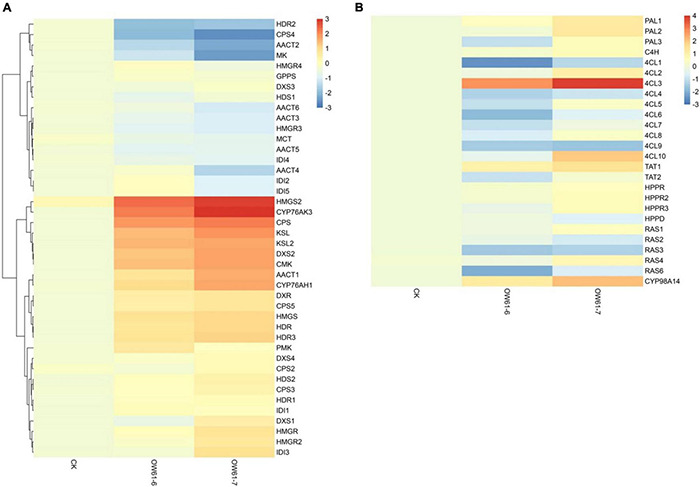
Genes expression in the biosynthesis of tanshinone and salvianolic acid in the *S. miltiorrhiza* hairy roots. **(A)** Gene expression of tanshinones biosynthesis in *S. miltiorrhiza* hairy roots. **(B)** Gene expression of phenolic acids biosynthesis in *S. miltiorrhiza* hairy roots. “CK” means the control group. The data were standardized, and the hierarchical clustering method was complete in R pheatmap.

Gene expression involving the tanshinone and salvianolic acid biosynthesis was analyzed through qRT-PCR (Figure 5). O*SMWRKY61*-6 and O*SMWRKY61*-7 were selected for gene expression analysis, and the quantitative results were plotted as log2. Figure 4 showed that both the salvianolic acid and tanshinone biosynthetic pathways could be regulated by *SmWRKY61.* Expression levels of genes in the downstream pathway of tanshinone biosynthesis were more significantly upregulated. For the phenolic acid biosynthesis, *SmWRKY61* mainly promoted the expression of *4Cl3*, *TAT1*, and *CYP98A14* in the downstream pathway. Compared to the control, the expression of *4Cl3* in the two overexpressed lines was increased by 23.7- and 73.6-fold, respectively. *TAT1* was increased by 3.1- and 5.8-fold, respectively and *CYP98A14* was increased by 3.8- and 11.3-fold, respectively.

The expression of key enzymes in the tanshinone metabolic pathway was significantly affected, including *DXS2, DXR, CMK, HDR*, and *HDR3* in the MEP pathway and *AACT1, HMGS*, and *HMGS2* in the MVA pathway. The expression of the downstream genes *IDI1, IDI3, CPS, CPS3, KSL, KSL2, CYP76AH1*, and *CYP76AK3* were upregulated. Among them, the expression levels of *DXS2, CMK, HMGS2, CPS, KSL, KSL2, CYP76AH1*, and *CYP76AK3* in OWRKY61-6 were 12.9, 13.3, 22.3, 17.2, 12.1, 12.4, and 59.0 times higher than that in the control, respectively.

As a result, *SmWRKY61* had a strong regulatory effect on tanshinone accumulation. *SmWRKY61* mainly promoted tanshinone accumulation by regulating the MEP pathway, especially the expression of related genes in the downstream pathway. Our study also found that *SmWRK61* significantly upregulated the expression of the *DXS2, CPS1*, and *CYP76AH1* genes, whereas expression of the *CPS1* and *HDR1* genes in *S. castanea* were significantly upregulated. These results indicated that the high expression of *SmWRK61* was probably the main reason for the accumulation of high levels of tanshinone in *S. castanea*.

### A More Specific Extension of *WRKY* Gene Family in *Salvia* Genus

**FIGURE 6:**
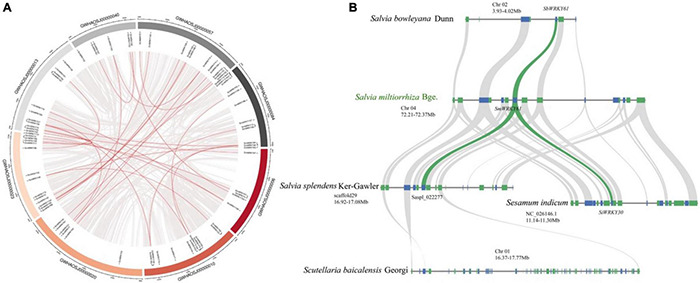
Collinearity analysis of *SmWRKYs*. **(A)** Collinearity analysis and chromosomes distribution of *SmWRKYs* in the *S. miltiorrhiza* genome. The gray lines mean all synteny blocks in the *S. miltiorrhiza* genome, and the red lines mean duplicated *WRKY* pairs in the *S. miltiorrhiza* genome. **(B)** Syntenic analysis of the *WRKY61* cluster by comparison to *S. indicum, S. miltiorrhiza, S. bowlevana*, *S. splendens*, and *S. baicalensis*.

To further reveal the regulatory effect of *SmWRKY61* on tanshinone accumulation, we identified the WRKY family in *S. miltiorrhiza*. A total of 79 candidate *SmWRKY* genes corresponding to the PF03106 were identified (Supplementary Table 18). Our phylogenetic analysis (Supplementary Figure 2) revealed that the *SmWRKYs* could be classified into three groups. *SmWRKY61* mainly belonged to WRKY family III, whose conserved motif was WRKYGQK, and the domain pattern was C-X5C-X22-HXC (Supplementary Figure 3A). The full length of *SmWRKY61* was 507 bp, encoding a 19.15 kD protein with an isoelectric point of 8.774. The secondary and tertiary structures are shown in Supplementary Figure 3B. Figure 6A showed that the *SmWRKY* were in homogeneously distributed on the 8 *S. miltiorrhiza* chromosomes. The segmental duplications play an important role in evolution, in which multiple genes through polyploidy were followed by chromosome rearrangements. Forty-eight *SmWRKY* genes were clustered into 30 segmental duplication event regions (Supplementary Table 19). However, *SmWRKY61* did not appear to be collinear with other members of the WRKY family.

The cluster of *SmWRKY61* had no homologs in *S. barcalensis* (Figure 6B), whereas three genes were orthologous to the two upstream and one downstream gene from *S. miltiorrhiza*, but no WRKY family members were present in this region. Moreover, the ortholog in *Sesamum indicum* (*SiWRKY30*) exhibited a more ancestral domain structure, which suggested that the domain mutation event may have occurred. *Saspl_022277* seemed to correlate with the *SmWRKY61* cluster in *S. splendens*. *S. bowlevana* also had collinear blocks with *S. miltiorrhiza*, and *SbWRKY61* had the highest homology with *SmWRKY61.* In conclusion, our collinearity results indicated a more specific extension of *WRKY* gene family in *Salvia* genus.

### Expression of *WRKY61* and Tanshinone Accumulation in *Salvia* L.

**FIGURE 7:**
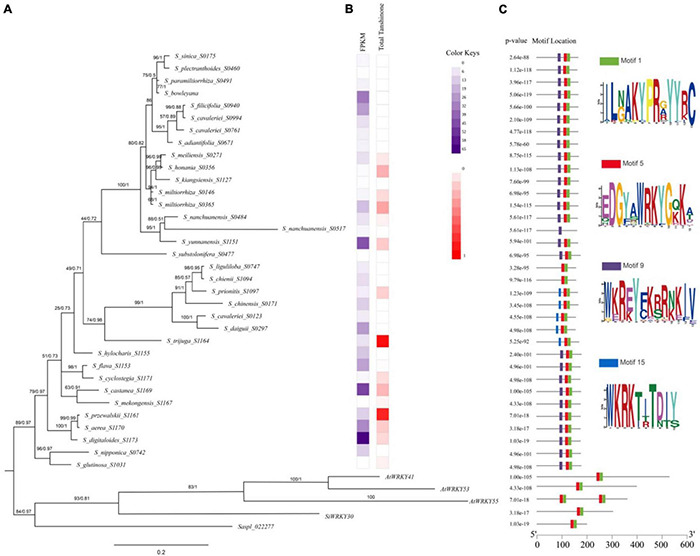
Phylogenetic relationships of conserved protein motifs in *WRKY* genes in *Salvia* L. **(A)** The genetic relationship of the phylogenetic tree constructed by Bayesian Inference (BI) method of *WRKY61* homologs in *Salvia* L. (Clades I–III). Numbers on the branches indicate statistical support values [numbers on left: maximum likelihood bootstrap proportion (ML-BP), numbers on right: Bayesian posterior probabilities (PP)]. **(B)** Heatmap of HPLC in *Salvia* L. dihydrotanshinone (DI), cryptotanshinone (CT), tanshinone I (TI), and tanshinone IIA (IIA). **(C)** The motif composition of *S. miltiorrhiza* WRKY proteins. The length of protein can be estimated using the scale at the bottom.

The expression of *SmWRKY61* was particularly high in *S. castanea* at both transcriptome level and qRT-PCR results. To further reveal the role of *WRKY61* in tanshinone biosynthesis in *Salvia* L., we analyzed *WRKY61* gene expression and tanshinone contents in transcriptomics of 55 *Salvia* L. worldwide, including East Asia (EA), Europe, and North America. The *WRKY61* sequence was detected only in EA *Salvia* L. The *WRKY61* was discovered in 37 representative species from 55 *Salvia* L. species (Figure 7A). The N-terminals of the WRKY domain contains seven landmark amino acid domains WRKYGQK (Figure 7C Motif 5), whereas WKRE mutation (Figure 7C Motif 9) and WKRK mutation (Figure 7C Motif 15) presented in the *Salvia* L. WKRE mutant domain appears in most *Salvia* L., except in the WKRK mutant domain branch. *WRKY61* was upregulated in *S_digitaloides_S1173, S_castanea_S1169, S_yunnanensis_S1151, S_bowleyana_S0603, S_aerea_S1170*, and *S_daiguii_S0297*, which indicates a highly analogical expression pattern of tanshinones. An aBSREL test found evidence of episodic diversifying selection on 2 out of 56 branches in the phylogeny. The fast unconstrained bayesian approximation test (FUBAR) (Murrell et al., 2013) indicated evidence of episodic positive/diversifying selection at 21 sites in 37 *WRKY61s*, with a posterior probability of 0.9 (Supplementary Table 23). Therefore, the above results indicated that an intense positive selection of *WRKY61* may take place in the evolutionary process of *Salvia* L.

A phylogenetic tree of *WRKY61* sequences from 37 representative species in 55 *Salvia* L. species (Supplementary Table 20) was constructed by Bayesian Inference (BI) performed by Mrbayes (Figure 7A). The results of the maximum likelihood (ML) analysis were largely consistent with the BI analysis. The phylogenetic relationships (Figure 7A) showed that *saspl_022277 and SiWRKY30*, which had high homology with *SmWRKY61*, clustered with *AtWRKY41*, *AtWRKY53*, and *AtWRKY55*. Within *Salvia* L., the three putative *WRKY61* gene groups (Clades I–III) were not distinguishable. In detail, Clade I comprised nine *Salvia* L. species (*S. glutinose* to *S. flava*), whereas Clade II included *S. hylocharis* to *S. liguliloba.* Clade III covered *S_substolonifera_S477* above, which together formed a likely sister group with clade II. The Clades I taxa belong to *Perennes* of subg. *Salvia sensu* (distributed in Lijiang, Yunnan, China), which was characterized by a Sino-Himalayan distribution pattern and tanshinone accumulation in the taxa. Particularly the expression level of *WRKY61* was also high in Clade I. *WRKY61* was first developed in *S_glutinosa*_S1031, but the expression of *WRKY61* in *S_glutinosa*_S1031 was downregulated. Taxa in clade I species: *S_digitaloides_S1173* (0.154 mg/g DW), *S_castanea_S1169* (0.287 mg/g DW), *S_przewalskii*_S1161 (0.864 mg/g DW), and *S_aerea*_S1170 (0.209 mg/g DW) showed high tanshinone accumulation and high *WRKY61* expression (Figure 7B). Six *WRKY61s* were detected in Clade II, belonging to *S. chinensis* group, which was placed in subg. *Allagospadonopsis.* In this clade, *S_trijuga_S1164* had the highest total tanshinone content in *Salvia* L.; subsequently, *S_prionitis*_S1097 had a total tanshinone content of 0.1895 mg/g (Figure 7B). However, no tanshinone was found in the other species in clade II. Phylogenetic and metabolomics evidence indicated that *S. trijuga* were members of subclade *Substoloniferae*, but the phylogenetic tree of *WRKY61* was placed in subg. *Allagospadonopsis.* Clade III contained 17 species in *Salvia*, which differed from the evolutionary relationships determined for *Salvia* L. In *S_nanchuanensis*_S0517 (*Salvia nanchuanensis* Sun var. *pteridifolia*), seven landmark domains WRKYGQK were replaced by WKRE mutations, which may have resulted in low expression of *WRKY61* and undetectable tanshinone content. Within the *Salvia miltiorrhiza* group, *S. meiliensis* and *S. honania* were two unique species that contain similar contents of tanshinone and *WRKY61* to *S. miltiorrhiza and S. kiangsiensis*.

## Discussion

Tanshinone has various pharmacological effects including cardioprotective (Wei et al., 2013; Wang et al., 2018), neuroprotective (Liu et al., 2010), antioxidant (Wang et al., 2013), anticancer (Chen et al., 2013), diabetic treatment (Lin et al., 2013), and other pharmacological effects. As far as we know, tanshinone was widely distributed in *Salvia* species (Wu et al., 2016). However, the biosynthesis and regulation mechanism of tanshinone in those species were largely unknown. In this work, the distribution pattern and accumulation of tanshinones in the roots of 37 *Salvia* species were analyzed by HPLC. We found that tanshinones were widely distributed in the roots of *Salvia* species from southwestern China such as *S. przewalskii*, *S. trijuga*, and *S. castanea.* Content of dihydrotanshinone I in *S. miltiorrhiza* roots was the highest, as high as 0.065% (Figure 1). The tanshinone IIA content of *S. miltiorrhiza* was only 0.2% (Figure 1). However, the content of tanshinone IIA in *S. castanea* was as high as 1.62% (Yang et al., 2012). To address the underlying reason for this difference, we performed comparative transcriptomic and proteomic analyses of *S. miltiorrhiza* and *S. castanea*. It was found that the key genes of tanshinone biosynthesis were upregulated in *S. castanea.* These results were consistent with our previous report (Fang et al., 2018). It suggested that the high expression of those key genes might be responsible for the high accumulation of tanshinones *S. castanea*. However, the underlying reasons causing the high expression of those key genes were still unclear.

A large number of studies have shown that transcription factors are important regulatory factors of plant secondary metabolism. Transcription factors can promote the accumulation of secondary metabolites by inducing expressions of multiple genes involved in biosynthetic pathways. With the development of third-generation sequencing technology, more than 1,300 TFs have been detected in *S. miltiorrhiza*, including WKRYs, bHLHs, MYBs and so on. Moreover, several TFs which can regulate tanshinone biosynthesis have been widely reported (Ding et al., 2017; Xing et al., 2018b; Zhang et al., 2020; Zhou et al., 2021). WRKY was another important transcription factor family involved in terpenoid biosynthesis. In *S. miltiorrhiza*, overexpression of *SmWRKY1* and *SmWRKY2* significantly increased the accumulation of tanshinone (Cao et al., 2018; Deng et al., 2019). In this work, we found dozens of differential expressed transcription factors like MYBs, bHLHs, and WRKYs. In these differentially expressed TFs, *SmWRKY61* expression was particularly high in *S. castanea*, and was 5,564 times more than that in *S. miltiorrhiza.* It was indicated that *WRKY61* was probably an important regulator for high tanshinone accumulation in *S. castanea*. So far, the roles of MYB and bHLH have been comprehensively reported in *Salvia miltiorrhiza*, but there are few reports on WRKY transcription factors. Therefore, we originally cloned a novel WRKY transcription factor *SmWRKY61* from *S. miltiorrhiza*. Overexpression of *SmWRKY61* in *S. miltiorrhiza* hairy roots can significantly enhance tanshinone accumulation. Contents of tanshinone IIA and tanshinone I in transgenic lines were 33.37 and 10.09 times higher than that in the control lines. Moreover, the promoting effect of *SmWRKY61* on tanshinone accumulation was significantly higher than that of other reported transcription factors, such as *SmMYB36* (Ding et al., 2017), *SmWRKY1* (Cao et al., 2018), *SmWRKY2* (Deng et al., 2019), *SmbHLH10* (Xing et al., 2018a), and *SmERF1L1* (Huang et al., 2019).

Perhaps more interestingly, we conducted a blast on NCBI with *WRKY61* and no homologous genes were found. *AtWRKY41*, *AtWRKY53*, and *AtWRKY55* had high homology with *SiWRKY30.* It was indicated that *WRKY61* had a unique structure in *Salvia* L. Collinearity analysis revealed that *S. barcalensis* did not have orthologs with *SmWRKY61*. However, the *SmWRKY61* ortholog in *Sesamum indicum* (*SiWRKY30*) exhibited the more ancestral and conservative WRKY domain structure. The collinearity analysis indicated a more specific origin of *WRKY61* in *Salvia* L. Within *Salvia* L., *WRKY61* genes were found in only 37 species of 55 *Salvia* L., and were only detected in EA *Salvia* L. FUBAR and aBSREL test found evidence of intense positive selection of *WRKY61*, which might take place in the evolutionary process of *Salvia* L. In particular, seven landmark domains WRKYGQK were replaced with WKRE mutation. It probably resulted in low expression of *WRKY61* and undetectable tanshinone content.

In conclusion, our study illustrated that *SmWRKY61* was a highly effective regulator of tanshinone accumulation and may be a key factor leading to high tanshinone accumulation in *S. castanea*. More specifically, the more specific *WRKY61* origin and evolutionary derivation of medically relevant tanshinones. Accordingly, our results provide insight into the contents of terpenoid differences and are more widely distributed in *Salvia* L.

## Materials and Methods

### Plant Materials

*Salvia miltiorrhiza* and *Salvia castanea* were planted in the greenhouse of Zhejiang Sci-Tech University (Hangzhou, Zhejiang, China). After growing for a year, the root of the plant was taken as a sample for transcriptomes and proteomes. The samples were immediately frozen in liquid nitrogen and stored in a −80°C freezer for future use. For the overexpression experiment, the aseptic leaves were infected with *Agrobacterium rhizogenes* strain ATCC 15834 to obtain *S. miltiorrhiza* hairy roots. The hairy roots were cultivated in 100-mL Erlenmeyer flasks, which contained 50 mL of 6, 7-V liquid medium (with 30 g l^–1^ sucrose). Adjusting the pH to 5.8, 0.2 g of fresh hairy roots were inoculated in each Erlenmeyer flask containing medium. Labeled samples were put in an orbital shaker (110 rpm) and incubated lucifugous at 25°C. After 24 days, to keep the roots at −80°C, our samples were rapidly frozen in liquid nitrogen. Portions of the samples were subsequently used to extract RNA and protein. All interaction experiments had three biological replicates.

### mRNA Library Construction and Sequencing

Total RNA was extracted using Trizol reagent (Invitrogen, CA, United States) following the manufacturer’s procedure using the root of annual plant *S. miltiorrhiza* and *S. castanea* roots. The total RNA quantity and purity were analyzed of Bioanalyzer 2100 and RNA 6000 Nano LaChhip Kit (Agilent, CA, United States) with RIN number >7.0. Approximately 10 μg of total RNA, representing a specific adipose type, was subjected to isolate poly (A) mRNA with poly-T oligo attached magnetic beads (Invitrogen). Following purification, the mRNA is fragmented into small pieces using divalent cations under elevated temperatures. Then the cleaved RNA fragments were reverse-transcribed to create the final cDNA library in accordance with the protocol for the mRNA-Seq sample preparation kit (Illumina, San Diego, CA, United States), the average insert size for the paired-end libraries was 300 bp (±50 bp). Then we performed the paired-end sequencing on an Illumina Hiseq2000 at the (LC Sciences, United States) following the vendor’s recommended protocol. A more detailed protocol of quality control, mapping, and sequence annotation methods can be found in Supplementary Method 1.1.

### Expression Profiling and Different Expression

To investigate the expression level of each unigene in different samples, all PE reads for each sample were aligned back to the final assembly by using perl scripts in Trinity under default parameters option. The alignment produced a digital expression level for each contig, and then these were normalized by RESM-based algorithm by using perl scripts in Trinity package to get RPKM values. Based on the expression levels, the significant differentially expressed transcripts (DETs) among different samples were identified with a *p* value < 0.05. The cluster of the DETs was performed by using the common perl and R scripts.

### Gene Family Collinearity and Chromosomal Distribution

The position of *SmWRKYs* on the chromosomes of *S. miltiorrhiza* was visualized by http://mg2c.iask.in/mg2c_v2.0/ (Chao et al., 2015; Supplementary Figure 4). All *SmWRKYs* were mapped to *S. miltiorrhiza* chromosomes based on physical location information from the *S. miltiorrhiza* genome using Circos (Krzywinski et al., 2009). MCScanX (Wang et al., 2012) was applied to analyze the *WRKY* gene collinearity, synteny, and duplication events. Whole-genome sequencing data of *S. miltiorrhiza* (PRJCA003150), *Salvia bowleyana* (PRJCA003734), *S. baicalensis* (PRJCA003374), *S. splendens* (GCA_004379255.1), and *S. indicum* (GCA_001692995.1) were downloaded from the Genome Warehouse of the National Genomics Data Center. A more detailed gene family identification and sequence analysis method can be found in Supplementary Method 1.2.

### *WRKY61* Gene Identification and Sequence Analysis in *Salvia* L.

WRKY61 gene in *Salvia* L. *S_cavaleriei*_S0123, *S_daiguii*_S0297, *S_liguliloba*_S0747, *S_chienii*_S1094, *S_prionitis*_S1097, *S_chinensis*_S0171, *S_hylocharis*_S1155, *S_flava*_S1153, *S_cyclostegia*_S1171, *S_przewalskii*_S1161, *S_aerea*_S1170, *S_digitaloides*_S1173, *S_mekongensis*_S1167, *S_castanea*_S1169, *S_nipponica*_S0742, *S_glutinosa*_S1031, *S_grandifolia*_S0804, *S_splendens*_S1062, *S_substolonifera*_S0477, *S_adiantifolia* _S0671, *S_cavaleriei*_S0761, *S_filicifolia*_S0940, *S_cava leriei*_S0994, *S_nanchuanensis*_S0484, *S_yunnanensis*_S1151, *S_nanchuanensis*_S0517, *S_miltiorrhiza*_S0146, *S_miltiorrhiza*_ S0365, *S_sinica*_S0175, *S_plectranthoides*_S0460, *S_paramil tiorrhiza*_S0491, *S_meiliensis*_S0271, *S_honania*_S0356, *S_kiangsiensis*_S1127, *S_trijuga*_S1164, *S_coccinea*_S0318, *S_adenophora*_S1068 were obtained from transcriptome, which is listed in Supplementary Table 20. *AtWRKY41*, *AtWRKY53*, and *AtWRKY55* were selected as an outgroup. A phylogeny etic tree was constructed based on nucleotide sequence multiple alignments used MAFFT (Katoh and Standley, 2013) and IQTREE2 (Nguyen et al., 2015) with the JTT + G4 model 5,000 replicates bootstrap. Mrbayes version 3.1.2 (Fredrik and John, 2003) was adopted to analyze Bayesian inference (BI) with the GTR + G model, where model was selected by jmodeltest (Posada, 2008) under the Akaike information criterion (AIC). The convergence of the analyses was validated by the standard deviation of split frequencies (<0.01). The first 25% of generations were discarded as burn-in. A 50% majority-rule consensus tree was constructed from the remaining trees to estimate posterior probabilities (PPs) of nodal support. Diversifying selections were formally tested at the 56 branches at http://datamonkey.org/ (Weaver et al., 2018) using aBSREL test (Smith et al., 2015; Supplementary Table 22). Fast unconstrained Bayesian approximation test (FUBAR) (Murrell et al., 2013) found evidence of episodic positive/diversifying selection at 21 sites and episodic negative/purifying selection at 1 site with a posterior probability of 0.9 (Supplementary Table 23).

### Protein Extraction

The samples were ground to a powder in liquid nitrogen, extracted with lysis buffer containing 1 mM PMSF and 2 mM EDTA (final concentration). Then 10 mM DTT (final concentration) was added, and the samples were sonicated at 200 W for 15 min and then centrifuged at 4°C for 15 min. The supernatant was mixed well with acetone containing 10% (v/v) TCA and incubated at −20°C overnight. After centrifugation at 4°C, 30,000 *g*, the supernatant was discarded. The precipitate was washed with chilled acetone three times. The pellet was air-dried and dissolved in lysis buffer. The suspension was sonicated at 200 W for 15 min and centrifuged at 4°C, 30,000 *g* for 15 min. The supernatant was transferred to another tube. To reduce disulfide bonds in proteins of the supernatant, 10 mM DTT (final concentration) was added and incubated at 56°C for 1 h. Subsequently, 55 mM IAM (final concentration) was added to block the cysteines and incubated for 1 h in the darkroom. The supernatant was mixed well with 5 volumes of chilled acetone for 2 h at −20°C to precipitate proteins. After centrifugation at 4°C and 30,000 *g*, the supernatant was discarded, and the pellet was air-dried for 5 min, dissolved in TEAB (Applied Biosystems, Milan, Italy), and sonicated at 200 W for 15 min. Finally, samples were centrifuged at 4°C, 30,000 *g* for 15 min. The supernatant was transferred to a new tube and quantified. The proteins in the supernatant were kept at −80°C for further analysis.

### Isobaric Tags for Relative and Absolute Quantification Labeling and SCX Fractionation

Total protein (100 g) was taken out of each sample solution and then the protein was digested with trypsin Gold (Promega, Madison, WI, United States) with the ratio of protein: trypsin = 30:1 at 37°C for 16 h. After trypsin digestion, peptides were dried by vacuum centrifugation. Peptides were reconstituted in 0.5*M* TEAB and processed according to the manufacturer’s protocol for 8-plex iTRAQ reagent (Applied Biosystems). Briefly, one unit of iTRAQ reagent was thawed and reconstituted in 24 μl isopropanol. Samples were labeled with the iTRAQ tags as follows: Sample *S. miltiorrhiza*, Sample *S. castanea*. The peptides were labeled with the isobaric tags, incubated at room temperature for 2 h. The labeled peptide mixtures were then pooled and dried by vacuum centrifugation. SCX chromatography was performed with a LC-20AB HPLC Pump system (Shimadzu, Kyoto, Japan). The iTRAQ-labeled peptide mixtures were reconstituted with 4 mL buffer A (25 mM NaH2PO4 in 25% ACN, pH 2.7) and loaded onto a 4.6 × 250 mm Ultremex SCX column containing 5-μm particles (Phenomenex). The peptides were eluted at a flow rate of 1 mL/min with a gradient of buffer A for 10 min, 5–60% buffer B for 27 min, 60–100% buffer B for 1 min. The system was then maintained at 100% buffer B for 1 min before equilibrating with buffer A for 10 min prior to the next injection. Elution was monitored by measuring the absorbance at 214 nm, and fractions were collected every 1 min. The eluted peptides were pooled into 20 fractions, desalted with a Strata X C18 column (Phenomenex), and vacuum dried.

### LC-ESI-MS/MS Analysis

Each fraction was resuspended in buffer A (5% ACN, 0.1%FA) and centrifuged at 20,000*g* for 10 min, the final concentration of peptide was about 0.5 g/l on average. A total of 10l supernatants were loaded on a LC-20AD nano HPLC (Shimadzu, Kyoto, Japan) by the autosampler onto a 2-cm C18 trap column. Then, the peptides were eluted onto a 10-cm analytical C18 column (inner diameter 75 m) packed in-house. The samples were loaded at 8 L/min for 4 min, then the 35 min gradient was run at 300 nl/min starting from 2 to 35% B (95%ACN, 0.1%FA), followed by 5 min linear gradient to 60%, then followed by 2 min linear gradient to 80%, maintained at 80% B for 4 min, and finally returned to 5% in 1 min. Data acquisition was performed with a TripleTOF 5600 System (AB SCIEX, Concord, ON) fitted with a Nanospray III source (AB SCIEX, Concord, ON) and a pulled quartz tip as the emitter (New Objectives, Woburn, MA, United States). Data were acquired using an ion spray voltage of 2.5 kV, curtain gas of 30 psi, nebulizer gas of 15 psi, and an interface heater temperature of 150. The MS was operated with a RP of greater than or equal to 30 000 FWHM for TOF MS scans. For IDA, survey scans were acquired in 250 ms and as many as 30 product ion scans were collected if exceeding a threshold of 120 counts per second (counts/s) and with a 2+ to 5+ charge-state. The total cycle time was fixed to 3.3 s. Q2 transmission window was 100 Da for 100%. Four times bins were summed for each scan at a pulser frequency value of 11 kHz through monitoring of the 40 GHz multichannel TDC detector with four-anode channel detection. A sweeping collision energy setting of 35 ± 5 eV coupled with iTRAQ adjust rolling collision energy was applied to all precursor ions for collision-induced dissociation. Dynamic exclusion was set for 1/2 of peak width (15 s), and then the precursor was refreshed off the exclusion list. A more detailed data analysis method can be found in Supplementary Method 1.3.

### *WRKY* Identification and Functional Validation Experiments

*SmWRKY61* (KM823184.1) protein sequences were downloaded from Genbank. The CDS of *SmWRKY61* was amplified and cloned (Supplementary Figures 4A,B) into the restriction site *att*P of the pDONR207 vector. The overexpression vector was constructed by Gateway method. A DNA fragment with *att*B-flanked was subjected to a BP recombination reaction with an *att*P-containing donor vector to generate an entry clone (Supplementary Figure 6C and Supplementary Table 25). The entry clone *att*L-containing is subjected to LR recombination reaction with the target vector *att*R-containing to generate an expression clone into the pK7WG2R vector (Supplementary Figure 5D and Supplementary Table 26). The contain recombinant plasmid *SmWRKY61* was transformed into *A. rhizogenes* (ATCC15834) (Supplementary Figure 5E). The *rolB, rolC, HPT*, and *SmWRKY61* specific primers were used to identify positive transgenic lines. Our transgenic lines were used for RNA extraction, and they were regularly subcultured (every 30 days) (Supplementary Figure 6). All the above primers are listed in Supplementary Table 24.

GoTaq-qPCR Master Mix kit (Promega, China) was used to perform qRT-PCR assay on an Applied Biosystems StepOne Real-time PCR System (United States) with *Actin* gene as the internal control, where the primers are listed in Supplementary Table 27. The comparative *Ct* method was performed to quantify gene expression levels in three biological replicates.

### HPLC Analysis to Measure the Phenolic Acid and Tanshinone Contents

Fresh hairy roots were harvested and dried and then ground to a powder. A total of 200 mg fresh samples were extracted with 16 mL of methanol/dichloromethane (3:1, v/v). Then the samples were sonicated for 1 h and centrifuged at 25°C. The Waters E2695 binary high-performance liquid chromatograph was used for content determination, and the detector was Waters 2996 diode array detector. Waters Sunfire C18 column (250 mm × 4.6 mm, 5 μm) was used for chromatographic analysis. Data collection software Empower 3 was used for data collection. The chromatographic conditions were as follows: flow rate of 1 mL/min, column temperature of 30°C, sample loading volume of 10 l. The absorbance was at 270 nm (tanshinone) and 288 nm (phenolic acid), respectively, and the mobile phases were 0.026% phosphoric acid aqueous solution and acetonitrile, respectively, with gradient elution. By plotting the peak area (y) of the analyte and the corresponding concentration (*x*, mg mL^–1^), the linear relationship of each standard curve was determined. The regression equation and correlation coefficient are [*y* = 3,724,065.1501*x*–86,580.8597] (*R*^2^ = 0.9999) for tanshinone I, [y = 5,524,625.7662*x* + 78,811.1024] (*R*^2^ = 0.9988) for cryptotanshinone, [*y* = 2,660,430.0088*x* + 22,146.7859] (*R*^2^ = 0.9997) for dihydrotanshinone I and [*y* = 5,128,785.8762*x*–58,317.4739] (*R*^2^ = 0.9994) for tanshinone IIA. [*y* = 5472992.9477*X*−3794.4223] (*R*^2^ = 1) for caffeic acid, [*y* = 1939973.1010*X* + 239.4423] (*R*^2^ = 0.9999) for rosmarinic acid and [*y* = 960469*X*-63957] (*R*^2^ = 0.9999) for salvianolic acids B. The CAS of standards for quantification by HPLC was listed in Supplementary Table 28.

### Quantitative Real-Time-PCR

A total of 1 μg of RNA was reverse transcribed for first-strand cDNA synthesis using the Prime Script™ RT reagent Kit (Takara, Japan) according to the manufacturer’s instructions. Reactions were performed with the SYBR Green PCR Master Mix in Applied Biosystems by Life Technologies (QuantStudio 6 Flex, ABI, Waltham, MA, United States). *Actin* was used as the standard to normalize the content of cDNA. Ten microliters of the reaction mixture was added to each well. The thermal cycling program was set at 40 cycles of 95°C for 30 s, 95°C for 5 s, and 59°C for 30s. Relative quantification of gene expression levels was dealt with the comparative CT method (2^–ΔΔCT^). Adhering to minimal MIQE guidelines, RT-qPCR was carried out using *SmActin* gene as reference gene, primer as previously reported (Zhang et al., 2020). The real-time PCR was conducted with three replicates for each sample, and data are indicated as means ± standard error (SE) (*n* = 3).

## Data Availability Statement

The datasets presented in this study can be found in online repositories. The names of the repository/repositories and accession number(s) can be found in the article/Supplementary Material.

## Author Contributions

ZL, LH, DY, and YWa conceived and organized the experiments. YuC and YWe performed the experiments. YuC, YiC, ZD, ZW, ZQ, and MD performed the bioinformatics analysis. DY, ZL, YH, JG, JY, and XZ contributed to the data analysis. YuC and DY wrote the manuscript. All the authors discussed and approved the final manuscript.

## Conflict of Interest

The authors declare that the research was conducted in the absence of any commercial or financial relationships that could be construed as a potential conflict of interest.

## Publisher’s Note

All claims expressed in this article are solely those of the authors and do not necessarily represent those of their affiliated organizations, or those of the publisher, the editors and the reviewers. Any product that may be evaluated in this article, or claim that may be made by its manufacturer, is not guaranteed or endorsed by the publisher.
