# Supplementary material for: Integrated Transcriptomics and Proteomics to Reveal Regulation Mechanism and Evolution of SmWRKY61 on Tanshinone Biosynthesis in Salvia miltiorrhiza and Salvia castanea
Source: Front Plant Sci. 2022 Mar 3;12:820582. doi: 10.3389/fpls.2021.820582 (PMC8928407; doi:10.3389/fpls.2021.820582)
Supplement: Supplementary file 1 [file Data_Sheet_1.docx]

Supplementary Material

1 Supplementary Methods

## 1.1 Quality control, mapping and sequence annotation

A cDNA library was constructed by de novo technology from the pooled RNA, the root samples of *S. miltiorrhiza* and *S. castanea* were sequenced and run with Illumina 2100 sequence platform. Using the Illumina paired-end RNA-seq approach, we sequenced the transcriptome, generating a total of 4.5G for *S. miltiorrhiza* and 5.39G for *S. castanea* paired-end reads of 30,027,614 bp for *S. miltiorrhiza* and 35,952,050 for *S. castanea* length. FastQC (Ward *et al.*, 2018) was selected to filter low-quality sequences. 29,644,212 reads for *S. castanea* and 35,421,130 reads for *S. miltiorrhiza* clean data were obtained by removing reads containing adapter, reads containing ploy-N and low-quality reads from raw data. At the same time, Q20, Q30, GC-content and sequence duplication levels of the clean data were calculated. All the downstream analyses were based on clean data with high quality. We aligned reads of *S. miltiorrhiza* and *S. castanea* were de novo assembled by using Trinity (Grabherr *et al.*, 2013) under default parameters choice. Assembly quality was assessed by length distribution analysis by common perl scripts. N50 number, average length, max length and contig number during different length interval were all been calculated. Moreover, we scanned the best candidate coding sequence (CDS) for each contig and got the ratios of long-CDS containing transcripts to corresponding length contigs.

Unigene annotations provide functional annotations for all unigenes, along with their expression levels. Functional annotations of unigenes were analyzed using protein sequence similarity, KEGG Pathway, KOG and GO analysis. All unigene sequences were against the protein databases (Nr, Swiss-Prot, KEGG, KOG) using BLASTx (E-value<0.00001) (Altschul *et al.*, 1997). Protein function information could be predicted from annotations of the most similar proteins in the databases. The KEGG pathway database records networks of molecular interactions in the cells, and variants of these pathways are specific to organisms (Ogata *et al.*, 1999). KOG is a database where orthologous gene products are classified. All unigenes were aligned to the KOG database to predict and classify their possible functions. GO functional annotation was obtained from SwissProt annotation. GO annotation comprises three ontologies, i.e., a molecular function, a cellular component and a biological process. The basic GO unit is a GO-term, and every GO-term belongs to a type of ontology.

### 1.2 Gene family identification and sequence analysis

In order to avoid missing potential WRKY genes, Pfamscan based on HMMER suite (http:// hmmer.janelia.org/) was used to screen candidate *WRKYs* from the predicted *S. miltiorrhiza* proteome (Song *et al.*, 2020), using the Pfam profiles of PF03106 as queries (E value < 10 -10). To further verify the annotation of the *SmWRKYs* prediction models, we used Tophat2 (Kim *et al.*, 2013) and Cufflinks (Cole *et al.*, 2012) to map the RNA-seq sequence to the *S. miltiorrhiza* genome (Song *et al.*, 2020). A total of 79 such full-length *SmWRKYs* were identified (Table S18). After multiple protein sequence alignments using MAFFT (Katoch *et al.*, 2013), maximum likelihood trees were built using IQTREE2 (Nguyen *et al.*, 2015) with the JTT+F+R7 model using 10,000 bootstrap replicates. Then, we use the protein sequence analysis MEME program (http://meme.nbcr.net/meme/intro.html) to identify the conserved motifs of the identified WRKY proteins (Bailey *et al.*, 2009). We used swissmodel (https://swissmodel.expasy.org/interactive/f Tm NPw/models/) and (https://swissmodel.expasy.org/interactive) to predict *SmWRKY61* secondary and tertiary structure prediction. Protein subcellular localization used Protein Predict software (https://www.predictprotein.org/).

## 1.3 Data Analysis

Raw data files acquired from the Orbitrap were converted into MGF files using Proteome Discoverer 1.2 (PD 1.2, Thermo), [5600 msconverter] and the MGF file were searched. Protein identifications were performed by using Mascot search engine (Matrix Science, London, UK; version 2.3.02) against customers provided database containing 18305 sequences. The quantitative protein ratios were weighted and normalized by the median ratio in Mascot. We only used ratios with p-values < 0.05, and only fold changes of >1.2 were considered as significant.

2 Supplementary reference

Altschul, S. F., Madden, T. L., Schffer, A. A., Zhang, J., Zhang, Z., & Webb, M., *et al*. (1997). Gapped blast and psi-blast: a new generation of protein database search programs. Nucleic acids research, 25(17), 3389.

Bailey, T. L., Boden, M., Buske, F. A., Frith, M., Grant, C. E., Clementi, L *et al*. (2009). MEME Suite: tools for motif discovery and searching. *Nucleic Acids Research*. 37, 202-208. doi: 10.1093/nar/gkp335

Cole, T., Adam, R., Loyal, G., Geo, P., Daehwan, K., David R, K., *et al*. (2012). Differential gene and transcript expression analysis of RNA-seq experiments with TopHat and Cufflinks. *Nature protocols*. 7:3. doi: 10.1038/nprot.2012.016

Grabherr, M. G., Haas, B. J., Yassour, M., Levin, J. Z., & Amit, I. (2013). Trinity: reconstructing a full-length transcriptome without a genome from rna-seq data. Nature Biotechnology, 29, 644.

Katoh, K., and Standley, D. M. (2013). MAFFT multiple sequence alignment software version 7: improvements in performance and usability. *Mol. Biol. Evol.* 30, 722-780

Kim, D., Pertea, G., Trapnell, C., Pimentel, H., Kelley, R., Salzberg, S. L. (2013). TopHat2: accurate alignment of transcriptomes in the presence of insertions, deletions and gene fusions. *Genome Biol*. 14:4, R36. doi: 10.1186/gb-2013-14-4-r36

Nguyen, L. T., Schmidt, H. A., von Haeseler, A., and Minh, B. Q. (2015). IQ-TREE: a fast and effective stochastic algorithm for estimating maximum-likelihood phylogenies. *Molecular biology and evolution*. 32:1, 268-274. doi: https://doi.org/10.1093/molbev/msu300

Ogata, H., Goto, S., Sato, K., Fujibuchi, W., & Kanehisa, M. (1999). Kegg: kyoto encyclopedia of genes and genomes. Nucleic Acids Research, 27(1), 29-34.

Song, Z., Lin, C., Xing, P., Fen, Y., Jin, H., Zhou, C., *et al*. (2020). A high-quality reference genome sequence of *Salvia miltiorrhiza* provides insights into tanshinone synthesis in its red rhizomes. *The Plant Genome*. 13:3. doi: https://doi.org/10.1002/tpg2.20041

Ward, C. M., To, T. H., & Pederson, S. M. (2018). Ngsreports: an r package for managing fastqc reports and other ngs related log files. Bioinformatics.

3 Supplementary Figures

#
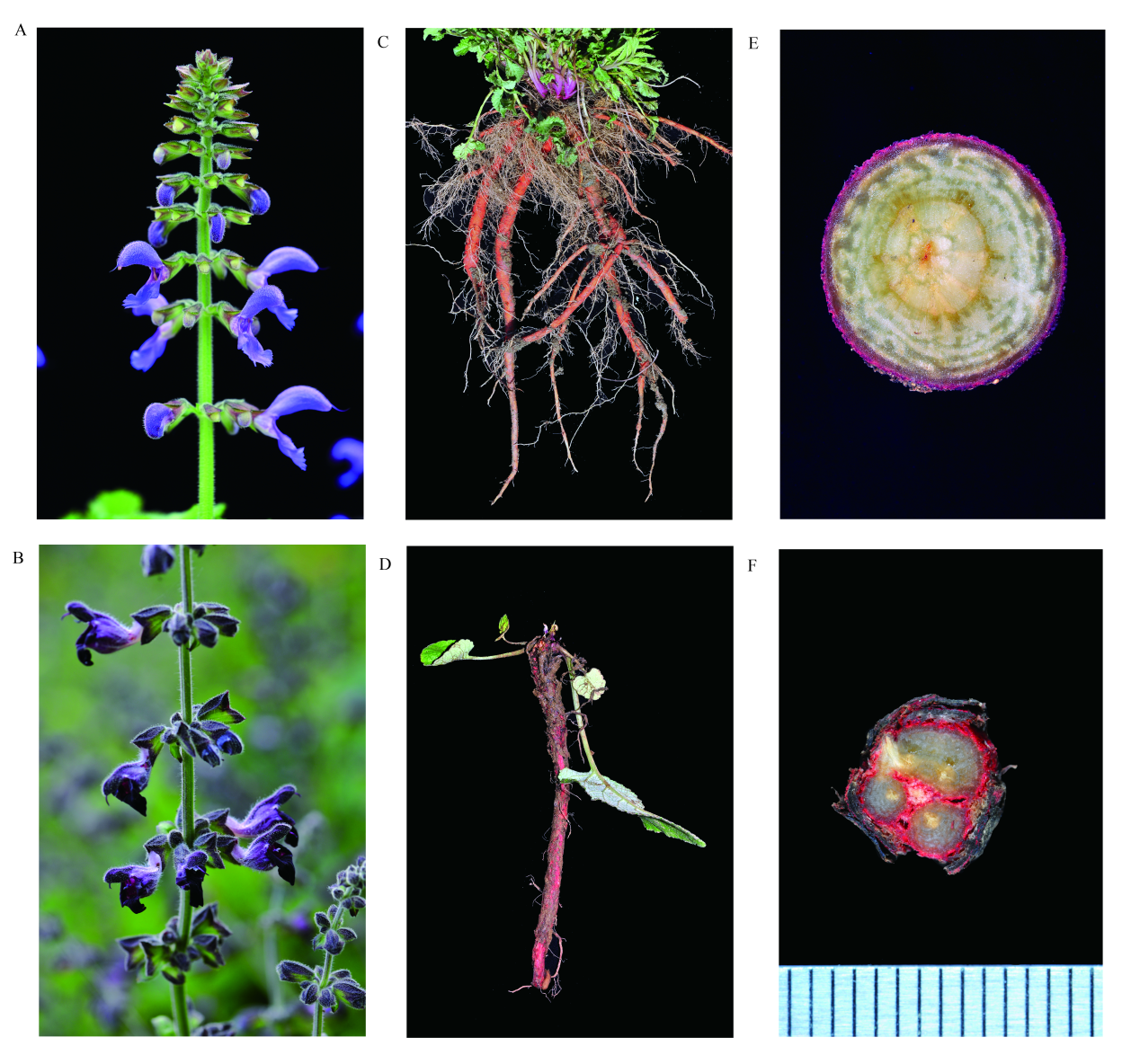
Figure S1. Differences in the morphological characteristics of *S. miltiorrhiza* and *S. castanea*

1. The inflorescence of *S. miltiorrhiza*.
2. The inflorescence of *S. castanea.*
3. Colouration of *S. miltiorrhiza* roots*.*
4. Colouration of *S. castanea* roots*.*
5. Cross-section of *S. miltiorrhiza* roots.
6. Cross-section of *S. castanea* roots.

**
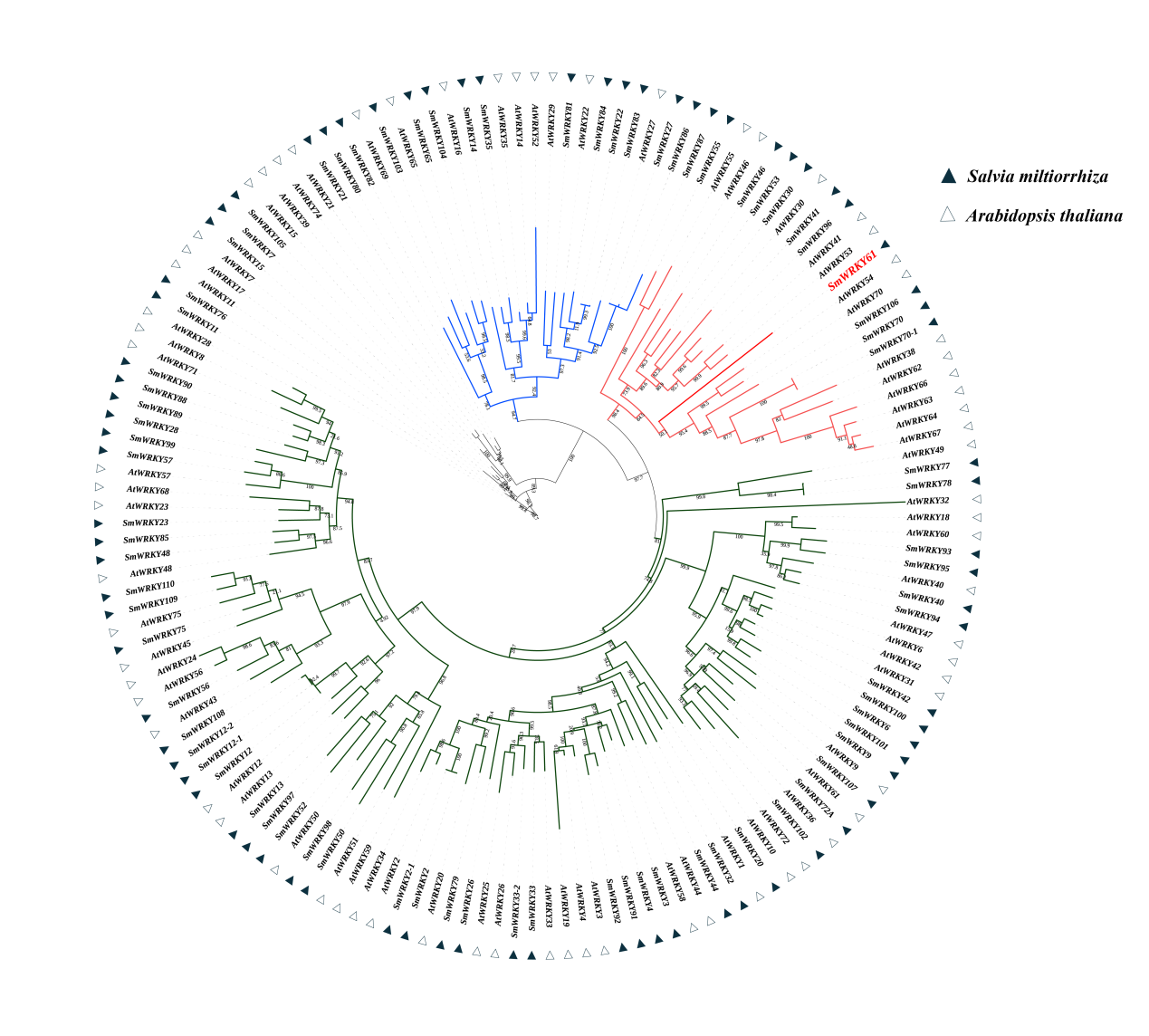
**

# Figure S2 Unrooted phylogenetic tree representing relationships among WRKY domains of *S. miltiorrhiza* and *Arabidopsis*.

The different-colored arcs indicate different groups (or subgroups) of WRKY domains. The black solid circles and hollow circles represent WRKY domain from *S. miltiorrhiza* and *Arabidopsis*, respectively.

#
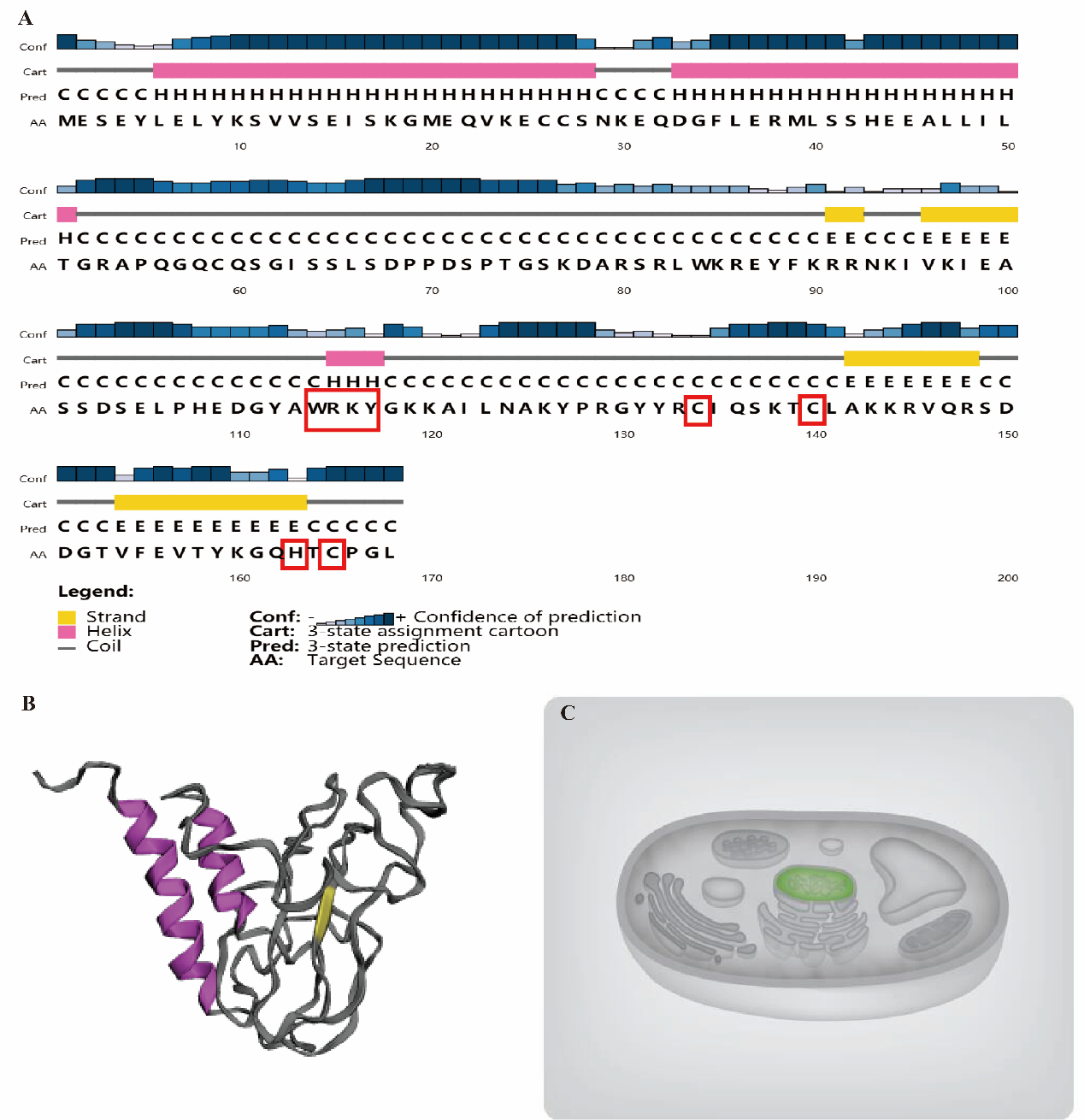
Figure S3. *SmWRKY* sequence, protein structure prediction and subcellular localization

1. Secondary structure prediction**.** The red box indicates *SmWRKY61* domain pattern C-X5C-X22-HXC
2. Protein structure prediction of *SmWRKY61*
3. Subcellular locations of *SmWRKY61*

#
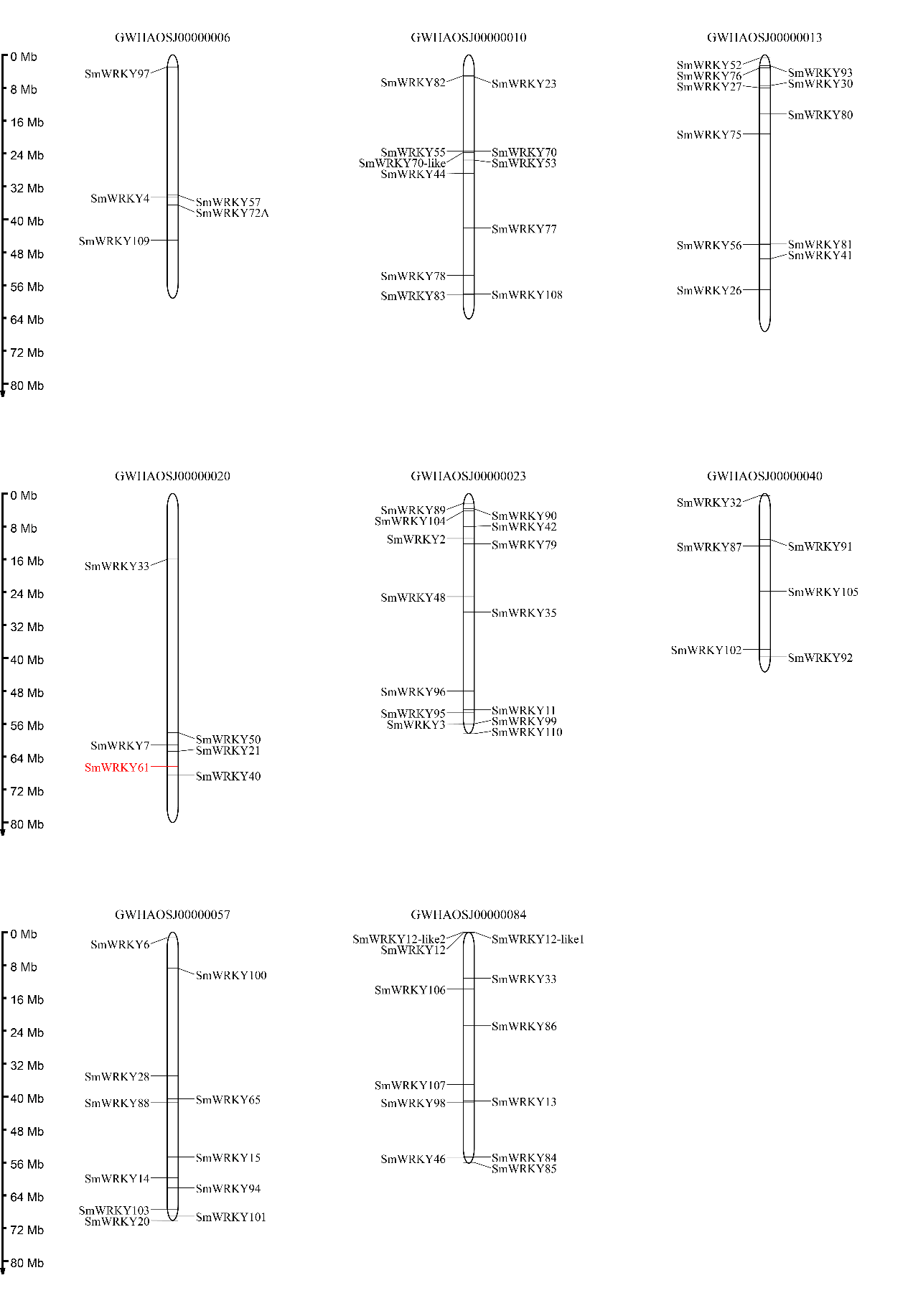
Figure S4. The position of WRKY genes on the chromosomes of *S. miltiorrhiza*

#
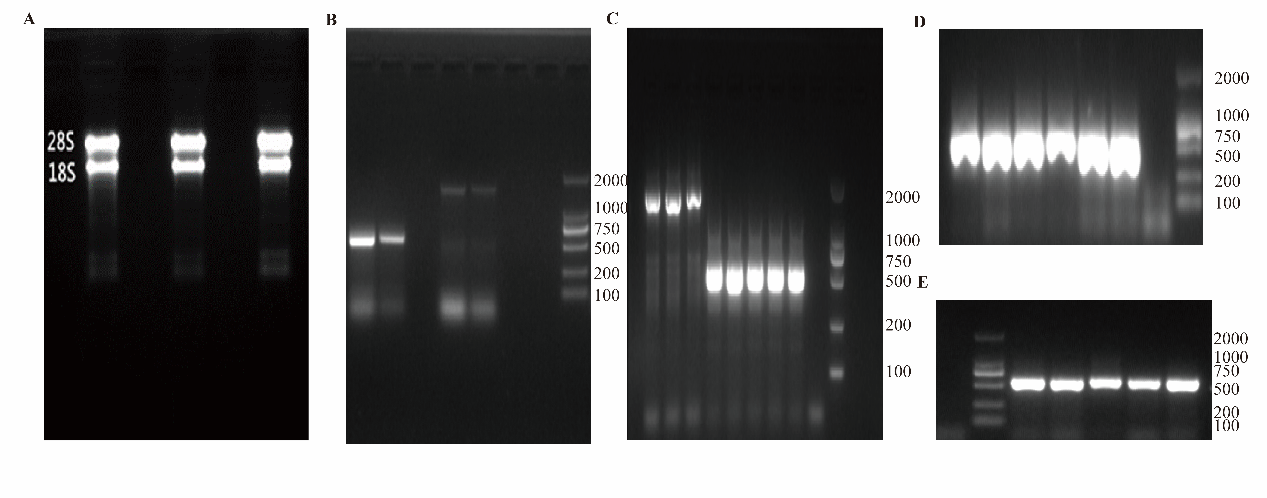
Figure S5. PCR identification of BP and LR reaction

1. Total RNA electrophoresis of hairy roots of *S. miltiorrhiza*
2. *SmWRKY61* gene amplification electrophoresis
3. (D) Agarose gel electrophoresis map of identification of positive clones of *Agrobacterium rhizogenes*

(E) Identification of positive transgenic hairy root lines by PCR

#
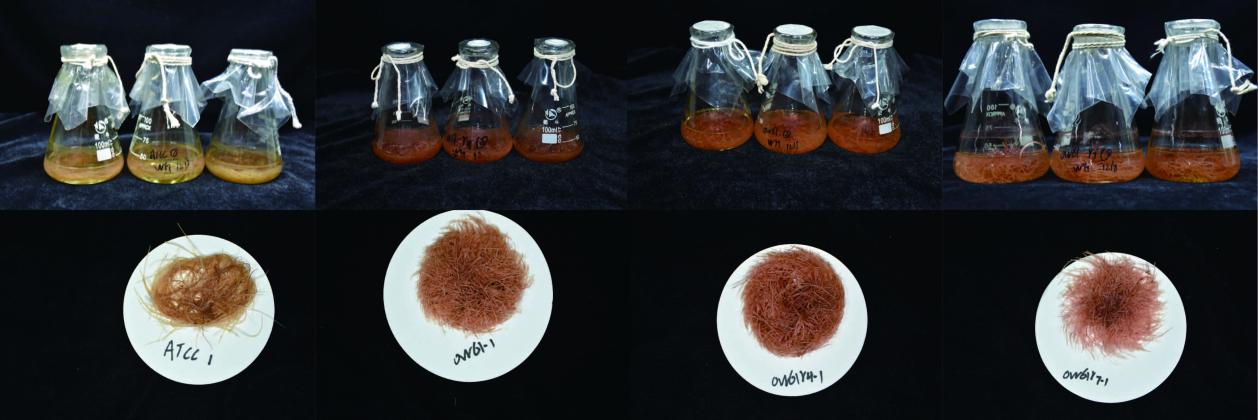
Figure S6. *SmWRKY* over-expression hairy root lines

#
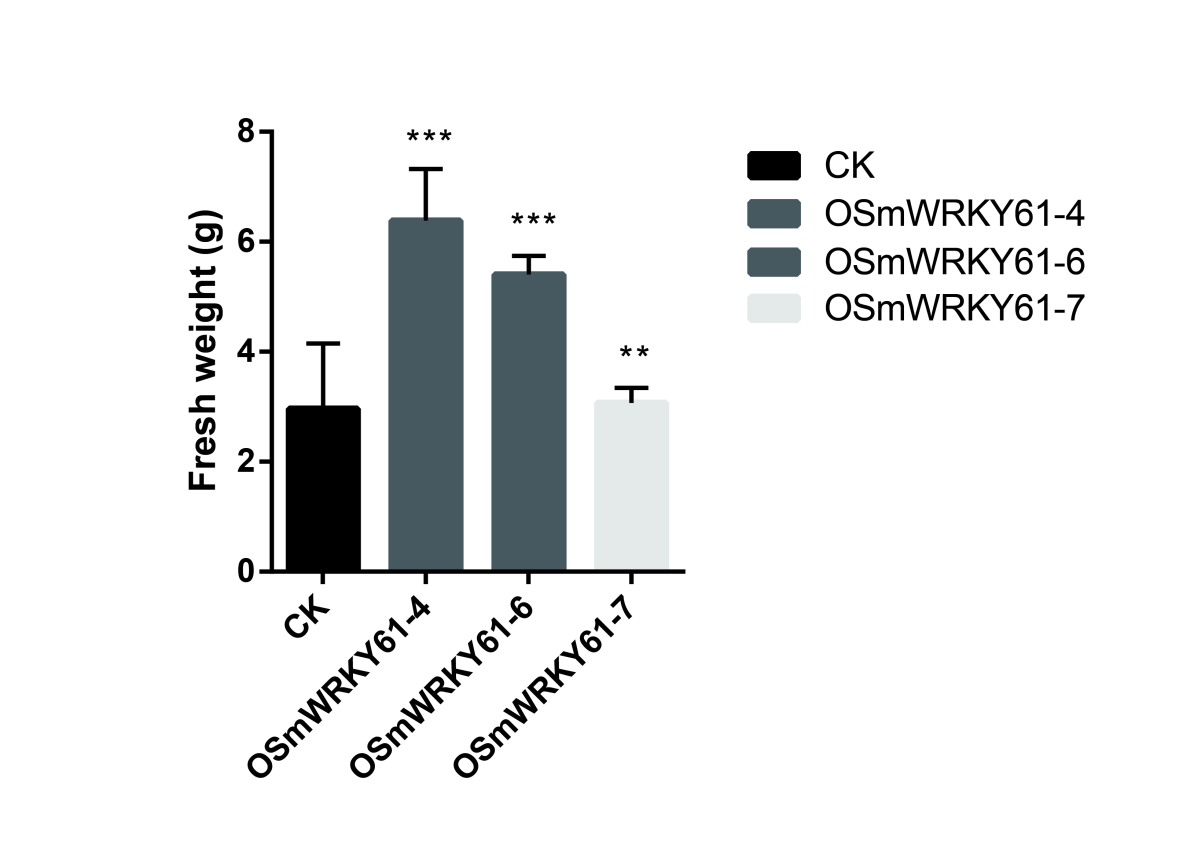
Figure S7. The biomass of *SmWRKY* over-expression hairy root

“*” represents 0.01<*P*<0.05, “**” represents *P*<0.01. All data are the means of three replicates; error bars indicate SD


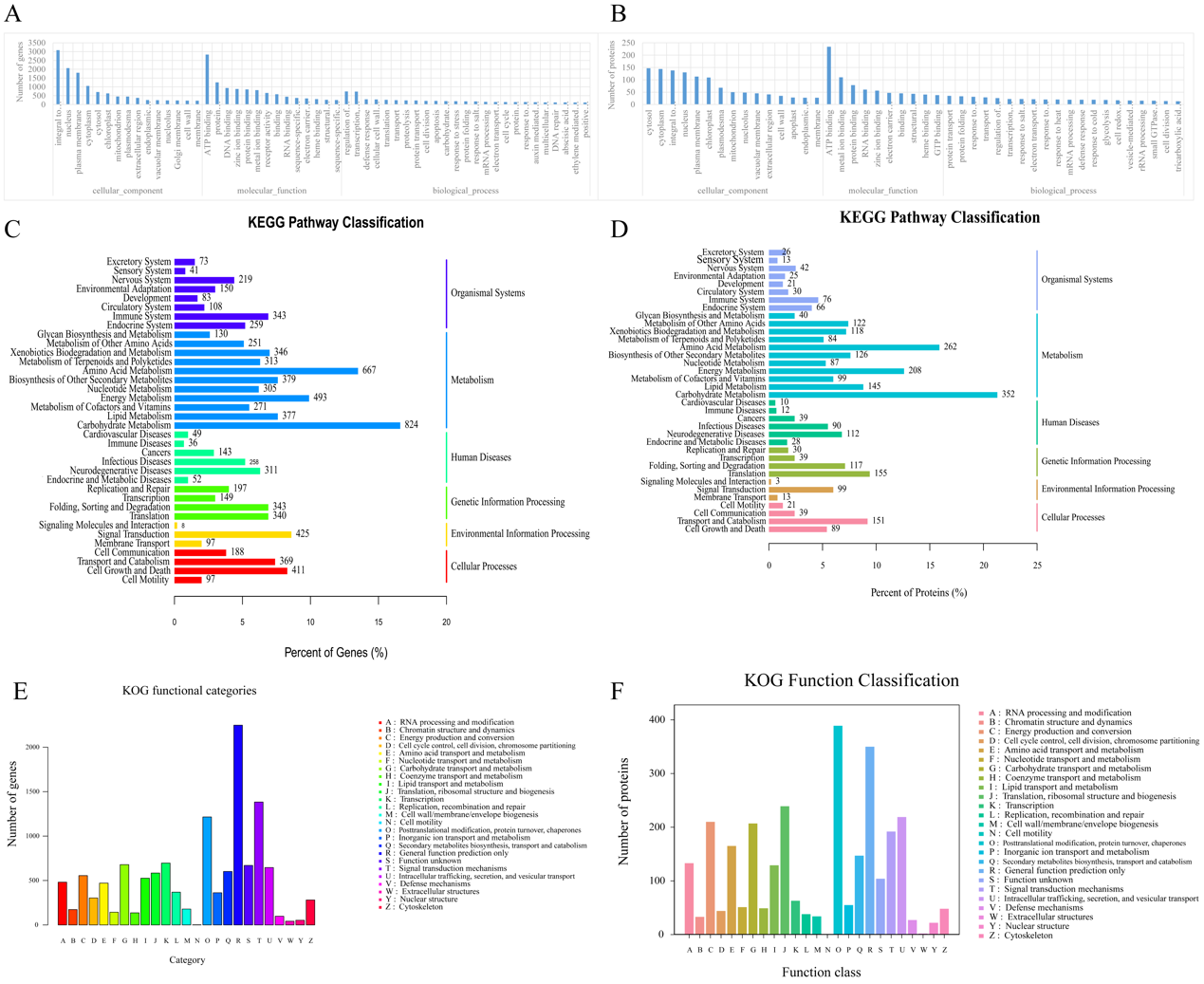
Figure S8. KEGG, GO and KOG function classification

A. Functional annotation of the transcriptome (GO). B. Functional annotation of the proteome (GO). C. Functional annotation of the transcriptome (KEGG). D. Functional annotation of the proteome (KEGG). E. Functional annotation of the transcriptome (KOG). F. Functional annotation of the proteome (KOG).

4 Supplementary Tables

Table S1 Differential expressed transcriptome gene in SM and SC

|  | **Total difference** | **High-expression in Sc** | **High-expression in Sm** |
| --- | --- | --- | --- |
| Transcripts | 27033 | 6897 | 5506 |
| secondary metabolism | 566 | 268 | 298 |
| CYP | 227 | 116 | 111 |
| MYB | 81 | 62 | 19 |
| WRKY | 25 | 12 | 17 |

Table S2 Information of jasmonic acid signal gene at the transcriptional level

| **Gene_ID** | **Name** | **Annotation** | **SC** | **SM** | **log_2_** | ***P*-value** | **FDR** |
| --- | --- | --- | --- | --- | --- | --- | --- |
|  |  |  | **rpkm** | **rpkm** | **fold_change** |  |  |
| *comp6880_c0* | *LOX1.1* | lipoxygenase | 0.48 | 9.62 | -3.89 | 0 | 0 |
| *comp93747_c0* | *Os03g0700700* | lipoxygenase | 0.3 | 4.29 | -3.36 | 0.02 | 0.05 |
| *comp12988_c0* | *LOX5* | lipoxygenase | 1.63 | 22.16 | -3.31 | 0 | 0 |
| *comp22621_c0* | *LOX1.5* | lipoxygenase | 68.46 | 467.06 | -2.39 | 0 | 0 |
| *comp23966_c0* | *LOX5* | lipoxygenase | 38.38 | 150.69 | -1.38 | 0.04 | 0.09 |
| *comp22754_c0* | *LOX1.5* | lipoxygenase | 114.41 | 36.06 | 2.05 | 0 | 0.01 |
| *comp5744_c0* | *LOX1.5* | lipoxygenase | 14.56 | 0.18 | 5.78 | 0 | 0 |
| *comp14182_c1* | *AOC1* | allene oxide cyclase | 9.12 | 48.02 | -2.01 | 0 | 0.01 |
| *comp14182_c0* | *AOC4* | allene oxide cyclase | 14.73 | 85.31 | -1.98 | 0 | 0.01 |
| *comp9693_c0* | *At3g06690* | acyl-CoA oxidase | 0 | 7.86 | -8.28 | 0 | 0 |
| *comp21311_c0* | *ACX2* | acyl-CoA oxidase | 55.53 | 353.5 | -2.14 | 0 | 0.01 |
| *comp21696_c0* | *COI1* | - | 0.54 | 6.8 | -3.2 | 0 | 0.01 |

Table S3 Information of response to abscisic acid gene at the transcriptional level

| **Gene_ID** | **Name** | **Annotation** | **SC_**  **rpkm** | **SM_**  **rpkm** | **log_2_**  **fold_change** |
| --- | --- | --- | --- | --- | --- |
| *comp12017_c0* | *CRK29* | - | 0.04 | 23.26 | -8.63 |
| *comp56732_c0* | *PIP2-1* | aquaporin PIP | 0.00 | 7.34 | -7.46 |
| *comp16810_c0* | *ABCG36* | - | 0.11 | 5.47 | -5.13 |
| *comp45847_c0* | *At1g16060* | AP2-like factor, ANT lineage | 0.32 | 14.39 | -5.08 |
| *comp6466_c0* | *ARIA* | - | 0.59 | 11.12 | -3.82 |
| *comp16810_c2* | *ABCG36* | - | 0.22 | 3.36 | -3.44 |
| *comp78927_c0* | *ELF3* | protein EARLY FLOWERING 3 | 0.32 | 4.60 | -3.42 |
| *comp8578_c1* | *ARIA* | - | 0.73 | 5.30 | -2.46 |
| *comp20577_c0* | *CCA1* | circadian clock associated 1 | 8.41 | 47.11 | -2.33 |
| *comp18139_c0* | *PTR3-A* | proton-dependent oligopeptide transporter, POT family | 12.04 | 92.59 | -2.11 |
| *comp8746_c1* | *RECQL4A* | bloom syndrome protein | 1.15 | 5.65 | -1.90 |
| *comp13682_c0* | *ANN4* | - | 3.56 | 17.33 | -1.77 |
| *comp18497_c0* | *At1g16060* | AP2-like factor, ANT lineage | 4.82 | 21.64 | -1.64 |
| *comp15000_c0* | *PIP2-8* | aquaporin PIP | 163.84 | 628.32 | -1.60 |
| *comp18800_c0* | *PIP2-1* | aquaporin PIP | 132.16 | 476.41 | -1.51 |
| *comp14424_c0* | *RAX2* | myb proto-oncogene protein, plant | 16.89 | 34.31 | -1.33 |
| *comp16967_c0* | *RFS5* | stachyose synthetase | 10.67 | 4.47 | 1.63 |
| *comp16637_c0* | *RH38* | - | 201.93 | 83.46 | 1.66 |
| *comp14904_c0* | *AFP2* | - | 11.03 | 3.62 | 1.98 |
| *comp76114_c0* | *RFS5* | stachyose synthetase | 6.45 | 1.81 | 2.21 |
| *comp12219_c0* | *HVA22E* | - | 6.99 | 1.94 | 2.22 |
| *comp9752_c0* | *HVA22A* | - | 79.71 | 21.53 | 2.25 |
| *comp20195_c0* | *SYP132* | syntaxin 1B/2/3 | 37.22 | 9.66 | 2.28 |
| *comp10760_c0* | *BLH1* | - | 7.34 | 3.13 | 2.33 |
| *comp23083_c0* | *BLH1* | pre-B-cell leukemia transcription factor | 38.51 | 11.32 | 2.34 |
| *comp8895_c0* | *NAC072* | - | 16.76 | 4.22 | 2.36 |
| *comp20255_c1* | *PHT1-4* | MFS transporter, PHS family, inorganic phosphate transporter | 36.90 | 14.59 | 2.47 |
| *comp14933_c0* | *ERF4* | EREBP-like factor | 60.96 | 13.14 | 2.59 |
| *comp2147_c1* | *CRK18* | - | 4.08 | 0.39 | 3.68 |
| *comp54637_c0* | *ABCG25* | ATP-binding cassette, subfamily G （WHITE）, member 2 | 10.08 | 0.77 | 4.07 |
| *comp113402_c0* | *ABCG25* | - | 4.84 | 0.34 | 4.11 |
| *comp66701_c0* | *MYB32* | myb proto-oncogene protein, plant | 7.19 | 0.53 | 4.12 |
| *comp119185_c0* | *CRK18* | DNA (cytosine-5-)-methyltransferase | 3.65 | 0.16 | 4.66 |
| *comp10679_c0* | *COR47* | - | 53.33 | 3.75 | 5.78 |
| *comp8350_c1* | *FBA2* | fructose-bisphosphate aldolase, class I | 102.50 | 0.70 | 7.56 |
| *comp131904_c0* | *UGT74E2* | limonoid glucosyltransferase | 4.28 | 0.00 | 8.15 |
| *comp86000_c0* | *VAL1* | - | 6.68 | 0.00 | 8.56 |
| *comp42947_c0* | *UGT74E2* | N-hydroxythioamide S-beta-glucosyltransferase | 11.36 | 0.00 | 10.25 |
| *comp9642_c0* | *PIMP1* | - | 11.32 | 0.00 | 10.67 |

Table S4 Information of response to gibberellin stimulus gene at the transcriptional level

| **Gene_ID** | **Name** | **Annotation** | **SC_rpkm** | **SM_rpkm** | **log_2_fold_change** |
| --- | --- | --- | --- | --- | --- |
| *comp4698_c0* | *SOC1* | MADS-box transcription factor, plant | 0.1 | 8.03 | -5.86525 |
| *comp39767_c0* | *MYB44* | myb proto-oncogene protein, plant | 0.33 | 23.05 | -5.69018 |
| *comp16381_c0* | *LBD40* | - | 2.6 | 30.49 | -3.62361 |
| *comp118834_c0* | *20ox2* | gibberellin 20-oxidase | 0.34 | 3.78 | -3.05963 |
| *comp20577_c0* | *CCA1* | circadian clock associated 1 | 8.41 | 47.11 | -2.32823 |
| *comp5255_c0* | *MYB44* | myb proto-oncogene protein, plant | 4.87 | 23.04 | -1.8514 |
| *comp14424_c0* | *RAX2* | myb proto-oncogene protein, plant | 16.89 | 34.31 | -1.33273 |
| *comp15691_c0* | *AS1* | myb proto-oncogene protein, plant | 11.19 | 2.96 | 2.30192 |
| *comp11541_c0* | *ATHB-23* | homeobox-leucine zipper protein | 34.22 | 9.17 | 2.327711 |
| *comp24181_c0* | *MYB59* | myb proto-oncogene protein, plant | 67.67 | 17.7 | 2.385602 |
| *comp17468_c0* | *SOC1* | MADS-box transcription factor, plant | 100.73 | 10.38 | 3.638347 |

Table S5 Information of response to fungus gene at the transcriptional level

| **Gene_ID** | **Name** | **Annotation** | **SC_**  **rpkm** | **SM_**  **rpkm** | **log_2_**  **fold_change** |
| --- | --- | --- | --- | --- | --- |
| *comp6560_c0* | *FER* | interleukin-1 receptor-associated kinase 4 | 13.49 | 0.00 | 10.70 |
| *comp6003_c0* | *tlp* | translation initiation factor IF-2 | 104.28 | 0.18 | 9.54 |
| *comp7722_c0* | *PER21* | peroxidase | 4.36 | 0.00 | 7.56 |
| *comp4510_c0* | *PR1B1* | - | 297.10 | 3.32 | 6.85 |
| *comp10679_c0* | *COR47* | - | 53.33 | 3.75 | 5.78 |
| *comp103017_c0* | *WRKY50* | - | 5.33 | 0.11 | 5.74 |
| *comp63159_c0* | *WAKL10* | - | 8.53 | 0.30 | 5.11 |
| *comp12373_c0* | *ERECTA* | - | 61.07 | 2.66 | 4.64 |
| *comp13251_c0* | *BCB* | - | 20.51 | 1.71 | 3.96 |
| *comp10748_c0* | *MLO6* | mlo protein | 6.50 | 0.55 | 3.92 |
| *comp9754_c0* | *FER* | interleukin-1 receptor-associated kinase 4 | 19.53 | 1.88 | 3.75 |
| *comp109784_c0* | *FER* | interleukin-1 receptor-associated kinase 4 | 5.71 | 0.66 | 3.43 |
| *comp10424_c0* | *CYP707A1* | （+）-abscisic acid 8'-hydroxylase | 7.35 | 1.04 | 3.18 |
| *comp11719_c0* | *BAK1* | - | 31.06 | 4.97 | 3.00 |
| *comp18345_c0* | *CYP74A* | hydroperoxide dehydratase | 44.25 | 9.83 | 2.55 |
| *comp10748_c1* | *MLO12* | mlo protein | 6.19 | 1.58 | 2.34 |
| *comp15691_c0* | *AS1* | myb proto-oncogene protein, plant | 11.19 | 2.96 | 2.30 |
| *comp22367_c0* | *FER* | interleukin-1 receptor-associated kinase 4 | 100.24 | 16.32 | 2.23 |
| *comp87585_c0* | *-* | - | 5.62 | 1.65 | 2.14 |
| *comp10816_c0* | *CIPK25* | - | 7.43 | 2.34 | 2.04 |
| *comp11316_c0* | *BIG* | E3 ubiquitin-protein ligase UBR4 | 6.36 | 2.05 | 2.00 |
| *comp100552_c0* | *BIG* | E3 ubiquitin-protein ligase UBR4 | 4.31 | 1.54 | 1.85 |
| *comp14503_c0* | *MLO6* | mlo protein | 3.37 | 11.55 | -1.39 |
| *comp11454_c0* | *MED37E* | heat shock 70kDa protein 1/8 | 67.06 | 231.25 | -1.40 |
| *comp16057_c0* | *WRKY18* | - | 6.40 | 21.04 | -1.59 |
| *comp19997_c0* | *WRKY33* | - | 5.26 | 24.84 | -1.66 |
| *comp9477_c0* | *ERECTA* | - | 1.30 | 5.88 | -1.79 |
| *comp22890_c0* | *FER* | interleukin-1 receptor-associated kinase 4 | 25.59 | 125.03 | -1.91 |
| *comp18468_c1* | *WRKY70* | - | 19.31 | 110.92 | -2.14 |
| *comp16281_c0* | *WRKY70* | - | 17.33 | 103.80 | -2.20 |
| *comp97422_c0* | *WRKY40* | - | 0.61 | 4.32 | -2.44 |
| *comp10670_c0* | *ABR1* | EREBP-like factor | 1.26 | 7.43 | -2.46 |
| *comp7625_c0* | *ERECTA* | - | 0.79 | 5.98 | -2.49 |
| *comp9440_c0* | *-* | - | 38.83 | 311.98 | -2.62 |
| *comp7210_c1* | *ABR1* | EREBP-like factor | 1.71 | 15.71 | -2.82 |
| *comp6809_c0* | *MLO2* | mlo protein | 0.57 | 5.67 | -2.89 |
| *comp12867_c0* | *PER21* | peroxidase | 48.49 | 468.17 | -2.93 |
| *comp21696_c0* | *COI1* | - | 0.54 | 6.80 | -3.20 |
| *comp16810_c2* | *ABCG36* | - | 0.22 | 3.36 | -3.44 |
| *comp20948_c0* | *CYP707A1* | cytochrome P450, family 26, subfamily A | 11.41 | 173.41 | -3.48 |
| *comp5802_c0* | *-* | - | 0.84 | 31.60 | -4.82 |
| *comp16810_c0* | *ABCG36* | - | 0.11 | 5.47 | -5.13 |
| *comp82377_c0* | *WRKY70* | - | 0.00 | 2.92 | -6.17 |

Table S6. MYB transcription factors in SM and SC at the transcriptional level

| **Gene_ID** | **Name** | **log(fold_change)** | **Regulation (SC_VS_SM)** | **Significant** |
| --- | --- | --- | --- | --- |
| *comp12818_c0* | *MYB23* | 2.29 | up | yes |
| *comp14953_c0* | *MYB68* | -1.45 | down | yes |
| *comp49289_c0* | *MYB10* | -4.86 | down | yes |
| *comp69618_c0* | *MYB37* | 4.38 | up | yes |
| *comp82239_c0* | *MYB110* | -3.28 | down | yes |
| *comp990_c0* | *MYB98* | 9.25 | up | yes |
| *comp15691_c0* | *MYB1* | 2.30 | up | yes |
| *comp15783_c0* | telomere repeat-binding factor 4 | -0.40 | down | no |
| *comp16047_c0* | telomere repeat-binding factor 4 | -0.46 | down | no |
| *comp24217_c0* | cell division cycle 5-like protein | 0.20 | up | no |
| *comp17115_c0* | *MYB78* | 1.64 | up | yes |
| *comp130691_c0* | *MYB58* | -2.84 | down | yes |
| *comp7655_c0* | *MYB11* | -4.57 | down | yes |
| *comp11642_c0* | *MYB101* | -0.32 | down | no |
| *comp12028_c0* | *MYB44* | -1.44 | down | yes |
| *comp11142_c0* | *MYB108* | 1.62 | up | yes |
| *comp15250_c0* | *MYB34* | 4.62 | up | yes |
| *comp6132_c1* | *MYB98* | 6.57 | up | yes |
| *comp14785_c0* | *MYB9* | -0.97 | down | no |
| *comp6132_c0* | *MYB98* | 4.38 | up | yes |
| *comp62466_c0* | *MYB81* | 8.44 | up | yes |
| *comp10768_c1* | *MYB61* | 3.55 | up | yes |
| *comp11652_c0* | *MYB39* | -3.13 | down | yes |
| *comp18355_c0* | *MYB39* | -2.08 | down | yes |
| *comp897_c0* | *MYB13* | -6.52 | down | yes |
| *comp15413_c0* | *MYB43* | 0.19 | up | no |
| *comp66701_c0* | *MYB25* | 4.12 | up | yes |
| *comp18355_c1* | *MYB54* | -0.31 | down | no |
| *comp10139_c0* | *MYB28* | -5.80 | down | yes |
| *comp17097_c0* | *MYB22* | 0.83 | up | no |
| *comp5937_c0* | *MYB52* | 1.39 | up | no |
| *comp17097_c1* | *MYB42* | 4.34 | up | yes |
| *comp14858_c0* | transcription factor MYB3R-3-like | 0.59 | up | no |
| *comp2003_c0* | uncharacterized LOC105164125 | -1.29 | down | no |
| *comp20207_c0* | myb-related protein 3R-1 | -0.44 | down | no |
| *comp65795_c0* |  | -0.70 | down | no |
| *comp97110_c0* | transcription factor MYB3R-1 | 0.81 | up | no |
| *comp10920_c0* | *MYB40* | -2.03 | down | yes |
| *comp13377_c0* | *MYB33* | 2.32 | up | yes |
| *comp14665_c0* | *MYB50* | -1.83 | down | yes |
| *comp14793_c0* | *MYB66* | 4.38 | up | yes |
| *comp4372_c0* | *MYB30* | 3.67 | up | yes |
| *comp85154_c0* | *MYB26* | 8.35 | up | yes |
| *comp16280_c0* | *MYB53* | -0.88 | down | no |
| *comp17600_c0* | *MYB32* | -0.90 | down | no |
| *comp19755_c0* | *MYB70* | -1.10 | down | no |
| *comp19955_c0* | *MYB64* | 1.12 | up | no |
| *comp39767_c0* | *MYB5* | -5.69 | down | yes |
| *comp5255_c0* | *MYB32* | -1.85 | down | yes |
| *comp14364_c0* | *MYB14* | -0.71 | down | no |
| *comp11753_c0* | *MYB45* | -4.56 | down | yes |
| *comp11753_c1* | *MYB45* | -2.60 | down | yes |
| *comp11139_c0* | *MYB111* | 2.81 | up | yes |
| *comp11721_c0* | *MYB6* | 1.60 | up | yes |
| *comp6687_c0* | *MYB5-like* | 6.14 | up | yes |
| *comp24181_c0* | *MYB59* | 2.39 | up | yes |
| *comp13673_c0* | *YB48* | -2.65 | down | yes |
| *comp4135_c0* | *MYB101* | 2.05 | up | yes |
| *comp3264_c0* | *MYB17* | 5.66 | up | yes |
| *comp12476_c0* | *MYB20* | -2.77 | down | yes |
| *comp13570_c1* | *MYB56* | 0.67 | up | no |
| *comp22919_c0* | myb-related protein B | 0.54 | up | no |
| *comp22014_c0* | *MYB108* | -0.50 | down | no |
| *comp15413_c1* | *MYB43* | 0.84 | up | no |
| *comp21539_c2* | *MYB91* | -2.62 | down | yes |
| *comp32512_c0* | *MYB75* | -4.90 | down | yes |
| *comp98303_c0* | *MYB111-like* | 4.59 | up | yes |
| *comp24025_c0* | *MYB4* | 12.42 | up | yes |
| *comp16144_c0* | *MYB8* | -1.07 | down | no |
| *comp9151_c0* | *MYB89* | -3.54 | down | yes |
| *comp14424_c0* | *MYB47* | -1.33 | down | yes |
| *comp10768_c0* | *MYB60* | 0.36 | up | no |
| *comp12461_c0* | *MYB27* | -1.14 | down | no |
| *comp14188_c0* | *MYB55* | -0.51 | down | no |
| *comp107512_c0* | *MYB55* | 11.07 | up | yes |
| *comp94183_c0* | *MYB55* | 8.64 | up | yes |
| *comp19396_c0* | repeat-binding factor 1 | -0.01 | down | no |
| *comp17967_c0* | telomere repeat-binding factor 1 | 0.03 | up | no |
| *comp12681_c0* | MYB-like transcription factor ETC3 | 2.25 | up | yes |
| *comp15131_c0* | *MYB114-like* | 0.42 | up | no |

Table S7. bHLH transcription factors in SM and SC at the transcriptional level

| **Gene_ID** | **Name** | **log(fold_change)** | **Regulation (SC_VS_SM)** | **Significant** |
| --- | --- | --- | --- | --- |
| *comp11003_c0* | *bHLH130* | -2.08 | down | yes |
| *comp11060_c0* | *bHLH79* | -2.43 | down | yes |
| *comp11419_c0* | *bHLH25* | -4.02 | down | yes |
| *comp11663_c0* | *bHLH147* | -2.53 | down | yes |
| *comp11761_c0* | *bHLH48* | -1.45 | down | yes |
| *comp12016_c0* | *bHLH126* | -5.53 | down | yes |
| *comp12270_c1* | *bHLH4* | -1.61 | down | yes |
| *comp12294_c0* | *bHLH125* | -2.80 | down | yes |
| *comp12370_c1* | *bHLH13* | -1.47 | down | yes |
| *comp12386_c0* | *bHLH128* | -3.86 | down | yes |
| *comp12727_c0* | *bHLH35* | -0.67 | down | no |
| *comp12761_c1* | *bHLH49* | 1.84 | up | yes |
| *comp130160_c0* | *bHLH25* | -3.11 | down | yes |
| *comp13511_c0* | *bHLH121* | -0.73 | down | no |
| *comp13534_c0* | *bHLH104* | 0.88 | up | no |
| *comp13649_c0* | *bHLH94* | -1.67 | down | yes |
| *comp13765_c0* | *bHLH27* | 0.17 | up | no |
| *comp13826_c0* | *bHLH79* | -2.00 | down | yes |
| *comp13912_c0* | *bHLH135* | -3.24 | down | yes |
| *comp14248_c0* | *bHLH106* | 0.45 | up | no |
| *comp14365_c0* | *bHLH113* | 1.53 | up | yes |
| *comp15028_c0* | *bHLH13* | -2.57 | down | yes |
| *comp15199_c0* | *bHLH149* | -1.11 | down | no |
| *comp15358_c0* | *bHLH68* | 0.95 | up | no |
| *comp15423_c0* | *bHLH133* | -0.39 | down | no |
| *comp15444_c0* | *bHLH63* | -1.23 | down | no |
| *comp15538_c0* | *bHLH130* | -3.55 | down | yes |
| *comp15732_c0* | *bHLH47* | -0.89 | down | no |
| *comp15831_c0* | *bHLH30* | 0.48 | up | no |
| *comp16102_c0* | *bHLH104* | -1.09 | down | no |
| *comp16213_c0* | *bHLH130* | -0.26 | down | no |
| *comp16373_c0* | *bHLH79* | -1.88 | down | yes |
| *comp16456_c0* | *bHLH48* | 0.24 | up | no |
| *comp16740_c1* | *bHLH148* | -0.96 | down | no |
| *comp16968_c0* | *bHLH80* | 0.73 | up | no |
| *comp17111_c0* | *bHLH66* | 0.74 | up | no |
| *comp17140_c0* | *bHLH49* | 0.08 | up | no |
| *comp17401_c0* | *bHLH77* | -1.26 | down | no |
| *comp17401_c1* | *bHLH78* | -0.02 | down | no |
| *comp17406_c0* | *bHLH110* | -0.13 | down | no |
| *comp17749_c0* | *bHLH155* | -1.88 | down | yes |
| *comp18208_c0* | *bHLH93* | -2.86 | down | yes |
| *comp18252_c0* | *bHLH69* | 2.39 | up | yes |
| *comp18295_c0* | *bHLH93* | -0.30 | down | no |
| *comp19526_c0* | *bHLH112* | -2.68 | down | yes |
| *comp19625_c0* | *bHLH78* | -1.83 | down | yes |
| *comp19890_c0* | *bHLH3* | 1.45 | up | yes |
| *comp20315_c0* | *bHLH145* | 0.24 | up | no |
| *comp20389_c0* | *bHLH121* | -0.22 | down | no |
| *comp20821_c0* | *bHLH2* | 0.07 | up | no |
| *comp20835_c0* | *bHLH144* | -0.16 | down | no |
| *comp21364_c0* | *bHLH74* | -0.38 | down | no |
| *comp21839_c0* | *bHLH3* | -0.35 | down | no |
| *comp22443_c0* | *bHLH144* | 0.05 | up | no |
| *comp22602_c0* | *bHLH140* | -0.32 | down | no |
| *comp4791_c0* | *bHLH71* | -4.00 | down | yes |
| *comp5388_c0* | *bHLH55* | -3.07 | down | yes |
| *comp6790_c0* | *bHLH18* | -1.66 | down | no |
| *comp7366_c0* | *bHLH79* | 1.13 | up | no |
| *comp7791_c0* | *bHLH13* | -1.98 | down | yes |
| *comp83938_c0* | *bHLH130* | 2.83 | up | yes |
| *comp8427_c0* | *bHLH93* | -10.52 | down | yes |
| *comp8625_c0* | *bHLH112* | 2.64 | up | yes |
| *comp87292_c0* | *bHLH32* | 5.63 | up | yes |
| *comp8785_c0* | *bHLH120* | 3.10 | up | yes |
| *comp89492_c0* | *bHLH84* | 4.82 | up | yes |
| *comp9139_c0* | *bHLH135* | -5.65 | down | yes |
| *comp9155_c0* | *bHLH13* | -3.46 | down | yes |

Table S8 Differential expressed WRKY gene in SM and SC at the transcriptional level

| **Gene_ID** | **Length** | | **Name** | **ZDS_rpkm** | **ZHDS_rpkm** | **Log2fold_change** | ***P* value** |
| --- | --- | --- | --- | --- | --- | --- | --- |
| *comp10407_c0* | | 1054 | *SmWRKY14* | 26.9 | 0.42 | 6.31 | 0 |
| *comp10228_c0* | | 927 | *SmWRKY61* | 32.23 | 3.9 | 4.12 | 0 |
| *comp14022_c0* | | 1112 | *SmWRKY10* | 11.61 | 0.8 | 4.22 | 0 |
| *comp7261_c1* | | 381 | *SmWRKY37* | 0 | 8.07 | -8.87 | 0 |
| *comp8939_c1* | | 289 | *SmWRKY17* | 10.3 | 0.33 | 5.26 | 0 |
| *comp103017_c0* | | 378 | *SmWRKY52* | 5.33 | 0.11 | 5.74 | 0 |
| *comp22922_c1* | | 1588 | *SmWRKY40* | 4.62 | 40.82 | -3.03 | 0 |
| *comp1012_c0* | | 548 | *SmWRKY41* | 0.28 | 5.01 | -3.81 | 0 |
| *comp7261_c0* | | 220 | *SmWRKY24* | 0 | 6.82 | -7.51 | 0 |
| *comp132630_c0* | | 304 | *SmWRKY30* | 0 | 3.64 | -7.29 | 0 |
| *comp15516_c0* | | 1186 | *SmWRKY57* | 15.61 | 109.79 | -2.43 | 0 |
| *comp16828_c0* | | 1005 | *SmWRKY38* | 16.26 | 4.28 | 2.31 | 0 |
| *comp16281_c0* | | 1050 | *SmWRKY1* | 17.33 | 103.8 | -2.2 | 0 |
| *comp18468_c1* | | 1160 | *SmWRKY70* | 19.31 | 110.92 | -2.14 | 0 |
| *comp8939_c0* | | 326 | *SmWRKY17* | 11.1 | 2.76 | 2.38 | 0 |
| *comp17208_c0* | | 1072 | *SmWRKY50* | 8.4 | 46.01 | -2.1 | 0 |
| *comp11086_c0* | | 1428 | *SmWRKY11* | 2.63 | 12.91 | -1.92 | 0.01 |
| *comp15836_c0* | | 1502 | *SmWRKY30* | 3.94 | 10.59 | -1.83 | 0.01 |
| *comp7205_c0* | | 551 | *SmWRKY25* | 5.34 | 1.78 | 1.97 | 0.01 |
| *comp82377_c0* | | 211 | *SmWRKY60* | 0 | 2.92 | -6.17 | 0.01 |
| *comp19997_c0* | | 1917 | *SmWRKY26* | 5.26 | 24.84 | -1.66 | 0.01 |
| *comp97422_c0* | | 293 | *SmWRKY9* | 0.61 | 4.32 | -2.44 | 0.02 |
| *comp16057_c0* | | 1223 | *SmWRKY58* | 6.4 | 21.04 | -1.59 | 0.02 |
| *comp8640_c0* | | 451 | *SmWRKY24* | 1.46 | 5.98 | -1.65 | 0.04 |
| *comp68816_c0* | | 485 | *SmWRKY33* | 1.33 | 5.3 | -1.6 | 0.05 |

Table S9. Cytochrome P450 gene in SM and SC at the transcriptional level

| **Gene id** | **Name** | **Annotation** | **Log(fold_change)** | **Regulation (SC_VS_SM)** | **Significant** |
| --- | --- | --- | --- | --- | --- |
| *comp1803_c0* | *CYP704C1* | - | 4.94 | up | yes |
| *comp7596_c0* | *CYP704C1* | - | 6.81 | up | yes |
| *comp7596_c1* | *CYP704C1* | - | 8.09 | up | yes |
| *comp97655_c0* | *CYP704C1* | - | -3.71 | down | yes |
| *comp10424_c0* | *CYP707A1* | (+)-abscisic acid 8'-hydroxylase | 3.18 | up | yes |
| *comp20948_c0* | *CYP707A1* | cytochrome P450, family 26, subfamily A | -3.48 | down | yes |
| *comp10424_c1* | *CYP707A3* | cytochrome P450, family 26, subfamily A | 2.06 | up | yes |
| *comp6399_c0* | *CYP707A3* | cytochrome P450, family 26, subfamily A | -2.45 | down | yes |
| *comp79421_c0* | *CYP707A7* | cytochrome P450, family 26, subfamily A | -3.66 | down | yes |
| *comp7056_c0* | *CYP708A2* | - | -7.16 | down | yes |
| *comp16844_c0* | *CYP711A1* | - | 1.51 | up | yes |
| *comp12095_c0* | *CYP716B1* | cytochrome P450, family 26, subfamily A | -8.66 | down | yes |
| *comp15426_c0* | *CYP716B1* | cytochrome P450, family 26, subfamily A | -5.14 | down | yes |
| *comp3621_c0* | *CYP716B1* | cytochrome P450, family 26, subfamily A | -4.81 | down | yes |
| *comp13317_c0* | *CYP716B2* | cytochrome P450, family 26, subfamily A | 1.56 | up | yes |
| *comp20054_c0* | *CYP716B2* | cytochrome P450, family 26, subfamily A | -2.12 | down | yes |
| *comp4025_c0* | *CYP716B2* | cytochrome P450, family 26, subfamily A | -9.94 | down | yes |
| *comp17723_c0* | *CYP71A1* | - | 1.69 | up | yes |
| *comp20040_c0* | *CYP71A1* | - | 1.54 | up | yes |
| *comp32997_c0* | *CYP71A1* | - | -7.16 | down | yes |
| *comp5772_c0* | *CYP71A1* | p-coumarate 3-hydroxylase | 2.97 | up | yes |
| *comp152391_c0* | *CYP71A25* | - | -3.29 | down | yes |
| *comp12598_c1* | *CYP71A6* | - | -8.56 | down | yes |
| *comp13261_c0* | *CYP71A6* | - | -1.63 | down | yes |
| *comp139262_c0* | *CYP71A6* | - | -7.61 | down | yes |
| *comp5754_c0* | *CYP71A6* | - | 4.47 | up | yes |
| *comp13397_c0* | *CYP71A8* | - | -5.63 | down | yes |
| *comp22864_c0* | *CYP71A8* | - | 2.13 | up | yes |
| *comp21749_c0* | *CYP71A9* | cytochrome P450, family 83, subfamily B, polypeptide 1 | -2.18 | down | yes |
| *comp6734_c1* | *CYP71B34* | - | 6.42 | up | yes |
| *comp4019_c0* | *CYP71B37* | - | 5.01 | up | yes |
| *comp11836_c0* | *CYP71B5* | - | -2.23 | down | yes |
| *comp13654_c0* | *CYP71B9* | - | 1.66 | up | yes |
| *comp5414_c0* | *CYP71B9* | - | -4.14 | down | yes |
| *comp12709_c0* | *CYP71D10* | cytochrome P450, family 2, subfamily D | 1.38 | up | yes |
| *comp35508_c0* | *CYP71D10* | - | 5.62 | up | yes |
| *comp5872_c0* | *CYP71D10* | - | 1.98 | up | yes |
| *comp73845_c0* | *CYP71D10* | cytochrome P450, family 2, subfamily D | 9.83 | up | yes |
| *comp10217_c0* | *CYP71D11* | cytochrome P450, family 2, subfamily D | 4.84 | up | yes |
| *comp109579_c0* | *CYP71D12* | - | 7.64 | up | yes |
| *comp82556_c0* | *CYP71D15* | - | 4.42 | up | yes |
| *comp19121_c0* | *CYP71D55* | cytochrome P450, family 2, subfamily D | 2.03 | up | yes |
| *comp19833_c0* | *CYP71D55* | cytochrome P450, family 2, subfamily D | -3.32 | down | yes |
| *comp42470_c0* | *CYP71D55* | cytochrome P450, family 2, subfamily D | 5.55 | up | yes |
| *comp48_c1* | *CYP71D55* | cytochrome P450, family 2, subfamily D | -8.25 | down | yes |
| *comp5710_c0* | *CYP71D55* | cytochrome P450, family 2, subfamily D | -5.49 | down | yes |
| *comp10161_c0* | *CYP71D9* | - | 1.87 | up | yes |
| *comp12694_c0* | *CYP71D9* | - | 1.75 | up | yes |
| *comp4922_c0* | *CYP71D9* | - | -3.31 | down | yes |
| *comp22075_c0* | *CYP71D95* | cytochrome P450, family 2, subfamily D | -1.52 | down | yes |
| *comp2875_c0* | *CYP71D95* | - | -8.31 | down | yes |
| *comp32553_c0* | *CYP71D95* | cytochrome P450, family 2, subfamily D | -2.26 | down | yes |
| *comp8620_c0* | *CYP71D95* | cytochrome P450, family 2, subfamily D | 6.12 | up | yes |
| *comp8620_c1* | *CYP71D95* | ferulate-5-hydroxylase | 3.96 | up | yes |
| *comp48_c0* | *CYP71Z6* | cytochrome P450, family 2, subfamily D | -8.02 | down | yes |
| *comp1960_c0* | *CYP720B1* | cytochrome P450, family 90, subfamily B, polypeptide 1 (steroid 22-alpha-hydroxylase) | -1.70 | down | yes |
| *comp10023_c0* | *CYP72A1* | - | 7.05 | up | yes |
| *comp12441_c0* | *CYP72A1* | - | -7.41 | down | yes |
| *comp15021_c0* | *CYP72A1* | - | 3.02 | up | yes |
| *comp18825_c0* | *CYP72A1* | unspecific monooxygenase | 1.59 | up | yes |
| *comp19986_c0* | *CYP72A1* | - | -2.24 | down | yes |
| *comp20001_c0* | *CYP72A1* | - | 1.59 | up | yes |
| *comp20990_c0* | *CYP72A1* | - | 1.58 | up | yes |
| *comp22141_c0* | *CYP72A1* | unspecific monooxygenase | -2.28 | down | yes |
| *comp23603_c0* | *CYP72A1* | unspecific monooxygenase | 4.05 | up | yes |
| *comp5257_c2* | *CYP72A1* | - | 8.75 | up | yes |
| *comp8747_c0* | *CYP72A1* | cytochrome P450, family 3, subfamily A | -4.93 | down | yes |
| *comp92552_c0* | *CYP72A1* | - | 4.81 | up | yes |
| *comp9644_c0* | *CYP72A1* | - | -5.69 | down | yes |
| *comp5257_c0* | *CYP72C1* | - | 6.91 | up | yes |
| *comp64534_c0* | *CYP72C1* | cytochrome P450, family 4, subfamily F (leukotriene-B4 20-monooxygenase) | -10.14 | down | yes |
| *comp19887_c0* | *CYP734A1* | cytokinin trans-hydroxylase | 1.35 | up | yes |
| *comp3135_c0* | *CYP734A1* | cytokinin trans-hydroxylase | -3.85 | down | yes |
| *comp8815_c0* | *CYP734A1* | cytochrome P450, family 4, subfamily F (leukotriene-B4 20-monooxygenase) | 2.29 | up | yes |
| *comp16562_c0* | *CYP735A2* | cytokinin trans-hydroxylase | 3.14 | up | yes |
| *comp6780_c0* | *CYP735A2* | cytokinin trans-hydroxylase | -5.07 | down | yes |
| *comp8252_c0* | *CYP735A2* | cytokinin trans-hydroxylase | -5.04 | down | yes |
| *comp66261_c0* | *CYP73A11* | trans-cinnamate 4-monooxygenase | 9.88 | up | yes |
| *comp103900_c0* | *CYP73A14* | trans-cinnamate 4-monooxygenase | 3.68 | up | yes |
| *comp18345_c0* | *CYP74A* | hydroperoxide dehydratase | 2.55 | up | yes |
| *comp2301_c0* | *CYP74A* | hydroperoxide dehydratase | -3.51 | down | yes |
| *comp5877_c0* | *CYP74A* | hydroperoxide dehydratase | 3.50 | up | yes |
| *comp14705_c0* | *CYP74A2* | hydroperoxide lyase | -2.42 | down | yes |
| *comp100126_c0* | *CYP75B1* | flavonoid 3'-monooxygenase | -7.23 | down | yes |
| *comp82215_c0* | *CYP75B1* | ferulate-5-hydroxylase | -2.27 | down | yes |
| *comp20463_c1* | *CYP75B2* | - | 3.01 | up | yes |
| *comp50773_c0* | *CYP75B2* | p-coumarate 3-hydroxylase | -8.65 | down | yes |
| *comp90756_c0* | *CYP75B2* | flavonoid 3'-monooxygenase | -7.99 | down | yes |
| *comp18456_c0* | *CYP76A2* | - | 3.34 | up | yes |
| *comp18456_c1* | *CYP76A2* | - | -4.37 | down | yes |
| *comp7591_c0* | *CYP76A2* | - | -4.95 | down | yes |
| *comp11871_c0* | *CYP76B10* | - | 3.65 | up | yes |
| *comp13540_c0* | *CYP76B10* | - | 1.43 | up | yes |
| *comp16677_c1* | *CYP76B10* | - | 3.04 | up | yes |
| *comp224_c1* | *CYP76B6* | p-coumarate 3-hydroxylase | -8.51 | down | yes |
| *comp22490_c1* | *CYP76B6* | - | 6.00 | up | yes |
| *comp22692_c0* | *CYP76B6* | - | 1.99 | up | yes |
| *comp6734_c0* | *CYP76B6* | - | 6.77 | up | yes |
| *comp69833_c0* | *CYP76B6* | - | 4.29 | up | yes |
| *comp8706_c0* | *CYP76C1* | - | 4.26 | up | yes |
| *comp16677_c0* | *CYP76C2* | - | 2.64 | up | yes |
| *comp3164_c0* | *CYP76C2* | - | 5.00 | up | yes |
| *comp81852_c0* | *CYP76C2* | - | -2.54 | down | yes |
| *comp113623_c0* | *CYP77A2* | - | -4.49 | down | yes |
| *comp21733_c0* | *CYP78A3* | - | 2.04 | up | yes |
| *comp1018_c0* | *CYP81D1* | - | -2.70 | down | yes |
| *comp14195_c0* | *CYP81D1* | - | 4.21 | up | yes |
| *comp14195_c1* | *CYP81D1* | - | 4.60 | up | yes |
| *comp14528_c0* | *CYP81D1* | - | 2.28 | up | yes |
| *comp20499_c0* | *CYP81D1* | - | -1.42 | down | yes |
| *comp8246_c0* | *CYP81D1* | - | 2.87 | up | yes |
| *comp17645_c0* | *CYP81E1* | - | 3.05 | up | yes |
| *comp19089_c0* | *CYP81F1* | - | 1.81 | up | yes |
| *comp73410_c0* | *CYP81F1* | - | 2.83 | up | yes |
| *comp3181_c0* | *CYP82A3* | - | -5.45 | down | yes |
| *comp11694_c0* | *CYP82C2* | - | 3.37 | up | yes |
| *comp9651_c0* | *CYP82C2* | - | 6.42 | up | yes |
| *comp17261_c0* | *CYP82G1* | - | 4.44 | up | yes |
| *comp24041_c0* | *CYP83B1* | cytochrome P450, family 83, subfamily B, polypeptide 1 | 2.39 | up | yes |
| *comp9370_c0* | *CYP83B1* | cytochrome P450, family 83, subfamily B, polypeptide 1 | 5.33 | up | yes |
| *comp9599_c1* | *CYP83B1* | cytochrome P450, family 83, subfamily B, polypeptide 1 | -2.15 | down | yes |
| *comp116035_c0* | *CYP86A2* | - | 2.35 | up | yes |
| *comp137873_c0* | *CYP86A2* | - | 2.25 | up | yes |
| *comp20108_c0* | *CYP86A2* | - | -5.27 | down | yes |
| *comp71036_c0* | *CYP86A2* | - | -3.81 | down | yes |
| *comp71596_c0* | *CYP86A2* | - | 3.20 | up | yes |
| *comp9201_c0* | *CYP86B1* | - | -3.71 | down | yes |
| *comp1298_c0* | *CYP89A2* | - | -5.70 | down | yes |
| *comp17646_c0* | *CYP89A2* | - | 1.87 | up | yes |
| *comp11850_c0* | *CYP90D2* | cytochrome P450, family 26, subfamily A | -6.75 | down | yes |
| *comp19454_c0* | *CYP93B1* | cytochrome P450, family 1, subfamily A, polypeptide 1 | -2.26 | down | yes |
| *comp12289_c0* | *CYP94A1* | - | 2.40 | up | yes |
| *comp20108_c2* | *CYP94A1* | - | 1.75 | up | yes |
| *comp20845_c0* | *CYP94A1* | - | 1.96 | up | yes |
| *comp21872_c0* | *CYP94A1* | - | 2.26 | 2.26 | yes |
| *comp92967_c0* | *CYP94A1* | - | 3.00 | up | yes |
| *comp16193_c0* | *CYP94A2* | - | 2.06 | up | yes |
| *comp19768_c0* | *CYP97C1* | carotenoid epsilon-ring hydroxylase | -2.58 | down | yes |
| *comp20524_c0* | *CYP98A2* | p-coumarate 3-hydroxylase | -1.32 | down | yes |

Table S10 Differential expressed proteomic protein in SM and SC

|  | **Total difference** | **High-expression in Sc** | **High-expression in Sm** |
| --- | --- | --- | --- |
| Transcripts | 3586 | 348 | 469 |
| Secondary metabolism | 133 | 62 | 71 |
| Related to terpenoids synthesis | 13 | 3 | 10 |
| Related to of phenolic acids pathway | 19 | 8 | 11 |

Table S11 Information of jasmonic acid signal protein

| **Gene id** | **KEGG KO Description** | **EC** | **quant_ratio**  **(SC_VS_SM)** | **Regulation**  **(SC_VS_SM)** | **Significance**  **(SC_VS_SM)** |
| --- | --- | --- | --- | --- | --- |
| *comp22754_c0* | lipoxygenase | EC:1.13.11.12 | 1.644 | up | yes |
| *comp22621_c0* | lipoxygenase | EC:1.13.11.12 | 0.545 | down | yes |
| *comp22240_c0* | lipoxygenase | EC:1.13.11.12 | 0.67 | down | yes |
| *comp14182_c0* | allene oxide cyclase | EC:5.3.99.6 | 1.014 | up | no |
| *comp14852_c0* | allene oxide cyclase | EC:5.3.99.6 | 1.044 | up | no |

Table S12 Information of response to abscisic acid protein

| **Gene id** | **Name** | **Quant_ratio**  **(SC_VS_SM)** | **Significance**  **(SC_VS_SM)** | **Regulation**  **(SC_VS_SM)** |
| --- | --- | --- | --- | --- |
| *comp16637_c0* | *RH38* | 1.675 | yes | up |
| *comp24084_c0* | *VLN4* | 1.2 | yes | up |
| *comp10679_c0* | *COR47* | 4.27 | yes | up |
| *comp19299_c0* | *RBG8* | 0.77 | yes | down |

Table S13 Information of response to fungus protein

| **Gene id** | **Name** | **Quant_ratio**  **（SC_VS_SM）** | **Significance**  **（SC_VS_SM）** | **Regulation**  **（SC_VS_SM）** |
| --- | --- | --- | --- | --- |
| *comp4510_c0* | *PR1B1* | 3.80 | yes | up |
| *comp20742_c0* | *ACX1* | 1.23 | yes | up |
| *comp10679_c0* | *COR47* | 4.27 | yes | up |
| *comp21696_c1* | *COI1* | 0.65 | yes | down |

Table S14 Information of CYP450 protein

| **Gene id** | **Name** | **Quant_ratio (SC_VS_SM)** | **Regulation (SC_VS_SM)** |
| --- | --- | --- | --- |
| *comp9651_c0* | *CYP82D70* | 2.262 | up |
| *comp24041_c0* | *CYP71AT146* | 1.712 | up |
| *comp19454_c0* | *CYP93B25* | 1.514 | up |
| *comp23603_c0* | *CYP749A39* | 1.799 | up |
| *comp17261_c0* | *CYP82U4* | 2.29 | up |
| *comp19463_c0* | *CYP76AK1* | 0.705 | down |
| *comp21835_c0* | *CYP92A73* | 0.765 | down |
| *comp19833_c0* | *CYP71BE37* | 0.72 | down |
| *comp21728_c0* | *CYP71D375* | 0.697 | down |
| *comp20054_c0* | *CYP716C12* | 0.728 | down |
| *comp20590_c0* | *CYP706C35* | 0.798 | down |

Table S15. Information of 2-oxoglutarate-dependent dioxygenase and protein

| **Gene id** | **Name** | **ZDS_rpkm** | **ZHDS_rpkm** | **Quant_ratio(ZDS/ZHDS)** | **Significance** |
| --- | --- | --- | --- | --- | --- |
| *comp22500_c0* | - | 172.84 | 161.15 | 2.011 | no |
| *comp16320_c0* | - | 18.05 | 28.18 | 0.505 | no |
| *comp17878_c0* | naringenin 3-dioxygenase | 22.71 | 15.38 | - | - |
| *comp19656_c0* | flavonol synthase | 9.7 | 75.28 | - | - |
| *comp17762_c0* | - | 10.46 | 26.34 | - | - |
| *comp13878_c0* | aminocyclopropanecarboxylate oxidase | 85.1 | 5.45 | 1.146 | no |
| *comp16817_c0* | aminocyclopropanecarboxylate oxidase | 9.94 | 4.39 | - | - |
| *comp14285_c0* | gibberellin 3-beta-dioxygenase | 11.85 | 11.31 | - | - |
| *comp12363_c0* | aminocyclopropanecarboxylate oxidase | 6.57 | 2.98 | - | - |
| *comp21303_c0* | aminocyclopropanecarboxylate oxidase | 33.12 | 19.5 | - | - |
| *comp19257_c0* | flavonol synthase | 48.43 | 14.14 | 1.145 | no |
| *comp7600_c0* | flavonol synthase | 20.74 | 0.36 | - | - |
| *comp9786_c0* | aminocyclopropanecarboxylate oxidase | 2.03 | 3.5 | - | - |
| *comp15499_c0* | flavonol synthase | 14.62 | 5.52 | - | - |
| *comp4804_c0* | aminocyclopropanecarboxylate oxidase | 12.8 | 0.55 | - | - |
| *comp56797_c0* | naringenin 3-dioxygenase | 12.12 | 0 | - | - |
| *comp20015_c1* | aminocyclopropanecarboxylate oxidase | 5.18 | 10.57 | - | - |
| *comp15553_c0* | flavonol synthase | 258.53 | 73.76 | 2.308 | yes |
| *comp16573_c0* | aminocyclopropanecarboxylate oxidase | 201.29 | 43.63 | 1.661 | no |
| *comp20597_c0* | aminocyclopropanecarboxylate oxidase | 53.63 | 62.38 | 0.847 | no |
| *comp22694_c0* | flavonol synthase | 48.42 | 72.31 | 1.018 | no |
| *comp18661_c0* | aminocyclopropanecarboxylate oxidase | 89.84 | 56.57 | 0.686 | no |
| *comp19779_c0* | Amino-cyclopropanecarboxylate oxidase | 68.68 | 32.68 | 0.614 | yes |
| *comp17451_c0* | flavonol synthase | 25.76 | 29.16 | - | - |
| *comp8659_c0* | gibberellin 2-oxidase | 0.48 | 12.86 | - | - |
| *comp23854_c1* | aminocyclopropanecarboxylate oxidase | 8.89 | 55.2 | - | - |
| *comp22239_c0* | - | 17.91 | 58.89 | - | - |
| *comp21472_c0* | naringenin 3-dioxygenase | 218.4 | 196.18 | 1.874 | yes |
| *comp14481_c0* | Amino-cyclopropanecarboxylate oxidase | 1.46 | 20.93 | 0.431 | yes |
| *comp19664_c0* | Amino-cyclopropanecarboxylate oxidase | 144.12 | 190.91 | - | - |
| *comp13901_c0* | - | 17.07 | 2.66 | - | - |
| *comp13323_c0* | Amino-cyclopropanecarboxylate oxidase | 16.29 | 5.18 | 0.838 | no |
| *comp19310_c0* | - | 30.88 | 42.91 | - | - |
| *comp134841_c0* | gibberellin 20-oxidase | 0 | 3.05 | - | - |
| *comp23360_c0* | Amino-cyclopropanecarboxylate oxidase | 31.09 | 75.08 | - | - |
| *comp13291_c1* | Amino-cyclopropanecarboxylate oxidase | 3.33 | 13.62 | 0.556 | yes |
| *comp3314_c0* | - | 17.22 | 0 | - | - |
| *comp15572_c0* | - | 5.86 | 49.68 | 0.679 | no |
| *comp16376_c0* | - | 48.44 | 61.22 | - | - |
| *comp7867_c0* | - | 1.59 | 202.02 | - | - |
| *comp14509_c0* | - | 608.07 | 130.27 | 2.999 | no |
| *comp22425_c0* | - | 217.3 | 272.48 | 1.098 | no |
| *comp18540_c0* | - | 42.6 | 81.64 | 0.796 | no |
| *comp15589_c0* | naringenin 3-dioxygenase | 21.78 | 115.2 | 0.207 | no |
| *comp11659_c0* | - | 0.24 | 22.27 | - | - |
| *comp19451_c0* | - | 105.9 | 276.26 | - | - |
| *comp12155_c0* | aminocyclopropanecarboxylate oxidase | 5.21 | 3.13 | - | - |
| *comp13451_c0* | - | 4.63 | 59.99 | - | - |
| *comp20939_c0* | prolyl 4-hydroxylase | 90.97 | 155.02 | 0.953 | no |
| *comp17499_c0* | - | 39.18 | 122.77 | - | - |
| *comp17462_c0* | gibberellin 2-oxidase | 31.93 | 50.72 | - | - |
| *comp17080_c0* | prolyl 4-hydroxylase | 68.73 | 33 | - | - |
| *comp7386_c0* | - | 2.9 | 4.51 | - | - |
| *comp14357_c1* | prolyl 4-hydroxylase | 91.38 | 22.5 | - | - |
| *comp14000_c0* | - | 3.54 | 18.67 | - | - |
| *comp16069_c0* | - | 5.23 | 4.61 | - | - |
| *comp7284_c0* | gibberellin 2-oxidase | 0.31 | 6.42 | - | - |
| *comp110078_c0* | gibberellin 3-beta-dioxygenase | 0 | 4.58 | - | - |

Table S16 Information of tanshinone pathway related gene

| **Name** | **Gene id** | **log2FC(ZDS/ZHDS) of Transcriptome** | **Quant_ratio of proteome (ZDS/ZHDS)** | **Significance (ZDS/ZHDS)** |
| --- | --- | --- | --- | --- |
| *CYP71D373* | *comp7847_c0* | -1.481076 | - | - |
| *CYP71D375* | *comp21728_c0* | -0.667213 | 0.697 | yes |
| *CYP71D464* | *comp12694_c0* | 1.277715 | - | - |
| *CYP76AH1* | *comp22692_c0* | 1.5858528 | 0.951 | no |
| *CYP76AK1* | *comp19463_c0* | -0.0604645 | 0.705 | yes |
| *SmAACT1* | *comp18854_c0* | 0.3949632 | 1.005 | no |
| *SmAACT2* | *comp18100_c0* | 0.3034651 | 1.226 | no |
| *SmAACT3* | *comp19861_c0* | -0.8762881 | 0.887 | no |
| *SmAACT4* | *comp17753_c0* | -0.1518034 | 1.181 | no |
| *SmAACT5* | *comp19861_c0* | -0.8762881 | 0.887 | no |
| *SmCMK* | *comp19689_c0* | 0.3890879 | - | - |
| *SmCPS1* | *comp22134_c0* | 1.4233921 | 1.53 | yes |
| *SmCPS3* | *comp162022_c0* | -1.819668 | - | - |
| *SmCPS5* | *comp8913_c1* | -1.8555319 | - | - |
| *SmDXS1* | *comp19857_c0* | -0.2388466 | - | - |
| *SmDXS2* | *comp23923_c0* | 2.788821 | - | - |
| *SmDXS3* | *comp21948_c0* | -1.261492 | 1.031 | no |
| *SmDXS4* | *comp21176_c0* | 0.3073018 | - | - |
| *SmDXS5* | *comp19857_c0* | -0.2388466 | - | - |
| *SmDXS6* | *comp21948_c0* | -1.261492 | 1.031 | no |
| *SmDXS7* | *comp19857_c0* | -0.2388466 | - | - |
| *SmGPPS1* | *comp19276_c0* | -0.1874692 | 1.061 | no |
| *SmGPPS2* | *comp12710_c1* | -1.9541962 | - | - |
| *SmGPPS4* | *comp66299_c0* | -2.2383479 | - | - |
| *SmGPPS.LSU1* | *comp8253_c0* | -3.809414 | - | - |
| *SmSmGPPS.SSUI.1* | *comp18517_c0* | 0.9379447 | - | - |
| *SmSmGPPS.SSUI.2* | *comp14521_c0* | -0.3616825 | - | - |
| *SmSmGPPS.SSUII.1* | *comp13237_c0* | 0.6388673 | - | - |
| *SmSmGPPS.SSUII.2* | *comp18156_c0* | -1.2017254 | 0.628 | no |
| *SmHDR1* | *comp19443_c0* | 0.962711 | 1.259 | yes |
| *SmHDS* | *comp21514_c0* | 0.847579 | 0.986 | no |
| *SmHMGR1* | *comp20754_c0* | -1.1563376 | - | - |
| *SmHMGR2* | *comp21764_c0* | -0.8418127 | - | - |
| *SmHMGR5* | *comp24278_c0* | 0.9021696 | - | - |
| *SmHMGS1* | *comp17582_c0* | 0.6800412 | 0.887 | no |
| *SmIPI1* | *comp18879_c0* | -0.7203009 | 0.874 | no |
| *SmIPI2* | *comp10302_c0* | 0.1536229 | - | - |
| *SmKSL1* | *comp21823_c0* | -1.2462589 | 0.888 | no |
| *SmKSL2* | *comp24059_c0* | 1.4966838 | - | - |
| *SmKSL5* | *comp4967_c0* | -2.792855 | - | - |
| *SmMCT* | *comp15960_c0* | 0.0122394 | 1.029 | no |
| *SmMDC1* | *comp19181_c0* | -0.215345 | 0.812 | no |
| *SmMDS* | *comp13642_c0* | -0.586868 | - | - |
| *SmMK* | *comp16422_c0* | -1.5949913 | 0.739 | yes |
| *SmPMK* | *comp21112_c0* | -1.2577809 | 0.548 | yes |

Table S17 Information of rosmarinic acid pathway related gene

| **Gene id** | **Name** | **log2FC(ZDS/ZHDS)** | **Quant_ratio (ZDS/ZHDS)** | **Significance (ZDS/ZHDS)** |
| --- | --- | --- | --- | --- |
| *comp19606_c0* | *4CL-like1* | 0.54358 | 1.765 | no |
| *comp19461_c1* | *4CL-like2* | 4.261212 | 1.048 | no |
| *comp8957_c0* | *4CL-like3* | -3.74739 | - | - |
| *comp756_c0* | *4CL-like3* | -2.91265 | - | - |
| *comp20712_c0* | *4CL-like4* | -1.28422 | 0.642 | yes |
| *comp12353_c0* | *4CL-like5* | 0.983007 | - | - |
| *comp22731_c0* | *4CL-like7* | -1.35364 | - | - |
| *comp54506_c0* | *4CL1* | 2.808365 | - | - |
| *comp20858_c0* | *4CL2* | -0.97243 | 0.966 | no |
| *comp23028_c0* | *4CL3* | 0.522261 | 1.121 | no |
| *comp21166_c0* | *C4H1* | -1.06088 | 0.803 | yes |
| *comp66261_c0* | *C4H1* | 3.642702 | - | - |
| *comp20700_c0* | *CYP98A14* | -0.33147 | 0.895 | no |
| *comp13941_c1* | *HCT1* | 2.512097 | - | - |
| *comp14716_c0* | *HCT2* | 0.855169 | - | - |
| *comp56084_c0* | *HCT3* | 3.343271 | - | - |
| *comp10820_c0* | *HPPR3* | -1.56331 | - | - |
| *comp16323_c0* | *HPPR1* | 0.05173 | 1.153 | no |
| *comp22753_c0* | *PAL1* | -1.08963 | 0.922 | no |
| *comp22753_c0* | *PAL2* | -1.08963 | 0.922 | no |
| *comp20996_c0* | *RAS1* | -1.10307 | 1.108 | no |
| *comp20996_c0* | *SmRAS1* | -1.10307 | 1.108 | no |
| *comp14716_c0* | *SmRAS2* | 0.855169 | - | - |
| *comp13941_c1* | *SmRAS3* | 2.512097 | - | - |
| *comp56084_c0* | *SmRAS4* | 3.343271 | - | - |
| *comp17241_c0* | *TAT1* | -0.09332 | - | - |
| *comp12075_c0* | *TAT2* | -3.07099 | - | - |
| *comp12768_c0* | *SbLAC4* | 2.02415 | - | - |

Table S18 79 full-length *SmWRKY* genes and WRKY gene in *Arabidopsis thaliana*

| 79 full-length *SmWRKY* genes and WRKY gene in *Arabidopsis thaliana* |
| --- |
| >*SmWRKY11*  MAVDLLGYRNLNRHAPSVQEAAPAGFKSMQQLILAASHQNQPSNFTVFSSQKVSSNVNRTGHARFRRALVQPELALAPVQYSEPRDSGSHQLQTLNLFSDSPASAAAAAPARALDLSKEMIGDDVLSLSATVSTSGNSSATFASTITGEGSVSNGKSGALSTVPISAGKPPLSGKRCREHDNISGKISDSSRCHCKKRKYRVKRTIRVPAVSSKVSDIPADEYSWRKYGQKPIKGSPHPRGYYKCSTVKGCPARKHVERARDDPTMVIVTYEGEHRHAKQEIPAAVENAKATTSL |
| >*SmWRKY12* |
| MEGERGLAPIYDRHLQISFSAPPHHQDQEMGFVQFEDHNPVLSFLVAPPQIAAKATTATNNNGSLGFNHAELAVNRPSWNNDQVEVVDPKVVNDENCSGNANDGGNSWWRSCSSDKNKVKIRRKLREPRFCFQTRSDVDVLDDGYKWRKYGQKVVKNSLHPRSYYRCTHNNCRVKKRVERLSEDCRMVITTYEGRHNHSPSDDSNSSDCYNSF |
| >*SmWRKY12-like1* |
| MEGERGLAPIYDHHLQISFSAPPHHQEQEMGFVQFEDHNPVLSFLVAPPQMAAKATTATNNNCSLGFNHAELAVNRPSWNNDQVEVVDPKVVNDENCSGNANDGGNSWWRSCSSDKNKVKIRRKLREPRFCFQTRSDVDVLDDGYKWRKYGQKVVKNSLHPRYIITNKMFQTLHQL |
| >*SmWRKY12-like2* |
| MEGERGLAPIYDHHLQISFSAPPHHQEQEMGFVQFEDHNPVLSFLVAPPQMAAKATTATNNNCSLGFNHAELAVNRPSWNNDQVEVVDPKVVNDENCSGNANDGGNSWWRSCSSDKNKVKIRRKLREPRFCFQTRSDVDVLDDGYKWRKYGQKVVKNSLHPRSYYRCTHNNCRVKKRVERLSEDCRMVITTYEGRHNHSPSDDSNSSDCYNSF |
| >*SmWRKY13* |
| MSSQAMLNQSLFEEQEMIISSQLGIFPFHTNLSSSLPLLGFNQPLKTLSADSPLITLSDHSTLTKQKEDFTPHFSGLPHLLSLQRSAASNYWAWGELSDCMSMKRSGGEDHLGVSAMKMKKIKARRKVREPRFCFKTMSDVDVLDDGYKWRKYGQKVVKNTQHPRSYYRCTQDNCRVKKRVERLADDPRMVITTYEGRHIHSPSHDEDDSQASAQLTNFFW |
| >*SmWRKY14* |
| MCSHLLKMMENYQGDLADIVRATTSAAAIDGGSTPSQQSAAPDWQFSRNSINYPPEYFGDPFSYIRDPLTPNMDTPPPVSELLYNSSIVNGTGVINPDEMKRPSNIFSRMLQISPNAKLQPCDPQAAAPPQRGLKNPPILVSNNGGLISPTTSALQISSPRNTGIKRRKSQAKKVVCIPAPAPANSRATGEVVPSDLWAWRKYGQKPIKGSPYPRGYYRCSSSKGCSARKQVERSRTDPNMLVITYTSEHNHPWPTQRNALAGSTRSQAAKNSKDSSQKPTNQSSSAAVKLEELPEFELPDSSSNHQHDSFFADLAEIGDDPLNLFGPHDNNDPFSFYDWPTTTTTADDPARNT |
| >*SmWRKY15* |
| MAVELMRGYNFTAKMEENAAQEAAAAGLQSVEKLIRLLSHSHQQNQQEPSSSAADYQAVADVAVDKFKKFISLLDRSRTGHARFRRGPIPNPPQPRPQPKVEPVTEPVGPPPQPPKTEEKEPGSKIYHPTPIQRLPPLPLPHHHHQLIRNGSLERKESTTTINFSSPANSFLSSVTGDTDSLQPSMSSGFQITNLSQVSSGGRPMASTSSFKRKCSSLDDSSAKCGGASSGSRCHCPKKRKSRLKKVVRVPAISMKMADIPPDDYSWRKYGQKPIKGSPHPRGYYKCSSLRGCPARKHVERALDDPAMLIVTYEGDHNHAHSNAEALVLESS |
| >*SmWRKY2* |
| MPMGGFDDHVAIMGEWMPPSPNPGAFFLSMMNDDVGARLAVESDSGNRRGPEQQGTSRNGDQKDQSEASFGDDETNNCKAAGEPKLSSRGGLMERIAARAGFNAPRLNTENIRPSDLSKKPEVRSPYLTIPPGLSPTTLLDSPVFLSNSLVLPSPTTGKFSFASSGDYRDSTLMIGDSDKGKENNFEDINASSFAFKPITESASFPYLNATNKTFPSAGFTVHSNTSLQRQSIEPTKSHLANNSTLFQEASFSKSDLGNNGSSNLLQQTHSSGAMAHSPPFDEQNDDGDQRSSGDPNSGGSTADDGYNWRKYGQKHVKGSEYPRSYYKCTHPSCPVKKKVERSQEGHITEIIYKGAHNHAKPPPNRRSTLGSSNALGDMQLDAEEPGTGDPIWSAVQNGNAAGGWKPDNLETTPSALPGQEYNNGGTTAPQAQNGTTYESVDGVDGCSTFSNEEEEDDRATRGSMSLGYDGEGDESESKRRKLETYAPEMGGASRPIREPRVVVQTTSEVDILDDGYRWRKYGQKVVKGNPNPRSYYKCTSAGCNVRKHVERASHDLKSVITTYEGKHNHDVPAARNSGAHVNSGGQNTHPPQQAHAASGNVHRPEPSHLRGSTAWFERPALGSFGLGPGHGFGYRMDQAGLGMGPNQGKLGVHPYLGQQNRAVKEMCYMLPKGEPKVEPVTDAGGLNSSNASSVYEQLMSRLPHMGNINIGREYSESLCELLGLT |
| >*SmWRKY20* |
| MGAVSSKRIASETGELERRASTNLPLATSNSNIEAAFPMTSEKIMSGMPHTKSHPNVKQEKDSGVVPEKEPSHLSQKQNSDTVNHTSFFEKASPVTKQKQYEVHQRKTSGGTCDFPESHGEERTWPCMNENENDGLSRIPKDEPGDLHLGQSPDIGVKTSSICDGERTTKKREMVAGKSLPIPSVNGVPASQPNEQILTQSSQPVKVLEKLQPRRNPDAGGCSSKHDQVTISSGHSEKLSAVPKEERRPHNSQARQCIHDRNHASLDKEEKGTNTKGESVQVSSLSMGVPIPEPGQESYAYSTKFEKVEKLQPRRNLDSVVQESHADVGSTPSKAPDKGFDDGYNWRKYGQKLVKGNKFVRSYYKCTYPNCQAKKQVEKSHDGCKSDINYLGNHHHQKPLQSPQVTTTFQVRTPEMPIASTSKPNVEPITKNASSDQHMAETSPKSVVGRSADGLSGATSCSNNDTKDLDDCPDPKRQKRGISSADDDVVNRSSSDSRHVVQTLSEVDLVNDGYRWRKYGQKLVKGNPNPRSYYRCSNTGCPVKKHVERASHDPKLVITTYEGKHVHDIPTSRTVSQSIARGGDANMMSPNGESSSKPEDNSPRPLLRSSSLTC |
| >*SmWRKY21* |
| MMEEVEKANIAAVESCHRVINILNQAQDQNLSGNLVEQTGEAVQRFKKVVSLLNSSCGHARVRKSRKIQPPFPPNIFLETPIFRNDDHRHHQQPKPLHLLQNNPVDTNRRQESVSIAKNSLTLGNPSLELSSHGKNLRLSQQTPLPNYHFLQQQQRFQLQQQQQQAELVYRRSNSGISLNFDSSSCTPTMSSTRSFISSLSMDGSVANVDGSAFRLIGAARSADQASFHHKRRCAGRGEDGSVKCGGSGRCHCSKKRKHRVKRSIKVPAISNKIADIPPDEYSWRKYGQKPIKGSPHPRGYYKCSSMRGCPARKHVERCLEDPSMLIVTYEGEHNHPRLPSQSANA |
| >*SmWRKY22* |
| MEDDWDLHAVVRGCAAATSNTRSLQALYGHQQACSSFSASSFQDLLEPRRNGAVREGLIDLYNYKPLFPNSEIPFSLPVSTISALVGSQDPPPPQTQLRPKHHSFSVSNASKTSASHSPTPRSKRRKTHTKKVCHVAAENLSSDVWSWRKYGQKPIKGSPYPRGYYRCSTSKGCMARKQVERNRSDPGMFIVTYTAEHNHPMPTHRNSLAGSTRNKPADPTGTSFSPEVSPATSLSPPAEKLESSREEELAEDDDDGVFSVSEMALDDDFFAGLEDFTAADPDCASGHLQFPWLSTSATTTTTAGGG |
| >*SmWRKY23* |
| MEDPFPSSLSFLDHYTASGCTLAEMLDYSDEKSSSLGFMELLNTQDHFAAFEEEVEDPSIHPNRNSSEVLNAPQTPNCSSISSESSDGVKDERVNEEEDEEDEEEDDNDQKKKTSKQLKVKKTNQKKKREQRFAFMTKSEVDHLEDGYRWRKYGQKAVKNSPFPRSYYRCTNATCNVKKRVERSCADPSIVVTTYEGQHTHPSPQSLRPHAPPPPSYPGFSLNNNQHQLMNYSLLVNGYMGGGGGGGGGPQQLSFCSQSSPALMANNGLLQDIVPWPAAKREEH |
| >*SmWRKY26* |
| MAPFIKEEEEFDSINSNPISTTIAEKRAGASIAERRAAKCGFNASSVNAALCPPPYFTVPPGLSPSALLDSPVMLPNAEAQMSPTTGTFHPLPMHEGLRQLKITSASNGKKTCSSETNSMSDSNYKEFEHGNKDYVESSEVMMLDDIEKKNAQKEVSKMINSEDGFYWRKYGQKHVKGSEYPRSYYKCTNANCPVTKKVERSHDGHIAEIVYKGTHNHPQPKQMPIDVVDDVSSLSSSTLTAASSSNHDFPDPPDREVPDVEAEAAKKRKRDSEMTMAAVPRSTAREPKVVVQLESEIDILEDGYRWRKYGQKVVKGNPNPRSYYKCTTANCPVRKHVERAANDIKSVITTYEGKHTHAVPSGKNGINSDPAAANPAPPLRAAAAGKQQLVQDYLDRKPMFNYSSGGDLSFGASSPYPLPSFPPFHSMPYASALAYSKLYPEYMPMPMPMPMPMSMPMPMPSSSPSCMPPFRYGGEPQLKPKEEHDQLYATCLAIPNNGTM |
| >*SmWRKY27* |
| MDDDWDLHAVVRGCAANTAAADNPFPSNLPFHGDQNSFDFSVDDRNDHFQGLQEIYQEFCGPPTAALQPQHQEELMQIKPALRLNMQIQENVQFNIGSSPQNFPSAIPQPVRPRRRKNQQMKMVRQMTQEELSADSWAWRKYGQKPIKGSPYPRNYYRCSTSKGCAARKQVERSPNDPEIFVVSYSGEHTHPRPTHRSSLAGSTRSKLASPPPKGGINSAVLSQKGSMASSSSSPATGASFSPDTPSIEAEAAAQNDDVQMAEEDDGAGDEDDENIIMIPSDLMNDDDDMFRGFQDSEDHSSGGGGDFFSMSSPPWTSTSSPGTFNGGD |
| >*SmWRKY28* |
| MSDADNYKMGSQQGLQFSFLADHTSFMYNHPKQQNPSDTMSFFYNTPSPSPAFDPSCSSSEPPLFAVNKNLGENLPTTPNSSEAAAVEEADSCPKHCKAGDLKPKKVKEKKAREARFAFVTKSEVDNLEDGYRWRKYGQKAVKNSPFPRSYYKCTSQKCSVKKLIERSYEDSTMVITTYLGRHNHHSPATLRGRAAVLPPPLQPQFGFHNFPENYFTSIHPNFPQLEINPQFLADQFNMDSSFLQHP |
| >*SmWRKY2-like* |
| MPMGGFDDHVAIMGEWMPPSPNPGAFFLSMMNDDVGARLAVESDSGNRRGPEQQGTSRNGDQKDQSEASFGDDETNNCKAAGEPKLSSRGGLMERIAARAGFNAPRLNTENIRPSDLSKKPEVRSPYLTIPPGLSPTTLLDSPVFLSNSLVLPSPTTGKFSFASSGDYRDSTLMIGDSDKGKENNFEDINASSFAFKPITESASFPYLNATNKTFPSAGFTVHSNTSLQRQSIEPTKSHLANNSTLFQEASFSKSDLGNNGSSNLLQQTHSSGAMAHSPPFDEQNDDGDQRSSGDPNSGGSTADDGYNWRKYGQKHVKGSEYPRSYYKCTHPSCPVKKKVERSQEGHITEIIYKGAHNHAKPPPNRRSTLGSSNALGDMQLDAEEPGTGDPIWSAVQNGNAAGGWKPDNLETTPSALPGQEYNNGGTTAPQAQNGTTYESVDGVDGCSTFSNEEEEDDRATRGSMSLGYDGEGDESESKRRKLETYAPEMGGASRPIREPRVVVQTTSEVDILDDGYRWRKYGQKVVKGNPNPRSYYKCTSAGCNVRKHVERASHDLKSVITTYEGKHNHDVPAARNSGAHVNSGGQNTHPPQQAHAASGNVHRPEPSHLRGSTAWFERPALGSFGLGPGHGFGYRMDQAGLGMGPNQGKLGVHPYLGQQNRAVKEMCYMLPKGEPKVEPVTDAGGLNSSNASSVYEQLMSRLPHMGNINIGREYSESLCELLGLT |
| >*SmWRKY3* |
| MAGNKPPPSSSSSKPPPSITLPPRTSIESLFTGGAGASPGPMSLVSSFFAENDPDNDCRSFSQLLAGAVQSPADEFRFQQNRPAVLAVSHPPGAGTFTVPPGLSPATLLDSPDLFSSSQGHFGLSHHQILAQISGQAQAQAQAQMQFQYSSFPSAPASSISHMQPIPSNASFQQHIPPPLPDTKNIKESSDFSQSDQKFQPANFAVDKPTDDGYNWRKYGQKQVKGSEFPRSYYKCTHPSCPVKKKVERSLDGQITEIIYKGQHNHPQPSKRPKDSGGSNGTMNPQRSSEFGGDRLSKPEEGGAKDQGSSQLTPEQVSGSSDSEEVAGGETRVDDGHEDEPESKRSRIVEVQNPEQASSSHRTVTEPRIIVQTTSEVDLLDDGYRWRKYGQKVVKGNPYPRSYYKCTTAGCNVRKHVERAASDPKAVITTYEGKHNHDVPAAKNSSHGNANAATQFRPQNPVANRPAASRGIEYGNEQQSVALLRFKEEQIT |
| >*SmWRKY30* |
| MSSPPFSGKGMENFPSDSDLKNLVRELSQGKEMAKQLQNHLNLHSSSSRESREFLLHRIMYSYDQALSMLTHNDPAAAASQAPPPPPESPRPVAGSPQSDDSDQDFTDQTSRKRKASDRWSQKVKVGPEVGVEGQLDDGYGWRKYGQKEILGAKHPRGYYRCTYRHAHGCLATKQVQRSDEDPTIFEITYRRKHTCNRATNNPPVPDPRGQDPNPLTQNPQSEPPFNIQTTLNPPFNFSSSSPINPQNDNIFSDRNNALGNYYPNPTNDLNADYGELIPNLRHSDSELSPIVAAMTNSATLDADFPFGPSGFGSVFDFDNSGHYP |
| >*SmWRKY32* |
| MEGANNRSSDALREKVSEDSEPSEERGVENRDGKAEKIDEQSRVSSGSTSDYALGNSSRGESKASQLQTLASVELPVADFRADFDDSSDHLSEVPVEYSLQPVPSGEDMKFLPQDHVKSVKKIVPKDIGGKSSASATKSSSSIASSNPPEQKLRPVENGSNLCIPEQDKQNPKHLSIVPTPRTPSDGYNWRKYGQKQVKSPEGSRSYYRCTFSDCCAKKIECCDISNRLIETVYRSHHNHDPPQKLNSTRENKPSLSSPPVNGSNDPSHLVRHVNDPVPSTPSKEHVVALDEKPETKQESSGSDETTVADTKELYGDGPESKKRQKRNSTGEVESLPKPGKKPKYVVHAAGDVGISGDGYRWRKYGQKMVKGNPHPRNYYRCTSAGCPVRKHIERAVDNSSAVVITYKGIHDHDTPVPRKRHGPKGGIAAATSPVSLNDMQSKSKLHKTQQTQWSVDKEGELTGEALVGGDKASESARTLLSVGFEIKPC |
| >*SmWRKY33* |
| MSSASFTSLLTSDHHSTTTSSAVGRGLGDRIAQRTGSGVPKFKSLPPPSLPITPPSAASPSSWFAIPPGLSPAELLDSPILLSASNILPSPTTGTYPIQGFGWNSSFNTNQQEESKLERNNFSEFSFQTRKPLTSYNLGEETWNYQQSANQNVQSSVDKPITKSEVKQMQSYSPEVAAIQKNDQSCNAAQIEYNHYNQNQSSSQNRKSDDGYNWRKYGQKQVKGSENPRSYYKCTFANCSMKKKVEKDLDGQITEIVYKGNHNHPKPQSTRRSSSSSSASSSVHHQSYSAHFNEISEQSYGQDRIDSSAATPDNSTISIGDDELEQSSQKSRSVGDEIDDEEPHPKRWKCESENDGVSTVGSRAVREPRVVVQTTSDIDILDDGYRWRKYGQKVVKGNPNPRSYYKCTTAGCPVRKHVERASHDPRSVITTYEGKHNHDIPAARGSGNGRPSINAGAAMATRPTPLPNNSTYPIPVSGLRPSLMEPQPGYGMGSYGYSSFGTYDDALFSRAKDEPKDDLFLESLLC |
| >*SmWRKY33* |
| MASSGGSLNPALNSFHYNPTSQYMNSFTDLLTSNKDHYEEKYSFPPLSPPPLSPSFFLDSPALFGSSNALPSPTTGAFAALFAKDDGPAFSDFSFHSQTRPSDPSAALITSKDCVKRQREEESWIRKVEEVPRSYVREQGKSDDGYNWRKYGQKQVKGSENPRSYYKCTFANCPTKKKVERNLDGCVTEIVYKGSHNHGKPHANSPSVSDDQASTSITNSTDDNANDHDAKRWKGDDENESGCGNRAVREPRVVVQTTSEIDILDDGYRWRKYGQKVVKGNPNPRSYYKCTSNGCPVRKHVERAFHDNRAVITTYEGKHNHEVPAARGSACYAASRPQAAACTVQNSDSFAFGNNSYMNQRDNAMSAPAKEEPKDHSFFDLFLD |
| >*SmWRKY35* |
| MCSHYLLKKKKRKMENYQGDLTDIIRATGGAETPAAAPDWHFPANPITFSTNPADDFGDPFAHLRDPLIHIDAPPPLPGFFHGSDVIKPSSVDDSNLGHKIIDDEMKRPPNIFSRMLQISPSPKLPLPLPLPCDSPVAVAPTGLKGVAPMLVPNDHTLISPSNSRGCLMESSLQISSPRNTGIRRRKSQAKKVVCIPAPAPANSRPSGEIVPSDLWAWRKYGQKPIKGSPYPRGYYRCSSSKGCSARKQVERSRTDPNMLVITYTSEHNHPWPTQRNALAGSTRSQPSKKSINPKEEQKESAVKEEVDNNEQSYKPALPESQDSFFAEIEADSLDMLFTQGFDDRDTKALDPFGFYDWGHDHTDS |
| >*SmWRKY4* |
| MAENESPPPASTTAPMTLLSGFFPENDPDIYCRSFSQLLMPSFGQPAEFMFQQNRPAGLVVSPPLAMLTVPPGLSPASLLDSPQGMVGAGEQHDFHSSSTTFSLQRNVPPRVPEISQMKSQLVVDKPADDGYNWRKYGQKQVKGSEFPRSYYKCTRSDCPVKKKVERSLDGQITEIIYKGQHNHPRPARRATDCSTDQDSVSASTDSEADGDEHESKRRRVGETSEQALSRRTVTEPRIIVQTTSEVDLLDDGYRWRKYGQKVVKGNPHPRSYYKCTSQGCNVRKHVERAATDAKAVITTYEGKHNHDIPAAKTSSHSSAAASQLRPVNELAERYGGDDQRLPHMKEEQDSIASSQNNWKIYNS |
| >*SmWRKY40* |
| MEFTSLLNNSLDLNAKPLRFLDESPPLKHQVLESSFIGLGTSVVNVKEEKGALIEELNRVSAENKKLTELLTVMCENYTELRNQLVEQTSKNVNGVENSNAASRKRKAESSNIDALNPESSSSDEESSKKPREEHIKAKISRVCVRTEASDTSLIVKDGYQWRKYGQKVTRDNPCPRAYFKCSFAPTCPVKKKVQRSVEDQSILVASYEGEHNHAPPSKVESNSSGSNKNSNLGSTSVSSTAAPTVTLDLTKPKQESTNATPKLDAPELQHFFVEQMASTLTKDPNFKAALAAAISGKFLQSNNHTDKC |
| >*SmWRKY41* |
| MERVSTWDLKVLTDELAQGMEKAMQLRFHLCSTSHSEAEDLILQRIISTFEKALCILKCGGHVGVAHVGAQAAAPESSISADGSSRSDDTNKNIKEYQEQGYASKKRKLQPTWTEQVKVNSENGLEGPGDDGYSWRKYGQKDILGAKYPRSYYRCTYRQGRNCWATKQVQRSDEDATVFEITYKGTHTCKQFNNVVPPPTSPEKQELKSNNSCYDQLEQNPMLSNFRANLIVNTEVSDIQEMPAYFSFPSTFACHEKENHCYPISALVDDNHLQGTYSPLFYSPATSGSNCFSTATYQMRDHDYGGNDNTDIMSAHASTNNSPIGGMEFSIEPAEIDPNFPFNVPGFFHIIKVQTF |
| >*SmWRKY42* |
| MDRGWGLTLDNSDQVGFFGNKASFGVNLSPRRNSGFIMFPRGREEQPEHREVDFFAVKKEDSQAQPSAATDFHVNTGLQLVTANTGSDQSTVDDGVSYDGEDKRAKNELAQLQIELERMNGENQRLRGMLSQVSGNYASLQMHLVTLMQHQQNSTTQEQEMDDKKCEDERKENGGARQFLELGPEQSNSLSEERTVSGSPHQNNLEVTRNKRSGREESPESESWVPGSKAPRLNNPNPNINTNTNTNTNTNQSIEATMRKARVSVRARSEAPMISDGCQWRKYGQKMAKGNPCPRAYYRCTMAVGCPVRKQVQRCAEDRTILITTYEGTHNHPLPPAAMAMASTTSAAASMLLSGSMPSADGLMNPNFLARTILPCSSNMATISASAPFPTVTLDLTQSPNALQYQRPPPHFQVPFPVSAQNFANQPAPQVFGQALYNQSKFSGLQMSKDVNELPGQPQAQPQPQPSFSDTLSAATAAITADPNFTAALAAAISSIIANTPNNNQP |
| >*SmWRKY44* |
| MQIGSRILPETIAVATVMMEIKEKEKIVIAKPVACRPHFSNLKSLSDLLSAATDASPPAAFTETAGAVIRPTTVRFKPDSAIDLVGVDRMAEAAASHPTKRALESNVACNVVYKPIAKLVSRTTVSQLSNLKSNGISPTQEVADRIQSSNQVTQQSGATLEVHPDLPTQSEQRATTPVENHADETKSSIPANIRDRPSYDGHNWRKYGQKQVKGSEYPRSYYKCTYPNCPVKKKVERTVDGKIAEIVYKGEHNHLKPQPPNHTPSDGHVQKPAYDATRKELQSHSINQQAEAVKAHQGRSENQDLTASSNQSTFSCIPPINYTVASAACNAGPSVSNNSLGECEEVGGFVEAEGGDFRNKRTRSNNQLPRASMAGEASSDPQIVVQNHTDSGIIGDGFRWRKYGQKVVKGKMYPRSYYRCTNPDCNVRKYVERTSEDPGTFITTYEGKHNHAMPIKGANAEASKTSTKN |
| >*SmWRKY46* |
| MENAESWGRDIDGVISQLNHGMKLSNRLKKQLHHNSNPSDCGFLIEKIVSCYDNALRLLSYMDSLQKGEDSTNLLHFTPTTEVYVPKKRKSLERWSEEVHVCCETSSEDQHEDGYNWRKYGQKDILGATHPRAYYRCTYRNTRGCLATKQVQRGEDDPSILKIVYKGKHGCGVNRSKENVVVTAETKVGESNQLMKHESPPLILLEPTSMSTCHLSNFLDPETTSFPLGDLDFLIDFDASFLEASSHDYYLSHVQQSKI |
| >*SmWRKY48* |
| MEEEKRKEAAAKASSNNQSMMINQEISSGSLFDISYAEAEADHLLQKNCSLGFMDVQDFSCPSSAFDDLLHNMVSEPPPEVLTNPATPNASSISSSSTEADRKKNDEEEQEEANKSRKQLKLKKKNQKRQRDPRFAFMTKSEIDHLDDGYRWRKYGQKAVKNSPFPRSYYRCTSPACGVKKRVERSSDDPSIVVTTYEGTHTHSCPITPRGAAAGFGVIPIEASAFHGGASSSLFDMPQILHYPRPLQQQRPYFTNLTLHPPPLSTNANSRVPPPFSSAAAATRDHGLLQDMLPSEMLKEPKGE |
| >*SmWRKY50* |
| MFFNFNFDSAIDAGINPQFQKSHNPNPNPNPNFDEVVHDILNLDDQIYFKDVSDYLMSEENPEETDLSQNKSIYDESSSSIGNKEYRRIVKNYNKEKQDFRVAFRTKSKLEIMDDGFKWRKYGKKMVKSSPNPRNYFKCSNGECSVKKRVERDANDSRFVITTYEGVHNHESPTCVLYCNPKSFLFD |
| >*SmWRKY52* |
| MIMAENYHNTNISGASSGSGGSSAHARGGHGHEPGFDFTNFDAWFDGFPADFSSFSHLLNCMPNELEAESGDASYTSSLSGETTINGESGRERERRGIKEKVAFKTKSEIEILNDGYKWRKYGKKMVKNSPNPRNYYKCSIEGCPVKKRVERDKEDRRYVVTTYEGIHNHEGPTI |
| >*SmWRKY53* |
| MEKVGVSEKKTVITVLTEGKKLANELKRQLHPTKNPTEACDVLVESILSSYENAITLLALIENGGFEPSISIEGSPRSEGSDHNSKKRKTMPRWSEHVRISSGAGGEAQLDDGYNWRKYGQKDILGATHPRAYYRCTHRNTQGCLATKQVQRADEDPTVFEVIYRGKHSCRQERLRQSKERKEQESLACSTLVSNGPSLIKTEKRDVIDSKDEEDLPSFSFPSTPIECENVEPQFFSEPNRFITTSYSPSSFLSSATSESYFSPSPCLVNDFGFGNSLQSSESDFGEIISNPTPITDFSLGDLDISIDEVDFESHFLDEFF |
| >*SmWRKY55* |
| MEEDLILYGCKLAKEMEQNLPMLANQPEALLASCEEIITIFNNVKERLIVSQGNMMSFHGRGGAGIQEWLRTASGSLTPATSATTSVAPEPMDLLSAQAMLRDPTNFHDGAKMKGLLEQQQSSSGLDFMLGGERDISRVGAIDEVHPKTNAPDFIKTSPPRHQRRRKEEGGKYVTTVAAPQMGNLDIPPEDGFTWRKYGQKEILGSKYPRSYYRCTHQKFYDCPAKKQVQRLDNDPFTFELTYRGTHTCHMSSTAPAPPPPEHTLPPPSLPTNSHWLSMHIIQGAAGSSTAPRFPDCPLPVADMADAMFNSGSSSSSMDLIFSSMDEKWDSEVKKD |
| >*SmWRKY56* |
| MEGENFPYFFGSSPFPLSGSSTNVLEQSMQNPSFPSSDQMDLASLLSAGSIEQNPGSTASREVDQNSSRSKSRFGKKKKYVPQRVAFHTRSEEDILDDGYKWRKYGQKSVKNSVHPRSYYRCTHHTCNVKKQIQRLSKDNSVVVTTYEGIHNHPCEKLMETLSPLLKQLQFLSRF |
| >*SmWRKY57* |
| MSGEEKKPDPDFAAASTWRSSDDDAGHHSYFFATTDDYKESSILSEFGWNIPPDGGFADFPPIEPDLAASRADDYVAPVVEASASNPSVSSSSSDDPPEKSSAAAKPPPEVAGKAKKKGQKRIRQPRFAFVTKSEVDHLEDGYRWRKYGQKAVKNSPFPRSYYRCTNSKCMVKKRIERSFEDPSVVITTYEGQHSHYSVGYPRAGLVTQDSNNLPYRPRLPHPPPRLQEPSVGEGLLGDIVRNNGS |
| >*SmWRKY6* |
| MDSARSPPPPTIQFPLNLGRGGADQAAENANDKGKAAVDEMDFFSEKKERSSGGGCGGACSDLYTTEPSSISDFNVNTELLLLTANNGGDGGTSSNSDRRLKSEVGVLKSKLERMNSENERLKEMLNHAKNKYNGLQMHLQTIIQEQQHKGVADQEGKISFHGNGPPNANDVSSFEEDGPTAGGEGNRSPQQKFLSHSASPNSVNEATEATIRKARVSVRAWCEGPMMTDGCQWRKYGQKIAKGNPCPRAYYRCTMAVGCPVRKQVQRCAEDKSILVTTYEGHHNHPLPPTAMAMASTTSSAARMLLSGSMPSADGLMNSSFLARTLLPSSSNLATISTSAPFPTVTLDLTTSPNPLQLPQPPMPFPAPPAAALLPQLLGQALYNQSQFSGLQMSQEMDQNNHQLQNLHSSMNQGQHYNQFNETSSNSIQPNDPSFAAALAAAITSIIGGSSTNNGGNNNNNNNNNNDNNNNSSNDLNGSSFYVNR |
| >*SmWRKY61* |
| MESEYLELYKSVVSEISKGMEQVKECCSNKEQDGFLERMLSSHEEALLILTGRAPQGQCQSGISSLSDPPDSPTGSKDARSRLWKREYFKRRNKIVKIEASSDSELPLEDGYAWRKYGKKAILNAKYPRLRWVWFGWHTYWRESFRRMKAWMDGLTLTENCFRVPFVEGCK |
| >*SmWRKY65* |
| MNSNRFSSSHFARESENSSSPENSEDSPTSAMAKDLKITSKLRRSAVRKKVMSVPIKDIERLRLKGDHVSAPPSDSWAWRKYGQKPIKGSPYPRGYYRCSSSKGCPARKQVERSSADPNMLIVTYSCEHNHPPPSSRNIHHRPPPPAMSVSEEELEEEEEEEDEYDEEGEKPRDLVHNQSQELILDNRFEGNLICGGEFGWLTDFESTSCMILESPILMEERRNTDREMAMIFSMREEEEEDLFAGLGELPECSTVFRRGMAQREAEAGHHRLRGAMCGARR |
| >*SmWRKY7* |
| MAVELMPGYNFTSKMEVSAAQEAAAAGLQSVEKLMRLLSHQQNFQEIQAADDYKAVADVAVYKFKKFISLLDRTPRTGHARFRRGPVPNSPPPPQKTEPAGPSAQTPKTEESAPGSIIYCPTPIQRLPPLPVPHNHQQQVKNVPNPIERKESSTTINFASPATSFMSSLTGDTDSLQPSMSMSSGFQITNLSQVSSAGRPPLSTSSFKRKCSSMDDATAKCGSGSSRCHCPKKRKSRLKRVVRVPAISMKMADIPPDDYSWRKYGQKPIKGSPHPRGYYKCSSVRGCPARKHVERALDDPTMLIVTYEGEHNHALSNMETPALVLESS |
| >*SmWRKY70* |
| MGSLSFDNFHARKKKAMVELVKGKEIAVQLQTLLHKPVSDRGPVSPKQLAVLIYRSFSDTLSELSSCTTPPDSDQIPAAEGSSACSAESPKKKRGVKDRRGCYKRRRTSDSWVTRSPTIEDNYAWRKYGQKTIMTSEYPRSYFRCTHKQEGCKALKQVQRIKGDEIMYQTTYLNHHSCKETLRPPPLLVNSDPIDPNLISFQTPKQDQIQSSKKLTVKCEECVSEDASNEAKSTLEDPWHDITGLDPLGYKPLWFPYQDEVESASLHGLDMEVNQLFDIQNFHYFD |
| >*SmWRKY70-like* |
| MGSLSFDNFHARKKKAMVELVKGKEIAVQLQTLLHKPVSDRGPVSPKQLAVLIYRSFSDTLSELSSCTTPPDSDQIPAAEGSSACSAESPKKKRGVKDRRGCYKRRRTSDSWVTRSPTIEDNYAWRKYGQKTIMTSEYPRSYFRCTHKQEGCKALKQVQRIKGDEIMYQTTYLNHHSCKETLRPPPLLVNSDPIDPNLISFQTPKQDQIQSSKKLTVKCEECVSEGASNEAKSTLEDPWHDITGLDPLGYKPLWFPYQDEVESASLHGLDMEVNQLFDIQNFHYFD |
| >*SmWRKY72A* |
| MKQRFIRFQLFKITSGCYANEVMTSRNMSSTHDKEVNMTRAKLKMEEVREENERLKLRLSQVMKDYNSLKKQLNGITQEDESKKSTNLGSLAASDDDDESELVCLSLGRSSRKKINLENDKLELGLNSRNLRSENSLDESKEEGSSDVRSSPKHSRSNGEDEILQHNPLKKPRVSVRALCNTQTMHDGCQWRKYGQKIAKGNPCPRAYYRCTASSSCPVRKQVQRCIDDMSILITTYEGTHNHPLPASAAPMASATSSAATMLSCGSTPFGYNFPRATISSSQSHPTVILDLTAPKSTTLFSTTRSSPTSFNFSSNFPAINNATYSQPPLFSGTQNDQKIAAAIAHDPNFQSALAAAFTSIVGKNNNNGGMNFQHLNFAQASTSSAGCAASFMNRFGQPVNSQQANFAPLQMGESKSRAMSVADEGEQMKL |
| >*SmWRKY75* |
| MEDLAPKLFPNSNMAPTTLYKTGNLDAFHFGASSHVAEKPKTESFLSEENCDRMKISGSVGSGKKKSEQRVRKPRFAFQTRSQVDILDDGYRWRKYGQKAVKNNKFPRSYYRCTREGCNVKKQVQRLSRDESVVVTTYEGKHTHPVEKPSDNFEQILSQMQIYPPF |
| >*SmWRKY9* |
| MATQQDEEEAEEISATMEPHLSLKLDAQLHSAPSSDTQDPPPILNSHQGFNTQELTVLQMKMNRVKEENEQLRNAVEKTMKDYTDLQTKFVMIQQNSKNKDPNTIESVIEKDDVAPRRRSASEEGAENDGLGLSLRVASSCSNTPEEKREEIMGAFGPTQHTNNMTGIMNHINSPPNKRARVSVRARCDSATMNDGCQWRKYGQKIAKGNPCPRAYYRCTVAPGCPVRKQVQRCLEDKSILITTYEGTHNHPLPVGATAMASTTSATSPSPYMMFDVNNPFHMQNNQVQHSPFYNPLINPSSSFIPNLRPLNPNYYDPTKGIVLDLTNNHPGSSNSMPQLGYSLIPKPPNFNGNTQLFPHPNALLQDQDGGNFDKTTSLSENVSAITSDPKFRVAVAAAISSLINKESQTVPKDGESGGNRDKSWILESSNPIHQSPTE |
| >*SmWRKY76* |
| MAVELLGYTSLNEQMAIQEAASAGLKSMEHLIRAVSHQQHQMQQQHQQQLDCREITDFTVSKFKKVITILNRTGHARFRRAPIQPQPQPQLQIQSSPPTPVQRQELTPAPIQPRNPAQPLSLFSLSPAPPAPQAPAASQPLTLDFTKPSASMGKDGGEMIGKDGLSLSAAVSTSGNSSSTFVSSITGEGSVSNGKGGSPSMFLAPAAPPISASKPPLSGKRCREHNHSDNISGKTSGSSRCHCKKRKSKVKRVIRVPAVSSKVADIPPDEYSWRKYGQKPIKGSPYPRGYYKCSTVRGCPARKHVERATDDPSMLMVTYEGEHRHTQGAMQEISAAGAPAQLVVFESS |
| >*SmWRKY77* |
| MMDELLKLREGSEDDELVRELLDELSPLFMQHYNMESASIFSSAAYSAPMADEIETDSSYSSNGIHGLGHGDYKSEADRISMLEKSYLSKANIHDNINKYTLRIKNCGSVMADDGYKWRKYGQKSIKNSPNPRSYYRCTNPRCNAKKQVERSIEDSETLIITYEGLHLHYTYPLLLLNNQQPDPPFKKHKGPRADPKAHDYAAQTENGLEKMSQQGLLEDVVPLTIRKPLVMDATSSHSSSSTSHDRSSQPSSPASTNSFTYSYLGLM |
| >*SmWRKY78* |
| MDELLKLREGSEDDELVRELLDELSPLFMQPYNMESASIFSSAAYSAPMADEIETDSSYSSNGIHGLGHGDYKSEADRISMLEKSYLSKANIHDNINKYTLRIKNCGSVMADDGYKWRKYGQKSIKNSPNPRSYYRCTNPRCNAKKQVERSIEDSETLIITYEGLHLHYTYPFLLLNNQQPDPPFKKHKGPRADPKAHDYAAQTENGLEKMSQQGLLEDVVPLTIRKPLVMDATSSHSSSSTSHDRSSQPSSPASTNSFTYSYLGLM |
| >*SmWRKY79* |
| MEDRSHSLPHYSHPHSENVTSAHDHVTDAGGSTNGARYRLMSPAKLPISRSTCITIPPGLSPTSFLESPVLLSNIKAEPSPTTGSFFKPQMMQSSGENAAILLEKNFASGNAVDERTSSGFEFRFHTGVSTTSGLSSAGMSIPVGMNRNGLVTHQDQRYSQSAAPSSLASQSHAVARQDSSVYDNIDESNQGAQSNTGTQASVSEHKDSSSSVSAERTSEDGYNWRKYGQKLVKGSEFPRSYYKCTYPNCEVKKIFERSPSGHITEIVYKGSHDHPKPQSARRHNPGALMSIQEDKLDKGLSLAGQEDKMNANNMEQSGSPMLSPLQTNEEGIDGSGSQFHGQNDDAEDDDPFLKRRKMEDGTDVTPVVKPIREPRVVVQTVSEVDILDDGYRWRKYGQKVVRGNPNPRSYYKCTNAGCPVRKHVERASHDPKAVITTYEGKHNHDVPIARNSSHEFAASTAYAGTSKMRAHSISLDLGVGIGPAAGEQKSQEGLDGEAGAGPTQNNQIYEIVNLYGNREAHNFQTPPINASNQCQQNLGRILMGP |
| >*SmWRKY80* |
| MEEVERANSAAVESCYRVIHLLSQPQIQNHYRNLSDQTGDAVNKFKKVVSFLNSSSGHARVRKFKKIQPPLPQNIFLENPMTRTEDQHPKPLSLIQINPLESNPLQEHGSSIKSALTLASPSLELNSLAKNLPQFAQQAADYHLLQQQKQRRIQLQQQQQAEVMYRCNSSGISLNFDGSTCTPTMSSSRSFISSLSVDASVANMDGSAFHLIGSSRSADQGSYHHKRRCSGRGDDGSVKCGGSGRCRCSKKRKNKIKRSIKVPAISTKLADIPPDEYSWRKYGQKPIKGSPHPRGYYKCSSMRGCPARKHVERCLEDSSMLIVTYEGEHNHPNLPSQSANT |
| >*SmWRKY81* |
| MSELVSDMADWSLQAIVRGSSGEFGKMDMDGPDSFLQALTSDHHHQFDQDLRSSFPDIFEGSTGSSGDELEELYKPFYPAPVSHESLPDHTALEFQEQNAAEDKPSSHDQSDQSSVTSPVASVTAVYTPKYKKRKNQHKRVVIQVSAEGLTSDMWAWRKYGQKPIKGSPYPRSYYRCSSSKGCLARKQVEQSCTDPGMFIITYTAEHSHSQPTRRNSLAGTVRQKFPSPKQDKTTENSKSRQQQQQQPELMDVKPRIKQEEDAKPSDEFDVSDFVFNEDFFSGLEEFDEFAQPFPVRPCSWV |
| >*SmWRKY82* |
| MQESDQSHSAADHSHANKKRKMGERVVVAVKIEGKDKKQHRNEGPPSDCWSWRKYGQKPIKGSPYPRGYYRCSTSKGCSAKKQVERCKTDASMLIITYTSTHNHPDPAPSSLSPHTKLTNAHHSHPTTPNQEQESETNPEETLNSGDDRLQFSQSPFVSTSDHAVPFEEILQTPGARYGDEASPLLKSEENDFYDELEELPTSSFFTAFMKSGFCEERITVNLS |
| >*SmWRKY83* |
| MEDDWDLHAVVRGCAAATSNTRSLQALYGHQQACSSFSASSFQDLLEPRRNGGVREGLIDLYNYKPLFPNSEIPFSLPVSTISALVGSQDPPPPQTQLRPKHHSFSVSNASKTSASHSPTPRSKRRKTHTKKVCHVAAENLSSDVWSWRKYGQKPIKGSPYPRGYYRCSTSKGCMARKQVERNRSDPGMFIVTYTAEHNHPMPTHRNSLAGSTRNKPADPIGTSFSPEVSPATSLSPAAEKLESSREEELAEDDDDGVFSVSEMALDDDFFAGLEDFTAADPDCASGHLQFPWLSTSATTTTTAGGG |
| >*SmWRKY84* |
| MEDDWDLHAVVRSCSSSTATATTAATATSSAATAGCQNLSSFEQDCTFSWCNKDPFEPRRSRSFVEDLHELWKPFFPKQQIPISPLSVLQDLSSHQQQQQQQQQKEIKQIISPQKQQLFSIVNGSKPSHHPTPRSKKRKIHSKKVCHVAAESLSSDVWSWRKYGQKPIKGSPYPRGYYRCSTSKGCMARKQVERNRSDPGMFIITYTGDHSHPVPTHRNSLAGSTRHREAKKPEMSPPPAEKMESSREDLADVEEEEEEDDDFFAGLEDFAADGLPPPPAAEATAGGG |
| >*SmWRKY85* |
| MEEKQKESGDEKIAFSDQMMIPESSSGSGFFDLSSTTDHHHKTNSFRFVDMLSVQDFTNPSSIFDDLLHTITAPFPPPPEYSEVVNTPATPNSSSVSSSSTEAAAANDDHKRIDEEEEEQDQEKTRKLQLKPKKKNQKRQREPRFAFMTKSEIDNLDDGYRWRKYGQKAVKNSPFPRSYYRCTSTACGVKKRVERSCEDPTIVVTTYEGTHAHPCPLTPRGSFAVMPERGAMLDGGMLHRYHHQLTPPQPMQHQPYFHNLLTSPLSLITTTANTNANTNTNPNPALFANLFQERPFYPSAAARDDGLLQDMLPSQMFKGQKGEEEE |
| >*SmWRKY86* |
| MQIKPALRLNMKNQENVQFNIGSPPQNFPGAIPQPVRPRRRKNQQMKMVRQMTQEELSADSWAWRKYGQKPIKGSPYPRNYYRCSTSKGCAASKQVERSPKVSYSGEHTHPRPTHQSSLAGSTRSKLASPPPKGGINSAVLSQKGSMASSSSSPATGASFSPDTPTIDAEAAAHNDDVQMAEEDDGAGDEDDENIIMIPSDLMNDDDDMFRGFQDSEDHSSGSGGDFFSMSSPPWTSTSSPGTFNGGD |
| >*SmWRKY87* |
| MHIQENVQFNIGFSPPPQNFPATVIPQPKNQQMKMVRQMAQEELSADSWAWRKYGQKPIKGSPYPRCSTRNKLVSLPPKGLDVEYGFFFVVVARRRSEFFSGYSVDGRRTQNEDVQMAEEDDGAVDEDDENIIMIPSDLMIDDDDMFGSGFQDSKYHSSGGGGDFFLMSPRRGHRPAPPAHSTAVTKGTWKSLMDHCFLEMDGKTG |
| >*SmWRKY88* |
| MSEDHLREFYYHHPYNEDRHGGAATFPYSGADAHLHMFDPSSYLSFTEFLHGPTDHNSLSAACGPSPPTSKEEEKAAGGNETPATPNSSISSSSTEVAAAEEDSNKSKKEKESFIAHGDEENSKKEGSKGKKKGEQKKERQPRFAFMTKSEVDHLEDGYRWRKYGQKAVKNSPYPRSYYRCTTQKCPVKKRVERSFQDPSIVITTYEGQHNHHVPATLRGSMVGLFGQSMLGPPAFPQELLLQMPHLYGGNSSNLYNNNEQQQLQQQQQQFHDYGLLQDIIPPLFPKRES |
| >*SmWRKY89* |
| MSNDPNKINPYYPYFEFHHDHVDHNHTMLSEGFAFPIVTDHSNFSPFMYQNPNEFHHILQGYNTLSPPPDPPPLCNNTSNNSNDVAGGSGGDGGGGENIPVTPNSSASISSQEAGAEEDSSKSKKDVRDGKSKNVKAKEKEGKKQRQARFAFMTKSEVDNLEDGYRWRKYGQKAVKNSPFPRSYYRCTSQKCNVKKRIERSYQDATVVITTYEGQHNHHSPAVLRGSAAAMLAPSLFSPQPAFPTFPQDMFYPSSATNFYTQLTDSGRHVPPPPPPPA |
| >*SmWRKY90* |
| MSEDFTADFYYRHPFQDDQRRTTTTAAFTYSGSHSSAYANDISSTFSADHSSVVDNNLQMFDPTYASFAEFLHGSNDNTSFSAAFGLSPSSSVAVEDQHTPAVEAARGGGETTPATPNSSISSSSAEAAAADEDLNKGKKEAKETLEDADGDSSKKDSKTKKKAEKKQRQPRYAFMTKSEVDHLEDGYRWRKYGQKAVKNSPYPRSYYRCTTQKCPVKKRVERSYEDPSIVITTYEGQHNHHVPATLRGNAAAMFNPSMLAPPLNIPSEGQGFPQELLLQMPHLYGGGGGGGASNMFHQQSFSNPQFHQFVDYGLLQDIIPSVFPKEEP |
| >*SmWRKY91* |
| MAQTVGDAQPSSRAKPTIQVPPRGSVGFLYANGSGPGFSPGPMTLVSSFFPEQSPFSFSQLLAGAMASPLAAKPGFPPAYDSWKDGKEKSICDAGSNSEAGVGRKWDRPVNLVVPPPPQLQVESMGMSPLFMVPPGLSPSGLMSPLQSPFGMSHQQALAHVTAQAALSHSIMHMQEEFQHSSSAEAIVNHSSSAKTEALTQQKNPSPLHLENTKKELAEVSQSSKAAAGDKPANDGYNWRKYGQKHVKASECPRSYYKCTHPNCPVKKKVERSLNGHISEITYKGQHNHDPPKPTKREKDSSVLDTSTNSQANSVLASEDQTETERLNENQNFATTSQQSFDPYAVANHKKDVKEAAIVVDEGDADEPAAKRRTLDIGQSVPASSHQAVPDSKIVLQTRSEVDLLDDGYKWRKYGQKVVKGNPHPRSYYRCTYAGCNVRKHVERASADPKSVITTYEGKHNHDIPIGKHGSSHGAATQHPKTQKVAAVSKDISEIGYGNKDQIPTTLQLKEEQIAA |
| >*SmWRKY92* |
| MGETSVEDAAAAKPKPTIVVPPRGSMVESLFTSGLGAMGFSPGPMTLVSSFFSDQGPFSFSQLLAGAIASPKLRDYANEESWGDSSESGKTSDDAGFKRNRPMTLVVAAPESLSPPFMLPPGLTPSGLLNSPGFLSPLQSPFGMSHQQALAHVTAQAAFSQSYMQMHAELQRSPADTSTEALVSHSSPSPSETLTEQINSAPRQLESSKIESSEVSQSDKKAAYVTTGDKPASDGYNWRKYGQKHVKASECPRSYYKCTYANCPVKKKVERSHDGRVSEIVYKGEHNHLPPQSNKRKKDTSVALDREVNNVDENVQELQNDETEEGPVIVIDDENDDEPLAKRRSSDEGHSVPASSHQTITESKIVVQTRSEVDLLDDGYKWRKYGQKVVKGNPHPRSYYRCTYAGCSVRKQVERSSADPKAVITSYEGKHSHEIPAGKYMSGPAANAQMQRVVTAKAPSISREMEFGNQAPITLLLKEEEIAV |
| >*SmWRKY93* |
| MEKTSMQFIDLNSKPMHNPSETSHDELIQELSRMKWENKKLNEILAVVCRNYHDFTKIDCVEELYASRKRKSDQLQSSGHIFRGCCGGGECSPPGRPREIKSNTSRVHVRIDPSDTSLVVKDGYQWRKYGQKVTRDNPSPRAYYKCSLSPSCPVKKKVQRSAGDPSLLVAIYEGQHNHQPSGAEIPAATAAAAEAPAAAANSTYLSDPTICSRIENEESEIQQVLVEQMASSLTRNHSFTAALVAAITGRILDDGSEENDDSYSISRVISE |
| >*SmWRKY94* |
| MEFSSLLDTSLNLNAKPPPLPPPPPKQEGESQSFMAFGRNEERGALVEEINRMSAENKKLTEMLSMMCENYSGLRNQLAEYRSKKRKSNDNVESSSSDEDSSNKTLIKPNIISHSHVRTQASDTSLIVKDGYQWRKYGQKVTRDNPSPRAYFKCSFAPTCPVKKKVQRSVEDQSIVIATYEGEHNHPHPSKVEAPPNRSSPSLSPLAPRITLDLTNSTPSSDDLRGKAQVQHCLVEQMASTLTKDPTFKAALAAAISGKFLQQNKW |
| >*SmWRKY95* |
| MENNLEASTLSIDLNSNPNEAPKSGFEETSLREMKSLFKDAQDDELIQELCRVKSENKKLSNMLTIVSRNLSEFFKQMQYEEEELSRTRKRKADDINNQHLQNISPTHSADLPNVIKSNISRVYVRIDPSDVSLVVKDGYQWRKYGQKVTRDNPSPRAYYKCSFAPACPAKKKVQRSVDDSSILVATYEGQHHHHSRPAPADGGAPSPCGGATTAGDEYCSKSSQGASDEIHQFLVEKMASSLTRNHNFTTALAAAITGRILDEVILSETTAVVDDTNNISNSIGFSM |
| >*SmWRKY96* |
| MESACSLEYKTVINELVQGMEKAKQLHSHLCSTSPSRAQDLLMQRILSSYEKALLILNPKEHTPPPSAATVDSPISVVDGICTSEDLNNSFRDNQDYNASKKRKMQPTWMEQVKVNPENGLEGPADDGYSWRKYGQKDILGARYPRSYYRCTYRLVQNCWATKQVQRSDDDPTVFEITYKGTHTCNQSTNAPVVPPPVSQEKQELQQQSQALLKLKASLRVNTDTGNSETMPAHFSFPSTYELCDADNHYISFSDLVGDGNLGAYSPLFLSPATSETNYFSPAPHHMASFGGSQGYQHTESDIADIVSAHASTTNSPIGSMDFPIDPLELDPNFPFNTSGFFR |
| >*SmWRKY97* |
| MDAPLMIMADDHSYPIKPGSSLGQAHEPGFEVSDFFEFNDWIEEAPSFSSPLSAASYDSQNPSYAPNAGSSWSSSSSSSSFLEGAITRDSGCGREKEMREKVAFKTKSEVEVLDDGFKWRKYGKKMVKNSPNPRNYYRCSVEGCPVKKRVERDNEDPLYVVTTYEGIHNHKGPHQV |
| >*SmWRKY98* |
| MLKLKSTTNTTPQSHQNPNSPSLNCDHDLSLFGNQLNDPLYFNIANYLMFDEDFGSNDNNISCEKSVVDCPNGSFTSVPFANNIKPRRVVNKYKVQDDCRFAFRTKTQLEVMDDGYKWRKYGKKMVKNSPNPRNYYKCSSGGCNVKKRVERDCLDPNYVITTYQGTHNHASPTCLLHCSLPPPPTLI |
| >*SmWRKY99* |
| MDGKDTMDSELSWGFGGGGESESRSSYLFGGDCKDISSILSEFGWNIPADSCGVFGCERGFADFDRIDSDLAGDAADSRAAAGASAPPRDDGTCRSSGEAMAEESSMSASVLASNPSVSSSSSEDLSAASGNSAAAAAPNPPPDTASKAKKQGQKRIREPRFAFVTKSEIDHLEDGYRWRKYGQKAVKNSPFPRSYYRCTNTKCRVKKRVERSWEDPSVVITTYEGQHCHYSAGFPRGGGILPPPPPQPQPRQGLLGDIVPPAMRNG |
| >*SmWRKY100* |
| MEKGWGLALENSDRVGFFGSKSVFEYSQSPRLNNRSTMFPAAPPPAGEHEVVVGEVDFFSAKKRPVDEVKKEINETRDLNIGLQLVTASDRSKVDDVSSDAEDRRAKIEMGQIEVELERMKAENQRLKGMVTHISNNYTTLQMQLHRLMQHQQNEIVERKSEEKEENGVVLVPRQFLELNKQISDEQSGSSSEERTVSGNKRINERDESEGSAPNKSPKPAPPEPLPDATMRKARVSVRARSEAPMISDGCQWRKYGQKMAKGNPCPRAYYRCTMAVGCPVRKQVQRCAEDRTILITTYEGNHSHPLPPAAMSMASTTSSAASMLLAGSMPSGDGGGLMNPSFLTRAMLPCSSNMATISASAPFPTITLDLTHSPSPHFQLPLAPATPPSFHQVLGQVVQNQSKFSGLQMSQDMSQLRPSYADTLSAATAAITSDPTFTAALAAAISSILGGAQPNNDGAAAANHNNAHVT |
| >SmWRKY101 |
| MATRGGLSFDPDHHSITRLFHHQAKTHFHNNQLKLQQILPRFPAPPPPSPPLMDAPVKSPPTTTIQFPVSHGDHGKRKVLDEMDFFADKNEGGGGGGGRDDDEEENVGIEHHRLDFKVNTGLHLLTTANTGSEESGVDDGMSSIPENKRSKIEMAFLQSEMERMNSENARLKEMLDQMTNNYNGLQMHVLTMMHQRTDNQTGGATAEESKNGHDNGLLVPRQFMDLGFGGNEEGSLSPPEGRIGRDMPPAHDKDVVGGDEPCSESKVQKLAHSPRSVDQATEATMRKARVSVRARSEAAMITDGCQWRKYGQKLAKGNPCPRAYYRCTMAAGCPVRKQVQRCADDTSILITTYEGNHNHPLPPAAMAMASTTSSAARMLLSGSMPSADGIMNSNFLARTLLPCSSSMATISASAPFPTITLDLTQSPNPLQFPKPSNQFPLSFLNPSQNMAAAGSSAAAALLPQIFGQALYNQSKFSGLQMSSAQELDQGAHEHLSSPLPPSLHQACQQNQLSETVNALATDPNFTAALAAAISSLIGGGGPSPNNGGPNPNNNNGNNNNNNNNNGNGNVTSSNNSSNGNNKLNSSGFQVN |
| >SmWRKY102 |
| MADLVGCKAVAEKEEEVIGIREENLRLKTLLQKIEMDYKNLQMKLSDFQQESKKSAEVSGDDEDQELVSLRLGSKSTMDRNDVVKAGSGAPMKTAVKTQRNGDDETSQTSVKKARVCVRARCDTPTMNDGCQWRKYGQKIAKGNPCPRAYYRCTVSPSCPVRKQVQRFIEDTSILITTYEGSHNHPLPLAATAMASTTSAAASMLLSGSSASQPSAAPLNRYGQPPPYFISNSSSNSSPTITLDLIANPSTLYSSTSPTNPRCPTTNLSFTSMDSNTVPAVWGGGGGGNSYHHLLQQQKFETSYVGNPNYQAPSQQALTETLTKVISSDPSFRSAIAAVISTMAANGRCSEDGRRDEG |
| >SmWRKY103 |
| MEGRFDDEHNEDSALTPPPENTADSPISGDEEAYAPSPKKSRRGGNKRVVMVPIGDGDGSRSKAEVYPPPDSWSWRKYGQKPIKGSPYPRGYYRCSSSKGCPARKQVERSRLDPTTLLVTYSCNHNHPLPTTTKNHHHHHHHHHHQNPLLPATAASPAAKPAITSSSSDTASPPPAASFPPEECAASSHQPDIEPCPADNGFVELAGEFGWLCNVGPTIMDGATLVGPTWGAQADVEFSTPIGEEDKLLFGDLGDLPESAMVFRRHRVEAPCCAGGTG |
| >SmWRKY104 |
| MDAGFNRHRHRFVNELDDSDNSAGNSGGDSPRSAVSTDTKIASTSSSKRSRRAIQKRVVSVPIKDVEGLRQKGDQISAPPSDSWAWRKYGQKPIKGSPYPRGYYRCSSSKGCPARKQVERSRLDPKMLVVTYYCEHNHNWPVSKSSHHKTAAARNVKTPISEEEEEEEEEEEEEEGEEVEEEKKPRINLSDQSRDEVAIGAADNKFSTNNHGGEGLSIQNGEFGWFADFECANFTMLESPMLAEERISTDSEMAMIFTMGDQEDESLFADLGELPECSTVFRRGMVQREVEQRRPGLATTG |
| >SmWRKY105 |
| MAVELMMSYGNSSFASQSEETAAAEAASGLQSVEKLIRFFSSAQKPAEDYAAVADAAVSKFKRAISLLGQARTGHARFRRAPTAAASSCSSPNSGEDRGGYSKIYCPTPIQQVPPPDYLHKAAEDSKTISFSYSHESSFINSPPSAAAFHVIGTKPPLSSSSSFKRSCASSGNGISGKCGGRCQCSKRRKLRVKRVVMVPAISMKMADIPPDDYSWRKYGQKPIKGSPHPRGYYKCSSVRGCPARKHVERAVDDPTMLIVTYEGDHNHTLSIAETPATMILESS |
| >SmWRKY106 |
| MASSSCVHRNAVPERVMTELTRGREIADRIRLMLRETGFDGSAGSVFPVQSLVSQLLDTFTHSLAMLRDGGESDEASQAPSAAKLEDSGDSCKTPAAKDRRGCYKRKRSSETRIKESSDLFEDGHAWRKYGQKSILNAKHPRNYYRCTHKFDQKCLATKQVQKIQDEPPLYRTTYNGQHTCNKSALHHHVVPASPDSSSVMWSFNSIKQEEEIQMKTPDEYYISPHHFSPMSSDVYSCSHSIEDVDDIVGSFEDYFEFETLS |
| >SmWRKY107 |
| MDISLHKSVLEEAVKEESKPSESNGDEESCVDDIVLHQFGAGKRAQDNDGMKSPSPNKKNSNCSNQGLAITNMESRPSPESESRSSSSAKKDQLDDQLGSTKAEMDEVMEENQRLKMYLDGILKDYRTLQMQYRDVMQQEATTKLSPHHHDLMEAAADDGLIDLSLAMSSIDKKRKTPKMNDDIEILDDNKQGLSLGLDCKFDLPEKSPIKPSPTLTPENSIEEVKEEAGETWPPKGVKNARDAGGEDEISQQNPTKRARVSVRVRCDTPTMNDGCQWRKYGQKISKGNPCPRAYYRCTVAPSCPVRKQVQRCAEDMTILITTYEGTHNHPLPMSATAMASTTSAAASMLMSGSSTSGTGPSTPSTTTTASNLHGLNFYLSDNSRTTKPTFYLPNPSISSSPSYPTITLDLTSSSSSSSSHLNRLGSGGGFAPRYSTTNLNFSSLESNPLPNSYNNAMLTYGQQSFNRNQNAANSLSFGSQPYETLYQSYLQKSISNPNPNQQHLGADTIAAATKAITSDPSFQTVLAAALTSIIGGSGGTAAANNHNAAEKSSGGDAFPILSSFPPTSNATKCAPSFMNKSSSSSSSQQPGLSTLLSPPFPFSSSNKSKSNSPADNRDHV |
| >SmWRKY108 |
| MEEHLLFAPSQTPSFFPDSFPEMDQIRSQTLQNSVPKFKEDCKVDNSRNKRGNYERRKMSFPQRFAFQTRSQEDILDDGYRWRKYGQKSVKNSKFPRSYYRCTHPTCNVKKQVERLSKDSSIVMTTYEGTHNHPSEKLMQSLTPLLQQIHFLTSANKSSIV |
| >SmWRKY109 |
| MDNFAAAMSSSSSSFFNMMMTSQPHDIDHHQVLIQHNNNGFMEQNQSIPTAAEPENEAKGGKKKGEKKSRKPRFAFQTRSQVDILDDGYRWRKYGQKAVKNNTFPRSYYRCTHQGCNVKKQVQRLSKDEGIVVTTYEGVHSHPIQKSTDNFDHILSQMQIYTSL |
| >>SmWRKY110 |
| MEDDRRIVDELECSSSSMMMMKSAEKKGAMEKKSRKPRFAFQTRSQVDILDDGYRWRKYGQKAVKNNTFPRSYYRCTHQGCNVKKQIQRLSKDEGIVVTTYEGMHSHPIQNSTDNFEHILTQMQIYSSF |
| >AtWRKY1 |
| MAEVGKVLASDMELDHSNETKAVDDVVATTDKAEVIPVAVTRTETVVESLESTDCKELEKLVPHTVASQSEVDVASPVSEKAPKVSESSGALSLQSGSEGNSPFIREKVMEDGYNWRKYGQKLVKGNEFVRSYYRCTHPNCKAKKQLERSAGGQVVDTVYFGEHDHPKPLAGAVPINQDKRSDVFTAVSKGEQRIDIVSLIYKLCIVSYDIMFVEKTSGSSVQTLRQTEPPKIHGGLHVSVIPPADDVKTDISQSSRITGDNTHKDYNSPTAKRRKKGGNIELSPVERSTNDSRIVVHTQTLFDIVNDGYRWRKYGQKSVKGSPYPRSYYRCSSPGCPVKKHVERSSHDTKLLITTYEGKHDHDMPPGRVVTHNNMLDSEVDDKEGDANKTPQSSTLQSITKDQHVEDHLRKKTKTNGFEKSLDQGPVLDEKLKEEIKERSDANKDHAANHAKPEAKSDDKTTVCQEKAVGTLESEEQKPKTEPAQS |
| >AtWRKY7 |
| MTVELMMSSYSGGGGGGDGFPAIAAAAKMEDTALREAASAGIHGVEEFLKLIGQSQQPTEKSQTEITAVTDVAVNSFKKVISLLGRSRTGHARFRRAPASTQTPFKQTPVVEEEVEVEEKKPETSSVLTKQKTEQYHGGGSAFRVYCPTPIHRRPPLSHNNNNNQNQTKNGSSSSSPPMLANGAPSTINFAPSPPVSATNSFMSSHRCDTDSTHMSSGFEFTNPSQLSGSRGKPPLSSASLKRRCNSSPSSRCHCSKKRKSRVKRVIRVPAVSSKMADIPSDEFSWRKYGQKPIKGSPHPRGYYKCSSVRGCPARKHVERALDDAMMLIVTYEGDHNHALVLETTTMNHDKTL |
| >AtWRKY2 |
| MAGFDENVAVMGEWVPRSPSPGTLFSSAIGEEKSSKRVLERELSLNHGQVIGLEEDTSSNHNKDSSQSNVFRGGLSERIAARAGFNAPRLNTENIRTNTDFSIDSNLRSPCLTISSPGLSPATLLESPVFLSNPLAQPSPTTGKFPFLPGVNGNALSSEKAKDEFFDDIGASFSFHPVSRSSSSFFQGTTEMMSVDYGNYNNRSSSHQSAEEVKPGSENIESSNLYGIETDNQNGQNKTSDVTTNTSLETVDHQEEEEEQRRGDSMAGGAPAEDGYNWRKYGQKLVKGSEYPRSYYKCTNPNCQVKKKVERSREGHITEIIYKGAHNHLKPPPNRRSGMQVDGTEQVEQQQQQRDSAATWVSCNNTQQQGGSNENNVEEGSTRFEYGNQSGSIQAQTGGQYESGDPVVVVDASSTFSNDEDEDDRGTHGSVSLGYDGGGGGGGGEGDESESKRRKLEAFAAEMSGSTRAIREPRVVVQTTSDVDILDDGYRWRKYGQKVVKGNPNPRSYYKCTAPGCTVRKHVERASHDLKSVITTYEGKHNHDVPAARNSSHGGGGDSGNGNSGGSAAVSHHYHNGHHSEPPRGRFDRQVTTNNQSPFSRPFSFQPHLGPPSGFSFGLGQTGLVNLSMPGLAYGQGKMPGLPHPYMTQPVGMSEAMMQRGMEPKVEPVSDSGQSVYNQIMSRLPQI |
| >AtWRKY23 |
| MEFTDFSKTSFYYPSSQSVWDFGDLAAAERHSLGFMELLSSQQHQDFATVSPHSFLLQTSQPQTQTQPSAKLSSSIIQAPPSEQLVTSKVESLCSDHLLINPPATPNSSSISSASSEALNEEKPKTEDNEEEGGEDQQEKSHTKKQLKAKKNNQKRQREARVAFMTKSEVDHLEDGYRWRKYGQKAVKNSPFPRSYYRCTTASCNVKKRVERSFRDPSTVVTTYEGQHTHISPLTSRPISTGGFFGSSGAASSLGNGCFGFPIDGSTLISPQFQQLVQYHHQQQQQELMSCFGGVNEYLNSHANEYGDDNRVKKSRVLVKDNGLLQDVVPSHMLKEE |
| >AtWRKY3 |
| MAEKEEKEPSKLKSSTGVSRPTISLPPRPFGEMFFSGGVGFSPGPMTLVSNLFSDPDEFKSFSQLLAGAMASPAAAAVAAAAVVATAHHQTPVSSVGDGGGSGGDVDPRFKQSRPTGLMITQPPGMFTVPPGLSPATLLDSPSFFGLFSPLQGTFGMTHQQALAQVTAQAVQGNNVHMQQSQQSEYPSSTQQQQQQQQQASLTEIPSFSSAPRSQIRASVQETSQGQRETSEISVFEHRSQPQNADKPADDGYNWRKYGQKQVKGSDFPRSYYKCTHPACPVKKKVERSLDGQVTEIIYKGQHNHELPQKRGNNNGSCKSSDIANQFQTSNSSLNKSKRDQETSQVTTTEQMSEASDSEEVGNAETSVGERHEDEPDPKRRNTEVRVSEPVASSHRTVTEPRIIVQTTSEVDLLDDGYRWRKYGQKVVKGNPYPRSYYKCTTPDCGVRKHVERAATDPKAVVTTYEGKHNHDVPAARTSSHQLRPNNQHNTSTVNFNHQQPVARLRLKEEQIT |
| >AtWRKY4 |
| MSEKEEAPSTSKSTGAPSRPTLSLPPRPFSEMFFNGGVGFSPGPMTLVSNMFPDSDEFRSFSQLLAGAMSSPATAAAAAAAATASDYQRLGEGTNSSSGDVDPRFKQNRPTGLMISQSQSPSMFTVPPGLSPAMLLDSPSFLGLFSPVQGSYGMTHQQALAQVTAQAVQANANMQPQTEYPPPSQVQSFSSGQAQIPTSAPLPAQRETSDVTIIEHRSQQPLNVDKPADDGYNWRKYGQKQVKGSEFPRSYYKCTNPGCPVKKKVERSLDGQVTEIIYKGQHNHEPPQNTKRGNKDNTANINGSSINNNRGSSELGASQFQTNSSNKTKREQHEAVSQATTTEHLSEASDGEEVGNGETDVREKDENEPDPKRRSTEVRISEPAPAASHRTVTEPRIIVQTTSEVDLLDDGYRWRKYGQKVVKGNPYPRSYYKCTTPGCGVRKHVERAATDPKAVVTTYEGKHNHDLPAAKSSSHAAAAAQLRPDNRPGGLANLNQQQQQQPVARLRLKEEQTT |
| >AtWRKY10 |
| MSDFDENFIEMTSYWAPPSSPSPRTILAMLEQTDNGLNPISEIFPQESLPRDHTDQSGQRSGLRERLAARVGFNLPTLNTEENMSPLDAFFRSSNVPNSPVVAISPGFSPSALLHTPNMVSDSSQIIPPSSATNYGPLEMVETSGEDNAAMMMFNNDLPYQPYNVDLPSLEVFDDIATEESFYIPSYEPHVDPIGTPLVTSFESELVDDAHTDIISIEDSESEDGNKDDDDEDFQYEDEDEDQYDQDQDVDEDEEEEKDEDNVALDDPQPPPPKRRRYEVSNMIGATRTSKTQRIILQMESDEDNPNDGYRWRKYGQKVVKGNPNPRSYFKCTNIECRVKKHVERGADNIKLVVTTYDGIHNHPSPPARRSNSSSRNRSAGATIPQNQNDRTSRLGRAPPTPTPPTPPPSSYTPEEMRPFSSLATEIDLTEVYMTGISMLPNIPVYENSGFMYQNDEPTMNAMPDGSDVYDGIMERLYFKFGVDM |
| >AtWRKY12 |
| MEGGGRRVFSNYDLQQVTSSSTTIQENMNFLVPFEETNVLTFFSSSSSSSLSSPSFPIHNSSSTTTTHAPLGFSNNLQGGGPLGSKVVNDDQENFGGGTNNDAHSNSWWRSNSGSGDMKNKVKIRRKLREPRFCFQTKSDVDVLDDGYKWRKYGQKVVKNSLHPRSYYRCTHNNCRVKKRVERLSEDCRMVITTYEGRHNHIPSDDSTSPDHDCLSSF |
| >AtWRKY19 |
| MSEKEELPLTLTSIGAATATSDYHQRVGSSGEGISSSSSDVDPRFMQNSPTGLMISQSSSMCTVPPGMAATPPISSGSGLSQQLNNSSSSKLCQVEGCQKGARDASGRCISHGGGRRCQKPDCQKGAEGKTVYCKAHGGGRRCEYLGCTKGAEGSTDFCIAHGGGRRCNHEDCTRSAWGRTEFCVKHGGGARCKTYGCGKSASGPLPFCRAHGGGKKCSHEDCTGFARGRSGLCLMHGGGKRCQRENCTKSAEGLSGLCISHGGGRRCQSIGCTKGAKGSKMFCKACITKRPLTIDGGGNMGGVTTGDALNYLKAVKDKFEDSEKYDTFLEVLNDCKHQGVDTSGVIARLKDLFKGHDDLLLGFNTYLSKEYQITILPEDDFPIDFLDKVEGPYEMTYQQAQTVQANANMQPQTEYPSSSAVQSFSSGQPQIPTSAPDSSLLAKSNTSGITIIEHMSQQPLNVDKQVNDGYNWQKYGQKKVKGSKFPLSYYKCTYLGCPSKRKVERSLDGQVAEIVYKDRHNHEPPNQGKDGSTTYLSGSSTHINCMSSELTASQFSSNKTKIEQQEAASLATTIEYMSEASDNEEDSNGETSEGEKDEDEPEPKRRITEVQVSELADASDRTVREPRVIFQTTSEVDNLDDGYRWRKYGQKVVKGNPYPRFSSSKDYDVVIRYGRADISNEDFISHLRASLCRRGISVYEKFNEVDALPKCRVLIIVLTSTYVPSNLLNILEHQHTEDRVVYPIFYRLSPYDFVCNSKNYERFYLQDEPKKWQAALKEITQMPGYTLTDKSESELIDEIVRDALKVLCSADKVNMIGMDMQVEEILSLLCIESLDVRSIGIWGTVGIGKTTIAEEIFRKISVQYETCVVLKDLHKEVEVKGHDAVRENFLSEVLEVEPHVIRISDIKTSFLRSRLQRKRILVILDDVNDYRDVDTFLGTLNYFGPGSRIIMTSRNRRVFVLCKIDHVYEVKPLDIPKSLLLLDRGTCQIVLSPEVYKTLSLELVKFSNGNPQVLQFLSSIDREWNKLSQEVKTTSPIYIPGIFEKSCCGLDDNERGIFLDIACFFNRIDKDNVAMLLDGCGFSAHVGFRGLVDKSLLTISQHNLVDMLSFIQATGREIVRQESADRPGDRSRLWNADYIRHVFINDTGTSAIEGIFLDMLNLKFDANPNVFEKMCNLRLLKLYCSKAEEKHGVSFPQGLEYLPSKLRLLHWEYYPLSSLPKSFNPENLVELNLPSSCAKKLWKGKKARFCTTNSSLEKLKKMRLSYSDQLTKIPRLSSATNLEHIDLEGCNSLLSLSQSISYLKKLVFLNLKGCSKLENIPSMVDLESLEVLNLSGCSKLGNFPEISPNVKELYMGGTMIQEIPSSIKNLVLLEKLDLENSRHLKNLPTSIYKLKHLETLNLSGCISLERFPDSSRRMKCLRFLDLSRTDIKELPSSISYLTALDELLFVDSRRNSPVVTNPNANSTELMPSESSKLEILGTPADNEVVVGGTVEKTRGIERTPTILVKSREYLIPDDVVAVGGDIKGLRPPVLQLQPAMKLSHIPRGSTWDFVTHFAPPETVAPPSSSSEAREEEVETEETGAMFIPLGDKETCSFTVNKGDSSRTISNTSPIYASEGSFITCWQKGQLLGRGSLGSVYEGISADGDFFAFKEVSLLDQGSQAHEWIQQVEGGIALLSQLQHQNIVRYRGTTKDESNLYIFLELVTQGSLRKLYQRNQLGDSVVSLYTRQILDGLKYLHDKGFIHRNIKCANVLVDANGTVKLADFGLAKVMSLWRTPYWNWMAPEVIVLKSFPLF |
| >AtWRKY20 |
| MILLPEPSPTTGSLFKPRPVHISASSSSYTGRGFHQNTFTEQKSSEFEFRPPASNMVYAELGKIRSEPPVHFQGQGHGSSHSPSSISDAAGSSSELSRPTPPCQMTPTSSDIPAGSDQEESIQTSQNDSRGSTPSILADDGYNWRKYGQKHVKGSEFPRSYYKCTHPNCEVKKLFERSHDGQITDIIYKGTHDHPKPQPGRRNSGGMAAQEERLDKYPSSTGRDEKGSGVYNLSNPNEQTGNPEVPPISASDDGGEAAASNRNKDEPDDDDPFSKRRRMEGAMEITPLVKPIREPRVVVQTLSEVDILDDGYRWRKYGQKVVRGNPNPRSYYKCTAHGCPVRKHVERASHDPKAVITTYEGKHDHDVPTSKSSSNHEIQPRFRPDETDTISLNLGVGISSDGPNHASNEHQHQNQQLVNQTHPNGVNFRFVHASPMSSYYASLNSGMNQYGQRETKNETQNGDISSLNNSSYPYPPNMGRVQSGP |
| >AtWRKY25 |
| MSSTSFTDLLGSSGVDCYEDDEDLRVSGSSFGGYYPERTGSGLPKFKTAQPPPLPISQSSHNFTFSDYLDSPLLLSSSHSLISPTTGTFPLQGFNGTTNNHSDFPWQLQSQPSNASSALQETYGVQDHEKKQEMIPNEIATQNNNQSFGTERQIKIPAYMVSRNSNDGYGWRKYGQKQVKKSENPRSYFKCTYPDCVSKKIVETASDGQITEIIYKGGHNHPKPEFTKRPSQSSLPSSVNGRRLFNPASVVSEPHDQSENSSISFDYSDLEQKSFKSEYGEIDEEEEQPEMKRMKREGEDEGMSIEVSKGVKEPRVVVQTISDIDVLIDGFRWRKYGQKVVKGNTNPRSYYKCTFQGCGVKKQVERSAADERAVLTTYEGRHNHDIPTALRRS |
| >AtWRKY26 |
| MGSFDRQRAVPKFKTATPSPLPLSPSPYFTMPPGLTPADFLDSPLLFTSSNILPSPTTGTFPAQSLNYNNNGLLIDKNEIKYEDTTPPLFLPSMVTQPLPQLDLFKSEIMSSNKTSDDGYNWRKYGQKQVKGSENPRSYFKCTYPNCLTKKKVETSLVKGQMIEIVYKGSHNHPKPQSTKRSSSTAIAAHQNSSNGDGKDIGEDETEAKRWKREENVKEPRVVVQTTSDIDILDDGYRWRKYGQKVVKGNPNPRSYYKCTFTGCFVRKHVERAFQDPKSVITTYEGKHKHQIPTPRRGPVLRLLGKTET |
| >AtWRKY32 |
| MASLRCFRELSRRATTVFSINQTRSISSFHGIEFSGTSISHGTVIPNRSLTRNLPWYSHWYRSQDRCFSSNTKDTDEDEESSEGEDDDEEEGEDFEDSADMEVEREYSPAEKVEEAEEIGYKVMGPLKPSERLFKPYEPVFAIVQIGSHQFKVSNGDSIFTEKLKFCDINDKLELTKVLLLGSASQTIIGRPILPDATVHAVVEEHALDEKVLIFKKKRRKNYRRTRGHRQELTKLRITDIQGIEKPEPKIVHKPSKEAVTEQTKAELVA |
| >AtWRKY33 |
| MAASFLTMDNSRTRQNMNGSANWSQQSGRTSTSSLEDLEIPKFRSFAPSSISISPSLVSPSTCFSPSLFLDSPAFVSSSANVLASPTTGALITNVTNQKGINEGDKSNNNNFNLFDFSFHTQSSGVSAPTTTTTTTTTTTTTNSSIFQSQEQQKKNQSEQWSQTETRPNNQAVSYNGREQRKGEDGYNWRKYGQKQVKGSENPRSYYKCTFPNCPTKKKVERSLEGQITEIVYKGSHNHPKPQSTRRSSSSSSTFHSAVYNASLDHNRQASSDQPNSNNSFHQSDSFGMQQEDNTTSDSVGDDEFEQGSSIVSRDEEDCGSEPEAKRWKGDNETNGGNGGGSKTVREPRIVVQTTSDIDILDDGYRWRKYGQKVVKGNPNPRSYYKCTTIGCPVRKHVERASHDMRAVITTYEGKHNHDVPAARGSGYATNRAPQDSSSVPIRPAAIAGHSNYTTSSQAPYTLQMLHNNNTNTGPFGYAMNNNNNNSNLQTQQNFVGGGFSRAKEEPNEETSFFDSFMP |
| >AtWRKY34 |
| MAGIDNKAAVMGEWFDCSTTNHRKRSKAELGREFSLNYIKNEDSLQTTFQESSRGALRERIAARSGFNAPWLNTEDILQSKSLTISSPGLSPATLLESPVFLSNPLLSPTTGKLSSVPSDKAKAELFDDITTSLAFQTISGSGLDPTNIALEPDDSQDYEERQLGGLGDSMACCAPADDGYNWRKYGQKLVKGSEYPRSYYKCTHPNCEAKKKVERSREGHIIEIIYTGDHIHSKPPPNRRSGIGSSGTGQDMQIDATEYEGFAGTNENIEWTSPVSAELEYGSHSGSMQVQNGTHQFGYGDAAADALYRDENEDDRTSHMSVSLTYDGEVEESESKRRKLEAYATETSGSTRASREPRVVVQTTSDIDILDDGYRWRKYGQKVVKGNPNPRSYYKCTANGCTVTKHVERASDDFKSVLTTYIGKHTHVVPAARNSSHVGAGSSGTLQGSLATQTHNHNVHYPMPHSRSEGLATANSSLFDFQSHLRHPTGFSVYIGQSELSDLSMPGLTIGQEKLTSLQAPDIGDPTGLMLQLAAQPKVEPVSPQQGLDLSASSLICREMLSRLRQI |
| >AtWRKY44 |
| MSCDDDSDSRNYVVYKPKAKLVSKATVSALANMLQGNRQQTWRQSEAVSYGKSVSQGTHRAGPNLVQKVPSFTESETSTGDRSSVDGYNWRKYGQKQVKGSECPRSYYKCTHPKCPVKKKVERSVEGQVSEIVYQGEHNHSKPSCPLPRRASSSISSGFQKPPKSIASEGSMGQDPNNNLYSPLWNNQSNDSTQNRTEKMSEGCVITPFEFAVPRSTNSNPGTSDSGCKSSQCDEGELDDPSRSKRRKNEKQSSEAGVSQGSVESDSLEDGFRWRKYGQKVVGGNAYPRSYYRCTSANCRARKHVERASDDPRAFITTYEGKHNHHLLLSPPSSSTLPFNSPQLSKQTI |
| >AtWRKY45 |
| MEDRRCDVLFPCSSSVDPRLTEFHGVDNSAQPTTSSEEKPRSKKKKKEREARYAFQTRSQVDILDDGYRWRKYGQKAVKNNPFPRSYYKCTEEGCRVKKQVQRQWGDEGVVVTTYQGVHTHAVDKPSDNFHHILTQMHIFPPFCLKE |
| >AtWRKY58 |
| MAVEDDVSLIRTTTLVAPTRPTITVPHRPPAIETAAYFFGGGDGLSLSPGPLSFVSSLFVDNFPDVLTPDNQRTTSFTHLLTSPMFFPPQSSAHTGFIQPRQQSQPQPQRPDTFPHHMPPSTSVAVHGRQSLDVSQVDQRARNHYNNPGNNNNNRSYNVVNVDKPADDGYNWRKYGQKPIKGCEYPRSYYKCTHVNCPVKKKVERSSDGQITQIIYKGQHDHERPQNRRGGGGRDSTEVGGAGQMMESSDDSGYRKDHDDDDDDDEDDEDLPASKIRRIDGVSTTHRTVTEPKIIVQTKSEVDLLDDGYRWRKYGQKVVKGNPHPRSYYKCTTPNCTVRKHVERASTDAKAVITTYEGKHNHDVPAARNGTAAATAAAVGPSDHHRMRSMSGNNMQQHMSFGNNNNTGQSPVLLRLKEEKITI |
| >AtWRKY18 |
| MDGSSFLDISLDLNTNPFSAKLPKKEVSVLASTHLKRKWLEQDESASELREELNRVNSENKKLTEMLARVCESYNELHNHLEKLQSRQSPEIEQTDIPIKKRKQDPDEFLGFPIGLSSGKTENSSSNEDHHHHHQQHEQKNQLLSCKRPVTDSFNKAKVSTVYVPTETSDTSLTVKDGFQWRKYGQKVTRDNPSPRAYFRCSFAPSCPVKKKVQRSAEDPSLLVATYEGTHNHLGPNASEGDATSQGGSSTVTLDLVNGCHRLALEKNERDNTMQEVLIQQMASSLTKDSKFTAALAAAISGRLMEQSRT |
| >AtWRKY40 |
| MDQYSSSLVDTSLDLTIGVTRMRVEEDPPTSALVEELNRVSAENKKLSEMLTLMCDNYNVLRKQLMEYVNKSNITERDQISPPKKRKSPAREDAFSCAVIGGVSESSSTDQDEYLCKKQREETVVKEKVSRVYYKTEASDTTLVVKDGYQWRKYGQKVTRDNPSPRAYFKCACAPSCSVKKKVQRSVEDQSVLVATYEGEHNHPMPSQIDSNNGLNRHISHGGSASTPVAANRRSSLTVPVTTVDMIESKKVTSPTSRIDFPQVQKLLVEQMASSLTKDPNFTAALAAAVTGKLYQQNHTEK |
| >AtWRKY60 |
| MDYDPNTNPFDLHFSGKLPKREVSASASKVVEKKWLVKDEKRNMLQDEINRVNSENKKLTEMLARVCEKYYALNNLMEELQSRKSPESVNFQNKQLTGKRKQELDEFVSSPIGLSLGPIENITNDKATVSTAYFAAEKSDTSLTVKDGYQWRKYGQKITRDNPSPRAYFRCSFSPSCLVKKKVQRSAEDPSFLVATYEGTHNHTGPHASVSRTVKLDLVQGGLEPVEEKKERGTIQEVLVQQMASSLTKDPKFTAALATAISGRLIEHSRT |
| >AtWRKY6 |
| MDRGWSGLTLDSSSLDLLNPNRISHKNHRRFSNPLAMSRIDEEDDQKTRISTNGSEFRFPVSLSGIRDREDEDFSSGVAGDNDREVPGEVDFFSDKKSRVCREDDEGFRVKKEEQDDRTDVNTGLNLRTTGNTKSDESMIDDGESSEMEDKRAKNELVKLQDELKKMTMDNQKLRELLTQVSNSYTSLQMHLVSLMQQQQQQNNKVIEAAEKPEETIVPRQFIDLGPTRAVGEAEDVSNSSSEDRTRSGGSSAAERRSNGKRLGREESPETESNKIQKVNSTTPTTFDQTAEATMRKARVSVRARSEAPMISDGCQWRKYGQKMAKGNPCPRAYYRCTMATGCPVRKQVQRCAEDRSILITTYEGNHNHPLPPAAVAMASTTTAAANMLLSGSMSSHDGMMNPTNLLARAVLPCSTSMATISASAPFPTVTLDLTHSPPPPNGSNPSSSAATNNNHNSLMQRPQQQQQQMTNLPPGMLPHVIGQALYNQSKFSGLQFSGGSPSTAAFSQSHAVADTITALTADPNFTAALAAVISSMINGTNHHDGEGNNKNQ |
| >AtWRKY9 |
| MGFDFSTSKSKAKRQKRIEVRFASPLMGIDLSLKLEAEEKKKEIEGSKHSRENKEDEEHDASGDEDEQMVKEDEDDSSSLGLRTREEENEREELLQLQIQMESVKEENTRLRKLVEQTLEDYRHLEMKFPVIDKTKKMDLEMFLGVQGKRCVDITSKARKRGAERSPSMEREIGLSLSLEKKQKQEESKEAVQSHHQRYNSSSLDMNMPRIISSSQGNRKARVSVRARCETATMNDGCQWRKYGQKTAKGNPCPRAYYRCTVAPGCPVRKQVQRCLEDMSILITTYEGTHNHPLPVGATAMASTASTSPFLLLDSSDNLSHPSYYQTPQAIDSSLITYPQNSSYNNRTIRSLNFDGPSRGDHVSSSQNRLNWMM |
| >AtWRKY36 |
| MIKEETVSYFQTFDGVMAESDKEEELDATKAKVEKVREENEKLKLLLSTILNNYNSLQMQVSKVLGQQQGASSMELDHIDRQDENNDYDVDISLRLGRSEQKISKKEENKVDKISTKNVEESKDKRSALGFGFQIQSYEASKLDDLCRQVKLANAENKCVSSRKDVKSVRNENHQDVLEEHEQTGLKKTRVCVKASCEDPSINDGCQWRKYGQKTAKTNPLPRAYYRCSMSSNCPVRKQVQRCGEEETSAFMTTYEGNHDHPLPMEASHMAAGTSAAASLLQSGSSSSSSSTSASLSYFFPFHHFSISTTNSHPTVTLDLTRPNYPNQLPDDYPLSSSSFSLNFSSPDPPPPSSHDHTLNFSGLRTQAPLSTDSLLARYRTRLSGQQ |
| >AtWRKY42 |
| MFRFPVSLGGGPRENLKPSDEQHQRAVVNEVDFFRSAEKRDRVSREEQNIIADETHRVHVKRENSRVDDHDDRSTDHINIGLNLLTANTGSDESMVDDGLSVDMEEKRTKCENAQLREELKKASEDNQRLKQMLSQTTNNFNSLQMQLVAVMRQQEDHHHLATTENNDNVKNRHEVPEMVPRQFIDLGPHSDEVSSEERTTVRSGSPPSLLEKSSSRQNGKRVLVREESPETESNGWRNPNKVPKHHASSSICGGNGSENASSKVIEQAAAEATMRKARVSVRARSEAPMLSDGCQWRKYGQKMAKGNPCPRAYYRCTMAVGCPVRKQVQRCAEDRTILITTYEGNHNHPLPPAAMNMASTTTAAASMLLSGSTMSNQDGLMNPTNLLARTILPCSSSMATISASAPFPTITLDLTESPNGNNPTNNPLMQFSQRSGLVELNQSVLPHMMGQALYYNQQSKFSGLHMPSQPLNAGESVSAATAAIASNPNFAAALAAAITSIINGSNNQQNGNNNNSNVTTSNVDNRQ |
| >AtWRKY47 |
| MEEHIQDRREIAFLHSGEFLHGDSDSKDHQPNESPVERHHESSIKEVDFFAAKSQPFDLGHVRTTTIVGSSGFNDGLGLVNSCHGTSSNDGDDKTKTQISRLKLELERLHEENHKLKHLLDEVSESYNDLQRRVLLARQTQVEGLHHKQHEDVPQAGSSQALENRRPKDMNHETPATTLKRRSPDDVDGRDMHRGSPKTPRIDQNKSTNHEEQQNPHDQLPYRKARVSVRARSDATTVNDGCQWRKYGQKMAKGNPCPRAYYRCTMAVGCPVRKQVQRCAEDTTILTTTYEGNHNHPLPPSATAMAATTSAAAAMLLSGSSSSNLHQTLSSPSATSSSSFYHNFPYTSTIATLSASAPFPTITLDLTNPPRPLQPPPQFLSQYGPAAFLPNANQIRSMNNNNQQLLIPNLFGPQAPPREMVDSVRAAIAMDPNFTAALAAAISNIIGGGNNDNNNNTDINDNKVDAKSGGSSNGDSPQLPQSCTTFSTN |
| >AtWRKY61 |
| MDEAKEENRRLKSSLSKIKKDFDILQTQYNQLMAKHNEPTKFQSKGHHQDKGEDEDREKVNEREELVSLSLGRRLNSEVPSGSNKEEKNKDVEEAEGDRNYDDNEKSSIQGLSMGIEYKALSNPNEKLEIDHNQETMSLEISNNNKIRSQNSFGFKNDGDDHEDEDEILPQNLVKKTRVSVRSRCETPTMNDGCQWRKYGQKIAKGNPCPRAYYRCTIAASCPVRKQVQRCSEDMSILISTYEGTHNHPLPMSATAMASATSAAASMLLSGASSSSSAAADLHGLNFSLSGNNITPKPKTHFLQSPSSSGHPTVTLDLTTSSSSQQPFLSMLNRFSSPPSNVSRSNSYPSTNLNFSNNTNTLMNWGGGGNPSDQYRAAYGNINTHQQSPYHKIIQTRTAGSSFDPFGRSSSSHSPQINLDHIGIKNIISHQVPSLPAETIKAITTDPSFQSALATALSSIMGGDLKIDHNVTRNEAEKSP |
| >AtWRKY72 |
| MEVLLKLPSSESPLKDKFGSVQIHEANKGDGDHQELESAKAEMSEVKEENEKLKGMLERIESDYKSLKLRFFDIIQQEPSNTATKNQNMVDHPKPTTTDLSSFDQERELVSLSLGRRSSSPSDSVPKKEEKTDAISAEVNADEELTKAGLTLGINNGNGGEPKEGLSMENRANSGSEEAWAPGKVTGKRSSPAPASGGDADGEAGQQNHVKRARVCVRARCDTPTMNDGCQWRKYGQKIAKGNPCPRAYYRCTVAPGCPVRKQVQRCADDMSILITTYEGTHSHSLPLSATTMASTTSAAASMLLSGSSSSPAAEMIGNNLYDNSRFNNNNKSFYSPTLHSPLHPTVTLDLTAPQHSSSSSSSLLSLNFNKFSNSFQRFPSTSLNFSSTSSTSSNPSTLNLPAIWGNGYSSYTPYPYNNVQFGTSNLGKTVQNSQSLTETLTKALTSDPSFHSVIAAAISTMVGSNGEQQIVGPRHSISNNIQQTNTTNNNKGCGGYFSSLLMSNIMASNQTGASLDQPSSQLPPFSMFKNSSSSSSTTNFVNKEEKS |
| >AtWRKY8 |
| MSHEIKDLNNYHYTSSYNHYNINNQNMINLPYVSGPSAYNANMISSSQVGFDLPSKNLSPQGAFELGFELSPSSSDFFNPSLDQENGLYNAYNYNSSQKSHEVVGDGCATIKSEVRVSASPSSSEADHHPGEDSGKIRKKREVRDGGEDDQRSQKVVKTKKKEEKKKEPRVSFMTKTEVDHLEDGYRWRKYGQKAVKNSPYPRSYYRCTTQKCNVKKRVERSYQDPTVVITTYESQHNHPIPTNRRTAMFSGTTASDYNPSSSPIFSDLIINTPRSFSNDDLFRVPYASVNVNPSYHQQQHGFHQQESEFELLKEMFPSVFFKQEP |
| >AtWRKY13 |
| MGAINQGISLFDESQTVINPINTNHLGFFFSFPSHSTLSSSSSSSSSSPSSLVSPFLGHNSLNSFLHNNPSSFISHPQDSINLMTNLPETLISSLSSSKQRDDHDGFLNLDHHRLTGSISSQRPLSNPWAWSCQAGYGSSQKNNHGSEIDVDDNDDEVGDGGGINDDDNGRHHHHDTPSRHDKHNTASLGVVSSLKMKKLKTRRKVREPRFCFKTLSEVDVLDDGYRWRKYGQKVVKNTQHPRSYYRCTQDKCRVKKRVERLADDPRMVITTYEGRHLHSPSNHLDDDSLSTSHLHPPLSNFFW |
| >AtWRKY24 |
| MDREDINPMLSRLDVENNNTFSSFVDKTLMMMPPSTFSGEVEPSSSSSWYPESFHVHAPPLPPENDQIGEKGKELKEKRSRKVPRIAFHTRSDDDVLDDGYRWRKYGQKSVKHNAHPRSYYRCTYHTCNVKKQVQRLAKDPNVVVTTYEGVHNHPCEKLMETLNPLLRQLQFLSSFSNL |
| >AtWRKY28 |
| MSNETRDLYNYQYPSSFSLHEMMNLPTSNPSSYGNLPSQNGFNPSTYSFTDCLQSSPAAYESLLQKTFGLSPSSSEVFNSSIDQEPNRDVTNDVINGGACNETETRVSPSNSSSSEADHPGEDSGKSRRKRELVGEEDQISKKVGKTKKTEVKKQREPRVSFMTKSEVDHLEDGYRWRKYGQKAVKNSPYPRSYYRCTTQKCNVKKRVERSFQDPTVVITTYEGQHNHPIPTNLRGSSAAAAMFSADLMTPRSFAHDMFRTAAYTNGGSVAAALDYGYGQSGYGSVNSNPSSHQVYHQGGEYELLREIFPSIFFKQEP |
| >AtWRKY43 |
| MNGLVDSSRDKKMKNPRFSFRTKSDADILDDGYRWRKYGQKSVKNSLYPRSYYRCTQHMCNVKKQVQRLSKETSIVETTYEGIHNHPCEELMQTLTPLLHQLQFLSKFT |
| >AtWRKY48 |
| MEKKKEEDHHHQQQQQQQKEIKNTETKIEQEQEQEQKQEISQASSSSNMANLVTSSDHHPLELAGNLSSIFDTSSLPFPYSYFEDHSSNNPNSFLDLLRQDHQFASSSNSSSFSFDAFPLPNNNNNTSFFTDLPLPQAESSEVVNTTPTSPNSTSVSSSSNEAANDNNSGKEVTVKDQEEGDQQQEQKGTKPQLKAKKKNQKKAREARFAFLTKSDIDNLDDGYRWRKYGQKAVKNSPYPRSYYRCTTVGCGVKKRVERSSDDPSIVMTTYEGQHTHPFPMTPRGHIGMLTSPILDHGATTASSSSFSIPQPRYLLTQHHQPYNMYNNNSLSMINRRSSDGTFVNPGPSSSFPGFGYDMSQASTSTSSSIRDHGLLQDILPSQIRSDTINTQTNEENKK |
| >AtWRKY49 |
| MEEEGYQWARRCGNNAVEDPFVYEPPLFFLPQDQHHMHGLMPNEDFIANKFVTSTLYSGPRIQDIANALALVEPLTHPVREISKSTVPLLERSTLSKVDRYTLKVKNNSNGMCDDGYKWRKYGQKSIKNSPNPRSYYKCTNPICNAKKQVERSIDESNTYIITYEGFHFHYTYPFFLPDKTRQWPNKKTKIHKHNAQDMNKKSQTQEESKEAQLGELTNQNHPVNKAQENTPANLEEGLFFPVDQCRPQQGLLEDVVAPAMKNIPTRDSVLTAS |
| >AtWRKY50 |
| MNDADTNLGSSFSDDTHSVFEFPELDLSDEWMDDDLVSAVSGMNQSYGYQTSDVAGALFSGSSSCFSHPESPSTKTYVAATATASADNQNKKEKKKIKGRVAFKTRSEVEVLDDGFKWRKYGKKMVKNSPHPRNYYKCSVDGCPVKKRVERDRDDPSFVITTYEGSHNHSSMN |
| >AtWRKY51 |
| MNISQNPSPNFTYFSDENFINPFMDNNDFSNLMFFDIDEGGNNGLIEEEISSPTSIVSSETFTGESGGSGSATTLSKKESTNRGSKESDQTKETGHRVAFRTRSKIDVMDDGFKWRKYGKKSVKNNINKRNYYKCSSEGCSVKKRVERDGDDAAYVITTYEGVHNHESLSNVYYNEMVLSYDHDNWNQHSLLRS |
| >AtWRKY56 |
| MEGVDNTNPMLTLEEGENNNPFSSLDDKTLMMMAPSLIFSGDVGPSSSSCTPAGYHLSAQLENFRGGGGEMGGLVSNNSNNSDHNKNCNKGKGKRTLAMQRIAFHTRSDDDVLDDGYRWRKYGQKSVKNNAHPRSYYRCTYHTCNVKKQVQRLAKDPNVVVTTYEGVHNHPCEKLMETLSPLLRQLQFLSRVSDL |
| >AtWRKY57 |
| MNDPDNPDLSNDDSAWRELTLTAQDSDFFDRDTSNILSDFGWNLHHSSDHPHSLRFDSDLTQTTGVKPTTVTSSCSSSAAVSVAVTSTNNNPSATSSSSEDPAENSTASAEKTPPPETPVKEKKKAQKRIRQPRFAFMTKSDVDNLEDGYRWRKYGQKAVKNSPFPRSYYRCTNSRCTVKKRVERSSDDPSIVITTYEGQHCHQTIGFPRGGILTAHDPHSFTSHHHLPPPLPNPYYYQELLHQLHRDNNAPSPRLPRPTTEDTPAVSTPSEEGLLGDIVPQTMRNP |
| >AtWRKY59 |
| MNYPSNPNPSSTDFTEFFKFDDFDDTFEKIMEEIGREDHSSSPTLSWSSSEKLVAAEITSPLQTSLATSPMSFEIGDKDEIKKRKRHKEDPIIHVFKTKSSIDEKVALDDGYKWRKYGKKPITGSPFPRHYHKCSSPDCNVKKKIERDTNNPDYILTTYEGRHNHPSPSVVYCDSDDFDLNSLNNWSFQTANTYSFSHSAPY |
| >AtWRKY68 |
| MENVGVGMPFYDLGQTRVYPLLSDFHDLSAERYPVGFMDLLGVHRHTPTHTPLMHFPTTPNSSSSEAVNGDDEEEEDGEEQQHKTKKRFKFTKMSRKQTKKKVPKVSFITRSEVLHLDDGYKWRKYGQKPVKDSPFPRNYYRCTTTWCDVKKRVERSFSDPSSVITTYEGQHTHPRPLLIMPKEGSSPSNGSASRAHIGLPTLPPQLLDYNNQQQQAPSSFGTEYINRQEKGINHDDDDDHVVKKSRTRDLLDGAGLVKDHGLLQDVVPSHIIKEEY |
| >AtWRKY31 |
| MFRFPVSLGGSRDEDRHDQITPLDDHRVVVDEVDFFSEKRDRVSRENINDDDDEGNKVLIKMEGSRVEENDRSRDVNIGLNLLTANTGSDESTVDDGLSMDMEDKRAKIENAQLQEELKKMKIENQRLRDMLSQATTNFNALQMQLVAVMRQQEQRNSSQDHLLAQESKAEGRKRQELQIMVPRQFMDLGPSSGAAEHGAEVSSEERTTVRSGSPPSLLESSNPRENGKRLLGREESSEESESNAWGNPNKVPKHNPSSSNSNGNRNGNVIDQSAAEATMRKARVSVRARSEAAMISDGCQWRKYGQKMAKGNPCPRAYYRCTMAGGCPVRKQVQRCAEDRSILITTYEGNHNHPLPPAATAMASTTTAAASMLLSGSMSSQDGLMNPTNLLARAILPCSSSMATISASAPFPTITLDLTNSPNGNNPNMTTNNPLMQFAQRPGFNPAVLPQVVGQAMYNNQQQSKFSGLQLPAQPLQIAATSSVAESVSAASAAIASDPNFAAALAAAITSIMNGSSHQNNNTNNNNVATSNNDSRQ |
| >AtWRKY71 |
| MDDHVEHNYNTSLEEVHFKSLSDCLQSSLVMDYNSLEKVFKFSPYSSPFQSVSPSVNNPYLNLTSNSPVVSSSSNEGEPKENTNDKSDQMEDNEGDLHGVGESSKQLTKQGKKKGEKKEREVRVAFMTKSEIDHLEDGYRWRKYGQKAVKNSPYPRSYYRCTTQKCNVKKRVERSFQDPSIVITTYEGKHNHPIPSTLRGTVAAEHLLVHRGGGGSLLHSFPRHHQDFLMMKHSPANYQSVGSLSYEHGHGTSSYNFNNNQPVVDYGLLQDIVPSMFSKNES |
| >AtWRKY75 |
| MEGYDNGSLYAPFLSLKSHSKPELHQGEEESSKVRSEGCSKSVESSKKKGKKQRYAFQTRSQVDILDDGYRWRKYGQKAVKNNKFPRSYYRCTYGGCNVKKQVQRLTVDQEVVVTTYEGVHSHPIEKSTENFEHILTQMQIYSSF |
| >AtWRKY11 |
| MAVDLMRFPKIDDQTAIQEAASQGLQSMEHLIRVLSNRPEQQHNVDCSEITDFTVSKFKTVISLLNRTGHARFRRGPVHSTSSAASQKLQSQIVKNTQPEAPIVRTTTNHPQIVPPPSSVTLDFSKPSIFGTKAKSAELEFSKENFSVSLNSSFMSSAITGDGSVSNGKIFLASAPLQPVNSSGKPPLAGHPYRKRCLEHEHSESFSGKVSGSAYGKCHCKKSRKNRMKRTVRVPAISAKIADIPPDEYSWRKYGQKPIKGSPHPRGYYKCSTFRGCPARKHVERALDDPAMLIVTYEGEHRHNQSAMQENISSSGINDLVFASA |
| >AtWRKY15 |
| MAVELMTRNYISGVGADSFAVQEAAASGLKSIENFIGLMSRDSFNSDQPSSSSASASASAAADLESARNTTADAAVSKFKRVISLLDRTRTGHARFRRAPVHVISPVLLQEEPKTTPFQSPLPPPPQMIRKGSFSSSMKTIDFSSLSSVTTESDNQKKIHHHQRPSETAPFASQTQSLSTTVSSFSKSTKRKCNSENLLTGKCASASSSGRCHCSKKRKIKQRRIIRVPAISAKMSDVPPDDYSWRKYGQKPIKGSPHPRGYYKCSSVRGCPARKHVERAADDSSMLIVTYEGDHNHSLSAADLAGAAVADLILESS |
| >AtWRKY17 |
| MTVDIMRLPKMEDQTAIQEAASQGLKSMEHLIRVLSNRPEERNVDCSEITDFTVSKFKKVISLLNRSGHARFRRGPVHSPPSSSVPPPVKVTTPAPTQISAPAPVSFVQANQQSVTLDFTRPSVFGAKTKSSEVVEFAKESFSVSSNSSFMSSAITGDGSVSKGSSIFLAPAPAVPVTSSGKPPLSGLPYRKRCFEHDHSEGFSGKISGSGNGKCHCKKSRKNRMKRTVRVPAVSAKIADIPPDEYSWRKYGQKPIKGSPHPRGYYKCSTFRGCPARKHVERALDDSTMLIVTYEGEHRHHQSTMQEHVTPSVSGLVFGSA |
| >AtWRKY21 |
| MEEIEGTNRAAVESCHRVLNLLHRSQQQDHVGFEKNLVSETREAVIRFKRVGSLLSSSVGHARFRRAKKLQSHVSQSLLLDPCQQRTTEVPSSSSQKTPVLRSGFQELSLRQPSDSLTLGTRSFSLNSNAKAPLLQLNQQTMPPSNYPTLFPVQQQQQQQQQQQQQEQQQQQQQQQQQFHERLQAHHLHQQQQLQKHQAELMLRKCNGGISLSFDNSSCTPTMSSTRSFVSSLSIDGSVANIEGKNSFHFGVPSSTDQNSLHSKRKCPLKGDEHGSLKCGSSSRCHCAKKRKHRVRRSIRVPAISNKVADIPPDDYSWRKYGQKPIKGSPYPRGYYKCSSMRGCPARKHVERCLEDPAMLIVTYEAEHNHPKLPSQAITT |
| >AtWRKY39 |
| MEEVEAANRSAIESCHGVLNLLSQRTSDPKSLTVETGEVVSKFKRVASLLTRGLGHGKFRSTNKFRSSFPQHIFLESPICCGNDLSGDYTQVLAPEPLQMVPASAVYNEMEPKHQLGHPSLMLSHKMCVDKSFLELKPPPFRAPYQLIHNHQQIAYSRSNSGVNLKFDGSGSSCYTPSVSNGSRSFVSSLSMDASVTDYDRNSFHLTGLSRGSDQQHTRKMCSGSLKCGSRSKCHCSKKRKLRVKRSIKVPAISNKIADIPPDEYSWRKYGQKPIKGSPHPRGYYKCSSVRGCPARKHVERCIDETSMLIVTYEGEHNHSRILSSQSAHT |
| >AtWRKY74 |
| MEEVEAANKAAVESCHGVLNLLSQQTNDSKSIMVETREAVCKFKRVSSLLSRGLGQRKIKKLNNNNYKFSSSLLPQHMFLESPVCSNNAISGCIPILAPKPLQIVPAGPPPLMLFNQNMCLDKSFLELKPPSSRAVDPKPYQFIHTHQQGVYSRSKSGLNLKFDGSIGASCYSPSISNGSRSFVSSLSMDGSVTDYDRNSFHLIGLPQGSDHISQHSRRTSCSGSLKCGSKSKCHCSKKRKLRVKRSIKVPAISNKIADIPPDEYSWRKYGQKPIKGSPHPRGYYKCSSVRGCPARKHVERCVEETSMLIVTYEGEHNHSRILSSQSAHT |
| >AtWRKY14 |
| MCSVSELLDMENFQGDLTDVVRGIGGHVLSPETPPSNIWPLPLSHPTPSPSDLNINPFGDPFVSMDDPLLQELNSITNSGYFSTVGDNNNNIHNNNGFLVPKVFEEDHIKSQCSIFPRIRISHSNIIHDSSPCNSPAMSAHVVAAAAAASPRGIINVDTNSPRNCLLVDGTTFSSQIQISSPRNLGLKRRKSQAKKVVCIPAPAAMNSRSSGEVVPSDLWAWRKYGQKPIKGSPFPRGYYRCSSSKGCSARKQVERSRTDPNMLVITYTSEHNHPWPIQRNALAGSTRSSTSSSSNPNPSKPSTANVNSSSIGSQNTIYLPSSTTPPPTLSSSAIKDERGDDMELENVDDDDDNQIAPYRPELHDHQHQPDDFFADLEELEGDSLSMLLSHGCGGDGKDKTTASDGISNFFGWSGDNNYNNYDDQDSRSL |
| >AtWRKY16 |
| MTESEQIVYISCIEEVRYSFVSHLSKALQRKGVNDVFIDSDDSLSNESQSMVERARVSVMILPGNRTVSLDKLVKVLDCQKNKDQVVVPVLYGVRSSETEWLSALDSKGFSSVHHSRKECSDSQLVKETVRDVYEKLFYMERIGIYSKLLEIEKMINKQPLDIRCVGIWGMPGIGKTTLAKAVFDQMSGEFDAHCFIEDYTKAIQEKGVYCLLEEQFLKENAGASGTVTKLSLLRDRLNNKRVLVVLDDVRSPLVVESFLGGFDWFGPKSLIIITSKDKSVFRLCRVNQIYEVQGLNEKEALQLFSLCASIDDMAEQNLHEVSMKVIKYANGHPLALNLYGRELMGKKRPPEMEIAFLKLKECPPAIFVDAIKSSYDTLNDREKNIFLDIACFFQGENVDYVMQLLEGCGFFPHVGIDVLVEKSLVTISENRVRMHNLIQDVGRQIINRETRQTKRRSRLWEPCSIKYLLEDKEQNENEEQKTTFERAQVPEEIEGMFLDTSNLSFDIKHVAFDNMLNLRLFKIYSSNPEVHHVNNFLKGSLSSLPNVLRLLHWENYPLQFLPQNFDPIHLVEINMPYSQLKKLWGGTKDLEMLKTIRLCHSQQLVDIDDLLKAQNLEVVDLQGCTRLQSFPATGQLLHLRVVNLSGCTEIKSFPEIPPNIETLNLQGTGIIELPLSIVKPNYRELLNLLAEIPGLSGVSNLEQSDLKPLTSLMKISTSYQNPGKLSCLELNDCSRLRSLPNMVNLELLKALDLSGCSELETIQGFPRNLKELYLVGTAVRQVPQLPQSLEFFNAHGCVSLKSIRLDFKKLPVHYTFSNCFDLSPQVVNDFLVQAMANVIAKHIPRERHVTGFSQKTVQRSSRDSQQELNKTLAFSFCAPSHANQNSKLDLQPGSSSMTRLDPSWRNTLVGFAMLVQVAFSEGYCDDTDFGISCVCKWKNKEGHSHRREINLHCWALGKAVERDHTFVFFDVNMRPDTDEGNDPDIWADLVVFEFFPVNKQRKPLNDSCTVTRCGVRLITAVNCNTSIENISPVLSLDPMEVSGNEDEEVLRVRYAGLQEIYKALFLYIAGLFNDEDVGLVAPLIANIIDMDVSYGLKVLAYRSLIRVSSNGEIVMHYLLRQMGKEILHTESKKTDKLVDNIQSSMIATKEIEITRSKSRRKNNKEKRVVCVVDRGSRSSDLWVWRKYGQKPIKSSPYPRSYYRCASSKGCFARKQVERSRTDPNVSVITYISEHNHPFPTLRNTLAGSTRSSSSKCSDVTTSASSTVSQDKEGPDKSHLPSSPASPPYAAMVVKEEDMEQWDNMEFDVDVEEDTFIPELFPEDTFADMDKLEENSQTMFLSRRSSGGNMEAQGKNSSDDREVNLPSKILNR |
| >AtWRKY22 |
| MADDWDLHAVVRGCSAVSSSATTTVYSPGVSSHTNPIFTVGRQSNAVSFGEIRDLYTPFTQESVVSSFSCINYPEEPRKPQNQKRPLSLSASSGSVTSKPSGSNTSRSKRRKIQHKKVCHVAAEALNSDVWAWRKYGQKPIKGSPYPRGYYRCSTSKGCLARKQVERNRSDPKMFIVTYTAEHNHPAPTHRNSLAGSTRQKPSDQQTSKSPTTTIATYSSSPVTSADEFVLPVEDHLAVGDLDGEEDLLSLSDTVVSDDFFDGLEEFAAGDSFSGNSAPASFDLSWVVNSAATTTGGI |
| >AtWRKY27 |
| MSSEDWDLFAVVRSCSSSVSTTNSCAGHEDDIGNCKQQQDPPPPPLFQASSSCNELQDSCKPFLPVTTTTTTTWSPPPLLPPPKASSPSPNILLKQEQVLLESQDQKPPLSVRVFPPSTSSSVFVFRGQRDQLLQQQSQPPLRSRKRKNQQKRTICHVTQENLSSDLWAWRKYGQKPIKGSPYPRNYYRCSSSKGCLARKQVERSNLDPNIFIVTYTGEHTHPRPTHRNSLAGSTRNKSQPVNPVPKPDTSPLSDTVKEEIHLSPTTPLKGNDDVQETNGDEDMVGQEVNMEEEEEEEEVEEDDEEEEDDDDVDDLLIPNLAVRDRDDLFFAGSFPSWSAGSAGDGGG |
| >AtWRKY29 |
| MDEGDLEAIVRGYSGSGDAFSGESSGTFSPSFCLPMETSSFYEPEMETSGLDELGELYKPFYPFSTQTILTSSVSLPEDSKPFRDDKKQRSHGCLLSNGSRADHIRISESKSKKSKKNQQKRVVEQVKEENLLSDAWAWRKYGQKPIKGSPYPRSYYRCSSSKGCLARKQVERNPQNPEKFTITYTNEHNHELPTRRNSLAGSTRAKTSQPKPTLTKKSEKEVVSSPTSNPMIPSADESSVAVQEMSVAETSTHQAAGAIEGRRLSNGLPSDLMSGSGTFPSFTGDFDELLNSQEFFSGYLWNY |
| >AtWRKY35 |
| MDNFQGDLTDVVRGIGSGHVSPSPGPPEGPSPSSMSPPPTSDLHVEFPSAATSASCLANPFGDPFVSMKDPLIHLPASYISGAGDNKSNKSFAIFPKIFEDDHIKSQCSVFPRIKISQSNNIHDASTCNSPAITVSSAAVAASPWGMINVNTTNSPRNCLLVDNNNNTSSCSQVQISSSPRNLGIKRRKSQAKKVVCIPAPAAMNSRSSGEVVPSDLWAWRKYGQKPIKGSPYPRGYYRCSSSKGCSARKQVERSRTDPNMLVITYTSEHNHPWPTQRNALAGSTRSSSSSSLNPSSKSSTAAATTSPSSRVFQNNSSKDEPNNSNLPSSSTHPPFDAAAIKEENVEERQEKMEFDYNDVENTYRPELLQEFQHQPEDFFADLDELEGDSLTMLLSHSSGGGNMENKTTIPDVFSDFFDDDESSRSL |
| >AtWRKY65 |
| MKRGLDMARSYNDHESSQETGPESPNSSTFNGMKALISSHSPKRSRRSVEKRVVNVPMKEMEGSRHKGDTTPPSDSWAWRKYGQKPIKGSPYPRGYYRCSSTKGCPARKQVERSRDDPTMILITYTSEHNHPWPLTSSTRNGPKPKPEPKPEPEPEVEPEAEEEDNKFMVLGRGIETTPSCVDEFAWFTEMETTSSTILESPIFSSEKKTAVSGADDVAVFFPMGEEDESLFADLGELPECSVVFRHRSSVVGSQVEIF |
| >AtWRKY69 |
| MHRRAAIQESDDEEDETYNDVVPESPSSCEDSKISKPTPKKSRRNVEKRVVSVPIADVEGSKSRGEVYPPSDSWAWRKYGQKPIKGSPYPRGYYRCSSSKGCPARKQVERSRVDPSKLMITYACDHNHPFPSSSANTKSHHRSSVVLKTAKKEEEYEEEEEELTVTAAEEPPAGLDLSHVDSPLLLGGCYSEIGEFGWFYDASISSSSGSSNFLDVTLERGFSVGQEEDESLFGDLGDLPDCASVFRRGTVATEEQHRRCDFGAIPFCDSSR |
| >AtWRKY30 |
| MEKNHSSGEWEKMKNEINELMIEGRDYAHQFGSASSQETREHLAKKILQSYHKSLTIMNYSGELDQVSQGGGSPKSDDSDQEPLVIKSSKKSMPRWSSKVRIAPGAGVDRTLDDGFSWRKYGQKDILGAKFPRGYYRCTYRKSQGCEATKQVQRSDENQMLLEISYRGIHSCSQAANVGTTMPIQNLEPNQTQEHGNLDMVKESVDNYNHQAHLHHNLHYPLSSTPNLENNNAYMLQMRDQNIEYFGSTSFSSDLGTSINYNFPASGSASHSASNSPSTVPLESPFESYDPNHPYGGFGGFYS |
| >AtWRKY38 |
| MEMNSPHEKAVQAIRYGHSCAMRLKRRLNHPMADGGPLSSYDLAKSIVESFSNAISILSAKPETEDDQFSDLSSRDSSPPPQGSPSKKRKIDSTNSSENWRDDSPDPIYYDGYLWRKYGQKSIKKSNHQRSYYRCSYNKDHNCEARKHEQKIKDNPPVYRTTYFGHHTCKTEHNLDAIFIAGQDPLDDFKSTQMIRFGKDQDQEKESRSNGFSLSVKHEEDIIKEQAIDQYREITSNDQDCQDVIEEYLSSPSGSYPPSSSSGSESADFNSDLLFDNPDSWDRYDQFYF |
| >AtWRKY41 |
| MEMMNWERRSLLNELIHGLKAAKQLQGSSSPSLSASSSYLTTEIKENLLHNIVSSFKKAILMLNGSTTQHNPTIELAPDPLAHPGKVPGSPASITGNPRSEEFFNVRSKEFNLSSKKRKMLPKWTEQVRISPERGLEGPHDDIFSWRKYGQKDILGAKFPRSYYRCTFRNTQYCWATKQVQRSDGDPTIFEVTYRGTHTCSQGIPLPEKRETKPKHTVAVNYQNLRASLTVRTGGLGSEAFSFPVTSPLYTYESINGGGTFYHHVGSSGPSDFTGLISTNTSTGSSPIFDVNFQFDPTAEINTGFPTFFHNSI |
| >AtWRKY46 |
| MMMEEKLVINELELGKELANRLMNNLKHTSSVDSNKTLISDILRIYQNAIFMLSFNQDKNILKRSLEIDGKDSKNVFKKRKVSEKNTEKVKVFVATEQENGSIDDGHCWRKYGQKEIHGSKNPRAYYRCTHRFTQDCLAVKQVQKSDTDPSLFEVKYLGNHTCNNITSPKTTTNFSVSLTNTNIFEGNRVHVTEQSEDMKPTKSEEVMISLEDLENKKNIFRTFSFSNHEIENGVWKSNLFLGNFVEDLSPATSGSAITSEVLSAPAAVENSETADSYFSSLDNIIDFGQDWLWS |
| >AtWRKY52 |
| MGFDICYIDVGCRKKKHSESRVKKVVSIPAIDEGDLWTWRKYGQKDILGSRFPRGYYRCAYKFTHGCKATKQVQRSETDSNMLAITYLSEHNHPRPTKRKALADSTRSTSSSIC |
| >AtWRKY53 |
| MEGRDMLSWEQKTLLSELINGFDAAKKLQARLREAPSPSSSFSSPATAVAETNEILVKQIVSSYERSLLLLNWSSSPSVQLIPTPVTVVPVANPGSVPESPASINGSPRSEEFADGGGSSESHHRQDYIFNSKKRKMLPKWSEKVRISPERGLEGPQDDVFSWRKYGQKDILGAKFPRSYYRCTHRSTQNCWATKQVQRSDGDATVFEVTYRGTHTCSQAITRTPPLASPEKRQDTRVKPAITQKPKDILESLKSNLTVRTDGLDDGKDVFSFPDTPPFYNYGTINGEFGHVESSPIFDVVDWFNPTVEIDTTFPAFLHESIYY |
| >AtWRKY54 |
| MDSNSNNTKSIKRKVVDQLVEGYEFATQLQLLLSHQHSNQYHIDETRLVSGSGSVSGGPDPVDELMSKILGSFHKTISVLDSFDPVAVSVPIAVEGSWNASCGDDSATPVSCNGGDSGESKKKRLGVGKGKRGCYTRKTRSHTRIVEAKSSEDRYAWRKYGQKEILNTTFPRSYFRCTHKPTQGCKATKQVQKQDQDSEMFQITYIGYHTCTANDQTHAKTEPFDQEIIMDSEKTLAASTAQNHVNAMVQEQENNTSSVTAIDAGMVKEEQNNNGDQSKDYYEGSSTGEDLSLVWQETMMFDDHQNHYYCGETSTTSHQFGFIDNDDQFSSFFDSYCADYERTSAM |
| >AtWRKY55 |
| MYSYKKISYQMEEVMSMIFHGMKLVKSLESSLPEKPPESLLTSLDEIVKTFSDANERLKMLLEIKNSETALNKTKPVIVSVANQMLMQMEPGLMQEYWLRYGGSTSSQGTEAMFQTQLMAVDGGGERNLTAAVERSGASGSSTPRQRRRKDEGEEQTVLVAALRTGNTDLPPDDNHTWRKYGQKEILGSRFPRAYYRCTHQKLYNCPAKKQVQRLNDDPFTFRVTYRGSHTCYNSTAPTASSATPSTIPISSVTTGHSVDYGLAVVDMADVMFGSGGVGTNMDFIFPKNDPS |
| >*AtWRKY62* |
| MNSCQQKAMEKLLHGHGCANQLLIMDQTESDSSMEREDLAKSVLHCFSDALSILIDTNDHQDDQSNNSSPQDSSPVLESSRKPLHKRGRKTSMAESSDYHRHESSTPIYHDGFLWRKYGQKQIKESEYQRSYYKCAYTKDQNCEAKKQVQKIQHNPPLYSTTYFGQHICQLHQAYATFPIDTSDFEEHEGSHMIRFGHPNISFSSSTSNLRQHQNHQDRIKDEYMKPVIAEDWSPSQWMSSEVALAVEAFEFNPFWTSHDLSS |
| >*AtWRKY63* |
| MFSNIDHKAVAALLHGQGCANILKTVLDNCKVSSVSTEPLINTILDSFSLALSSVNSPNRQPHHESSSRDMAGLVPQRSSKKKICGVKGLEIYRDDSPNPRLDDGFTWRKYGQKTIKTSLYQRCYYRCAYAKDQNCYATKRVQMIQDSPPVYRTTYLGQHTCKAFGVHDNTYGSEMINFDQVVSESVMRQLATIGEQAVLMEDEANHIMNQEYDINDYLVDDEVFWGNEFPLFSSEDLMLF |
| >*AtWRKY64* |
| MFSNIDQTAVAALLRGQGCANSLKRLLENHKLSSDSTEPLIYTILNSFSLALSFVDPPSLLPHNESSLQNMTSHVLQRSSKKKYYGAEDLEYYRDESPTPRPDDGFTWRKYGQKTIKTSPYQRCYYRCTYAKDQNCNARKRVQMIQDNPPVYRTTYLGKHVCKAVAVHDDTYGSEMIKFDQVVSESVMPQLATIDEQAITMEDEAIDHIMNQECDINDFSVDDDPFWASQFPPFSSEDIMFFDNIANLD |
| >*AtWRKY66* |
| MSLEIDAKAVSALLLGQGCANNLKTLLKNHETGSVSTEPLINSILDSFSFALSSQNIPRHVSQRSSKKKMCGIQGMEDSPTPAHIDGFIWRKYGQKTIKTSPHQRWYYRCAYAKDQNCDATKRVQKIQDNPPVYRNTYVGQHACEAPAYAVNNGGTYGSKMIKFDYVIPESVMPQPLSIDSQEITMEDKDTDDHILNYINEHLMEDEAYDVFPDVLGERCCFGLEPFPGLNINKS |
| >*AtWRKY67* |
| MVSNIDHKAMEALLRGQGCANNLKILLENGEISSVSTEPLIHTILDSFSLALSFMDSPNHPPYHESSSHNMASHMSRRSSKQVQHRRKLCVAEGLVNYNHDSRTMCPNDGFTWRKYGQKTIKASAHKRCYYRCTYAKDQNCNATKRVQKIKDNPPVYRTTYLGKHVCKAFAVHDDTYSSTMIRFDQVVPEPIMPQLTTIDHQVITVEENSAEHIMNQECDINDYLVDDDPFWASQFPPFPSSDTMFLENISAFD |
| >*AtWRKY70* |
| MDTNKAKKLKVMNQLVEGHDLTTQLQQLLSQPGSGLEDLVAKILVCFNNTISVLDTFEPISSSSSLAAVEGSQNASCDNDGKFEDSGDSRKRLGPVKGKRGCYKRKKRSETCTIESTILEDAFSWRKYGQKEILNAKFPRSYFRCTHKYTQGCKATKQVQKVELEPKMFSITYIGNHTCNTNAETPKSKTCDHHDEIFMDSEDHKSPSLSTSMKEEDNPHRHHGSSTENDLSLVWPEMVFEEDYHHQASYVNGKTSTSIDVLGSQDLMVFGGGGDFEFSENEHFSIFSSCSNLS |

Table S19 Segmentally duplicated *SmWRKY* gene pairs

| **Chr** | **Label** | **Chr** | **Label** | **Duplication Type** |
| --- | --- | --- | --- | --- |
| GWHAOSJ00000006 | *SmWRKY109* | GWHAOSJ00000013 | *SmWRKY75* | Segmental duplication |
| GWHAOSJ00000006 | *SmWRKY97* | GWHAOSJ00000013 | *SmWRKY52* | Segmental duplication |
| GWHAOSJ00000006 | *SmWRKY57* | GWHAOSJ00000023 | *SmWRKY99* | Segmental duplication |
| GWHAOSJ00000006 | *SmWRKY109* | GWHAOSJ00000023 | *SmWRKY110* | Segmental duplication |
| GWHAOSJ00000006 | *SmWRKY72A* | GWHAOSJ00000040 | *SmWRKY102* | Segmental duplication |
| GWHAOSJ00000010 | *SmWRKY83* | GWHAOSJ00000013 | *SmWRKY27* | Segmental duplication |
| GWHAOSJ00000010 | *SmWRKY53* | GWHAOSJ00000013 | *SmWRKY30* | Segmental duplication |
| GWHAOSJ00000010 | *SmWRKY23* | GWHAOSJ00000023 | *SmWRKY48* | Segmental duplication |
| GWHAOSJ00000010 | *SmWRKY55* | GWHAOSJ00000084 | *SmWRKY106* | Segmental duplication |
| GWHAOSJ00000010 | *SmWRKY83* | GWHAOSJ00000084 | *SmWRKY84* | Segmental duplication |
| GWHAOSJ00000010 | *SmWRKY53* | GWHAOSJ00000084 | *SmWRKY46* | Segmental duplication |
| GWHAOSJ00000013 | *SmWRKY80* | GWHAOSJ00000020 | *SmWRKY21* | Segmental duplication |
| GWHAOSJ00000013 | *SmWRKY76* | GWHAOSJ00000023 | *SmWRKY11* | Segmental duplication |
| GWHAOSJ00000013 | *SmWRKY41* | GWHAOSJ00000023 | *SmWRKY96* | Segmental duplication |
| GWHAOSJ00000013 | *SmWRKY93* | GWHAOSJ00000023 | *SmWRKY95* | Segmental duplication |
| GWHAOSJ00000013 | *SmWRKY27* | GWHAOSJ00000084 | *SmWRKY84* | Segmental duplication |
| GWHAOSJ00000020 | *SmWRKY7* | GWHAOSJ00000040 | *SmWRKY105* | Segmental duplication |
| GWHAOSJ00000020 | *SmWRKY7* | GWHAOSJ00000057 | *SmWRKY15* | Segmental duplication |
| GWHAOSJ00000020 | *SmWRKY40* | GWHAOSJ00000057 | *SmWRKY94* | Segmental duplication |
| GWHAOSJ00000020 | *SmWRKY50* | GWHAOSJ00000084 | *SmWRKY98* | Segmental duplication |
| GWHAOSJ00000023 | *SmWRKY42* | GWHAOSJ00000057 | *SmWRKY100* | Segmental duplication |
| GWHAOSJ00000023 | *SmWRKY104* | GWHAOSJ00000057 | *SmWRKY103* | Segmental duplication |
| GWHAOSJ00000023 | *SmWRKY90* | GWHAOSJ00000057 | *SmWRKY88* | Segmental duplication |
| GWHAOSJ00000023 | *SmWRKY104* | GWHAOSJ00000057 | *SmWRKY65* | Segmental duplication |
| GWHAOSJ00000023 | *SmWRKY89* | GWHAOSJ00000057 | *SmWRKY28* | Segmental duplication |
| GWHAOSJ00000023 | *SmWRKY48* | GWHAOSJ00000084 | *SmWRKY85* | Segmental duplication |
| GWHAOSJ00000040 | *SmWRKY91* | GWHAOSJ00000040 | *SmWRKY92* | Segmental duplication |
| GWHAOSJ00000040 | *SmWRKY105* | GWHAOSJ00000057 | *SmWRKY15* | Segmental duplication |
| GWHAOSJ00000057 | *SmWRKY6* | GWHAOSJ00000057 | *SmWRKY101* | Segmental duplication |

Table S20 37 *WRKY61* gene in *Salvia* L.

| *WRKY61* gene |
| --- |
| >S_cavaleriei_S0123  MESEYLEVYKSVVSEISKGMEQVKEVKECCSNKEQEGVLERMLSSHQEALLILTGRGKCQSGISYPPDVCGSKDASSRLWKRKTITDIYSVKIETSSSQQDGFAWRKYGEKHILGAKYPRAYYRCVHRLTKYCWATKQVQRSDDGTVFEVTYRARHTC  >S_daiguii_S0297  MESEYVEVYKSVVSEISKGMEQVKEVKERCSNKEQEGVLERMLSSHQEALLILTGRAPQSGISYPTSVCGSKDASSRLWKRKTITDISSVKIQTSSSQQDGFAWRKYGQKHILGAKYPRAYYRCVHRLSKYCWATKQVQRSDDGTVFEVTYRARHTC  >S_liguliloba_S0747  MESEYLEVYKSVVSEISKGMEQVKEVKESCSNKEQEGVLERMLSSHEEALLILTGRGKCDSGISSLPDPPASSTSVRGSKDASSRLWKRKTVKVETSSSQQDGFAWRKYGQKDILGAKYPRAYYRCVHRRSKYCSATKQVQRSDDGTVFEVTYKGRHTC  >S_chienii_S1094  MESEYLEVYKSVVSEISKGMEQVKEVKESCSNKEQEGVLERMLSSHEEALLILTGRGKCDSGISSLPDPPASSTSVRGSKDASSRLWKRKTVKVETSSSQQDGFAWRKYGQKDILGAKYPRAYYRCVHRRSKYCSATKQVQRSDDGTVFEVTYKGRHTC  >S_prionitis_S1097  MESEYLELYKSVVSEISKGLEQVKEVKESCSNKEQEGVLERMLSSHEEALLILTGRAPRGKCHSGISSLPDPPASSTSVSGSKDASSRLWKRKTTTDIYSVKVETSSSQQDGFAWRKYGQKDILGAKYPRAYYRCVHRHSKYCWATKQVQRSDDGTVFEVTYKGRHTC  >S_chinensis_S0171  MESEYLELYKSVVSEISKGMEQVKEVKECCSNKEQEGVLERMLSSHEEALLILTGRAPRGKCDSGISSLPDPPASSTSVRGSKDASSRLWKRKIITDTYSVKVETSSPQQDGFAWRKYGEEDTLGAKYPRAYYICVHKYCGATKHVQRSDDGTVFEVTYNRRHTCQ  >S_hylocharis_S1155  MESEYVELYKSVVSEISKGMEQVKEVKECCSNKEQEGFLERMLSCHEEALLILTGGRAPQPQGQCQSGISSLSDPPDSSTCVRKVTGSKYASSGLWKRKYCKSGKKIVKIEASSSELPLEDGYSWRKYGQKAILNAKYPRGYYRCVHGHSKGCLAKKQVQRSDDGTIFEVTYTGRHTCQGL  >S_flava_S1153  MESEYLELYKSVVSEISKGMEQVKEVKECCSNKEQEGFLERMLSSHEEALLILTGRAPQGQCQSGISSLSDPPAPGSSTSVRKVTGSKDASSGLWKRKYCESRKKIVKIGASSELPLEDGYSWRKFGQKAILGAKYPRGYYRCVHSKKCFARKQVQRSDDGTIFEVTYTGRHTCQGL  >S_cyclostegia_S1171  MESEYLQLYKSVVSEISKGMEQVKEVKESCSNKEQEGFLDRMLSSHEEALLILTGQGQCQSVISSLSDPPAPGSSTSVRKVTGSKDASSGLWKRKYCESRKKIVKIGASSELPLEDGYSWRKFGQKAILGAKYPRGYYRCVHRVSKKCFARKQVQRSDDGTIFEVTYTGRHTCQGL  >S_przewalskii_S1161  MESEYLQLYKSVVSEISKGMEQVKQVKESCSNKEQEGFLDRMLSSHEEALLILTGRAPHGQCQSGISSLSDPPDSSTSLRKVTGSKDASSGLWERKYCKSRKKFVKIEASSELPLEDGYGWRKYGQKTILGAKYPRAYYRCIHQYSKGCLAKNQVQRSDDGAIFEVTYTGRHTCQGL  >S_aerea_S1170  MESEYLQLYKSVVSEISKGMEQVKQVKESCSNKEQEGFLDRMLSSHEEALLILTGRAPHGQCQSGISSLSDPPDSSTSLRKVTGSKDASSGLWERKYCKSRKKFVKIEASSELPLEDGYGWRKYGQKTILGAKYPRAYYRCIHQYSKGCLAKNQVQRSDDGAIFEVTYTGRHTCQGL  >S_digitaloides_S1173  MEREYLQLYKSVVSEISKGMEQVKQVKESCSNKEQEGFLDRMLSSHEEALLILTGRAPQGQCQSGISSLSDPPDSSTSLRKVTGSKDASSGLWERKYCKSRKKFVKIEASSELPLEDGYCWRKYGQKTILGAKYPRAYYRCIHQYSKGCLAKNQVQRSDDGAIFEVTYTGRHTCQGL  >S_mekongensis_S1167  MESEYVELYKSVVSEISKGMEQVKEVKECCSNKEQEGFLDRMLSSHEEALLILAGRAPQGQCQSGISSSLSDPPDSSTSLRKLTGSNDASSGFWKREYCHSRKKIVKIEASSELPLEVEDGYSWRKYGQKTILGAKYPRGYYRCIHQYSKGCVAKKQVQRSDDGTIFEVTYSGQHTCRG  >S_castanea_S1169  MESEYVQLYKSVVSEISKGMEQVKEVKECCSNKEQEGFLDRMLSSHEEALLILTGRAPQGQCQSGISSSLSDPPDSSTSLRKVTGSKDASSGLWKREYRKKIDKIEASSELPLEVEDGYSWRKYGQKAILGAKYPRGYYRCVHRLSKHCFATKQVHRSDDGTIFEVTYIGQHTCQGL  >S_nipponica_S0742  MEGEYLQLYKSLVSEISKGMEQVKQVKESCSNKEQEGVLDRMLSSHEEALLILTGHAPQGQCQSGISSSSDPPDSSTSVPKVTGSKDASIGLGKREYYASRKKIVKIEASGASSELLLEDGYRWTKYGQKRILNAKYPRGYYKCNHSRGCMARKQVQRSDDGTIFEVTYTGRHTCQGL  >S_glutinosa_S1031  MEGEYLQLYKSLVSEISKGMEQVKQVKESCSNKEQEGVLDGMLSSHEEALLILTGRAPQGQCQSGISSLSDPPDSSTSVRKVTGSKDASISLGKRKYCKSRKKIVKIEASGASSELPLEDGYSWRMYGQKTILGAKYPRDYYKCIHKYSRTCSARKQVQRSDDGTIFEVTYTGRHTCPGL  >S_substolonifera_S0477  MESEYLQLYRSVVGEISKGMEQVKQVKECCSNKEQAGVVERILSSHEEALLILTGRAPQGQCQSGISSLSDPPDSSTSVTKVTGSKDASKVLWKRKYCKYEKKIVKIEASSELPLEDGYRWKKYGQKTILNAKYPRGYYRCIQPHGRTCLARKQVQRSDDGTIFEVTYKGPHTCPGL  >S_adiantifolia_S0671  MESEYLELYKSVVSEISKGMEQVKEVKECCSNKEQDGFLERMLSSHEEALLILTGRAPQGQCQSGISSLSDPPDSPTGSKDARSLWKREYFKRRNEIVKIEASSDSELPLEDGYAWRKYGKKTILNAKYPRRYYRCAQSKTCLAKKRVQRMSDDGTVSEVTYRGQHTC  >S_cavaleriei_S0761  MESEYVELYKSVVSEISKGMEQVKEVKECCSNKEQDGFLDRMLSSHEEALLILTGRAPQGQCQSGISSLSDPPDSPTGSKDARSRLWKREYFKRRNEIVKIEASSDSEDGYAWRKYGKKTILNAKYPRRYYKCAQSKTCLAKKRVQRSDDGTVSEVTYGGQHTC  >S_filicifolia_S0940  MESEYVELYKSVVSEISKGMEQVKEVKECCSNKEQDGFLDRMLSSHEEALLILTGRAPQGQCQSGISSLSDPPDSPTGSKDARSRLWKREYFKRRNEIVKIEASSDSELPLEDGYAWRKYGKKTILNAKYPRRYYICAQSKTCLAKKRVQRSDDGTVFEVTYRGQHTC  >S_cavaleriei_S0994  MESEYVELYKSVVSEISKGMEQVKEVKECCSNKEQDGFLDRMLSSHEEALLILTGRAPQGQCQSGISSLSDPPDSPTGSKDARSRLWKREYFKRRNEIVKIEASSDSELPLEDGYAWRKYGKKTILNAKYPRRYYICAQSKTCLAKKRVQRSDDGTVFEVTYRGQHTC  >S_meiliensis_S0271  MESEYLELYKSVVSEISKGMEQVKEVKECCSNKEQDGFLERMLSSHEEALLILTGRAPQGQCQSGISSLSDPPDSPTGSKDARSRLWKREYFKRRNKIFKIEASSDSELAHEDGYAWRKYGKKAILNAKYPRGYYRCIQSKTCLAKKRVQWSDDGTVFEVTYKGQHTCPG  >S_honania_S0356  MESEYLELYKSVVSEISKGMEQVKEVKECCSNKEQDGFLERMLSSHEEALLILTGRAPQGQCQSGISSLSDPPDSPTGSKDARSRLWKREYFKRRNKIVKIEASSDSELAHEDGYAWRKYGKKAILNAKYPRGYYRCIQSKTCLAKKRVQWSDDGTVFEVTYKGQHTCPG  >S_kiangsiensis_S1127  MESEYVELYKSVVSEISKGMEQVKEVKECCSNKEQDGFLDRMLSSHEEALLILTGRAPQGQCQSGISSLSDPPDSPTGSKDARSRLWKKEYFKRRNKIVKIEASSDSELPHEDGYAWRKYGKKAILNAKYPRGYYRCIQSKTCLAKKRVQWSDDGTVFEVAYKGQHTCPG  >S_miltiorrhiza_S0146  MESEYLELYKSVVSEISKGMEQVKECCSNKEQDGFLERMLSSHEEALLILTGRAPQGQCQSGISSLSDPPDSPTGSKDARSRLWKREYFKRRNKIVKIEASSDSELPHEDGYAWRKYGKKAILNAKYPRGYYRCIQSKTCLAKKRVQRSDDGTVFEVTYKGQHTCPGL  >S_miltiorrhiza_S0365  MESEYLELYKSVVSEISKGMEQVKECCSNKEQDGFLERMLSSHEEALLILTGRAPQGQCQSGISSLSDPPDSPTGSKDARSRLWKREYFKRRNKIVKIEASSDSELPHEDGYAWRKYGKKAILNAKYPRGYYRCIQSKTCLAKKRVQRSDDGTVFEVTYKGQHTCPGL  >S_paramiltiorrhiza_S0491  MESEYLELYKSVVSEISKGMEQVKECCSNKEQDGFLERMLSSHEEALLILTGRAPQGQCQSGISSLSDPPDSPTGSKDARSRLWKREYFKRRNKIVKIEASSDSELPLEDGYAWRKYGKKAILNAKYPRRYYKCIQSKTCLAKKRVQRSDDGTVFEVTYKGQHTCPGL  >S_bowleyana_S0603  MESEYLELYKSVVSEISKGMEQVKECCSNKEQDGFLERMLSSHEEALLILTGRAPQGQCQSGISSLSDPPDSPTGSKDARSRLWKREYFKRRNKIVKIEASSDSELPLEDGYAWRKYGKKAILNAKYPRRYYKCIQSKTCLAKKRVQRSDDGTVFEVTYKGQHTCPG  >S_sinica_S0175  MESEYLELYKSVVSEISKGMEQVKECCSNKEQDEFLDRMLSSHEEALLILTGRAPQGQCQSGISSLSDPPDSPTGSKDARSRLWKREYFKRRNKIVKIEASSDSELPLEDGYAWRKYGKKAILNAKYPRRYYKCIQSKTCLAKKRVQWSDDGTVFEVTYKGQHTCPGL  >S_plectranthoides_S0460  MESEYLELYKSVVSEISKGMEQVKECCSNKEQDEFLDRMLSSHEEALLILTGRAPQGQCQSGISSLSDPPDSPTGSKDARSRSWKREYFKRRNKIVKIEASSDSELPLEDGYAWRKYGKKAILNAKYPRRYYKCIQSKTCLAKKRVQRSDDGTVFEVTYKGQHTCPGL  >S_nanchuanensis_S0484  MESEYLELYKSLVSEISKGMEQVKECCSNKEQDGFLDRMLCSHEEALLILTGRGGGQCQSGISSLSYPPDSSTSGSKDARRRSRLWEREYFKRRNKIVKIEASSDSELPLEDGYAWSKYGKKAILNAKYPRRYYRCIQSKTCLAKKRVQRSDDGTVFEVTYGGQHT  >S_yunnanensis_S1151  MESEYLELYKSLVSEISKGMEQVKECCSNKEQDGFLDRMLCSHEEALLILTGRAPQGQCQSGISSLSYPPDSSTSGSKNARRRSHLWLREYFKTRNKIVKIEASSDSELPLEDGYAWRKYGKKAILNAKYPRGYYRCIQSKTCLAKKQVQRSDDGTVFEVTYGGQHT  >S_trijuga_S1164  MESEYLEVYNSVVGEIRKGMEQVKECCSNREQEGVLERMLSSHEEALLILTGRAPQGQCQSGISSLSDPPDASTSVREVTGSKGATSGLWKRKTRTNIYRVKIEASSSQQPLEDGYAWRKYGHKVILNAKYPRSYYRCTSKTCLARKQVQKSDDGTIFEVTYTGHHTCQGL  >*Saspl_022277*  MEAKLDNLLTLRLTLHYLLWLSSSFQESSSRNFQCDRMESERVELLNSLVSEISKGMEQVKQLKDSCFCNSNNGVLDKMLSSHEEALFILTGRNPQQQCHSDSGISSLSDPPATASLKRKSSTGKVSGDSSVPPEDGYSWRRYGRKEILGSKYPREYYRCSLKGSTCCGAKKQIQRSDDGTVFEITYRGLHVCRQSPKS  >S_nanchuanensis_S0517  MESEYLELYKSLVSEISKGMEQVKEVKECCSNKEQDGFLDRMLCSHEEALLILTGRAPQGQCQSGISSLSYPPDSSTSASKDARRRSRLWEREYFKRRNTICQDGILFTTTRGWICLEQVRQESYPCQISKRLLMHSENLFGKTSTEVRRWDCIGHIQRTTYM  >*SiWRKY30*  LIINNDTNNCTKTRSRPIPITSNSETANAFLFFSAYFPFVQNAIAGTRMDKEPPQAFESIQAYFTLKHEVSKGREQVKQLKAASSGDQEKILERMLASYEEVWSILNEMAAEGKDLPVIPPSSDSPASGKSVLRHKRGSYKRKSSDSISVRRHRAPEELDDDGYCWRKYGQKDILGAKYPRSYFRCLYRYTQGCMATKQVQRSDDEPLIYDTTYTGKHSCSSSAPIDKETLVDKSQFQLLISDSTYLINGKLVNKSSHHSKRLLIKSQLYLRSCLGRGYLMCHSNTKFLYYYNHINYLGRGYL  >*AtWRKY41*  MEMMNWERRSLLNELIHGLKAAKQLQGSSSPSLSASSSYLTTEIKENLLHNIVSSFKKAILMLNGSTTQHNPTIELAPDPLAHPGKVPGSPASITGNPRSEEFFNVRSKEFNLSSKKRKMLPKWTEQVRISPERGLEGPHDDIFSWRKYGQKDILGAKFPRSYYRCTFRNTQYCWATKQVQRSDGDPTIFEVTYRGTHTCSQGIPLPEKRETKPKHTVAVNYQNLRASLTVRTGGLGSEAFSFPVTSPLYTYESINGGGTFYHHVGSSGPSDFTGLISTNTSTGSSPIFDVNFQFDPTAEINTGFPTFFHNSI  >*AtWRKY53*  MEGRDMLSWEQKTLLSELINGFDAAKKLQARLREAPSPSSSFSSPATAVAETNEILVKQIVSSYERSLLLLNWSSSPSVQLIPTPVTVVPVANPGSVPESPASINGSPRSEEFADGGGSSESHHRQDYIFNSKKRKMLPKWSEKVRISPERGLEGPQDDVFSWRKYGQKDILGAKFPRSYYRCTHRSTQNCWATKQVQRSDGDATVFEVTYRGTHTCSQAITRTPPLASPEKRQDTRVKPAITQKPKDILESLKSNLTVRTDGLDDGKDVFSFPDTPPFYNYGTINGEFGHVESSPIFDVVDWFNPTVEIDTTFPAFLHESIYY  >*AtWRKY55*  MYSYKKISYQMEEVMSMIFHGMKLVKSLESSLPEKPPESLLTSLDEIVKTFSDANERLKMLLEIKNSETALNKTKPVIVSVANQMLMQMEPGLMQEYWLRYGGSTSSQGTEAMFQTQLMAVDGGGERNLTAAVERSGASGSSTPRQRRRKDEGEEQTVLVAALRTGNTDLPPDDNHTWRKYGQKEILGSRFPRAYYRCTHQKLYNCPAKKQVQRLNDDPFTFRVTYRGSHTCYNSTAPTASSATPSTIPISSVTTGHSVDYGLAVVDMADVMFGSGGVGTNMDFIFPKNDPS |

Table S21 Expression of *WRKY61* and total tanshinone content in *Salvia* L.

| **Gene id** | **WRKY61_fpkm** | **Total tanshinone (mg/g DW)** |
| --- | --- | --- |
| *S_cavaleriei_S0123* | 5.54 | 0 |
| *S_daiguii_S0297* | 26.00666667 | 0 |
| *S_liguliloba_S0747* | 5.46 | 0 |
| *S_chienii_S1094* | 9.1 | 0 |
| *S_chinensis_S0171* | 12.48 | 0 |
| *S_nipponica_S0742* | 2.34 | 0 |
| *S_substolonifera_S0477* | 2.93 | 0 |
| *S_adiantifolia_S0671* | 14.51 | 0 |
| *S_cavaleriei_S0761* | 24.24 | 0 |
| *S_filicifolia_S0940* | 0.04 | 0 |
| *S_cavaleriei_S0994* | 0 | 0 |
| *S_kiangsiensis_S1127* | 1.98 | 0 |
| *S_sinica_S0175* | 4.62 | 0 |
| *S_plectranthoides_S0460* | 44.17 | 0 |
| *S_paramiltiorrhiza_S0491* | 30.79 | 0 |
| *S_bowleyana* | 48.17333333 | 0 |
| *S_nanchuanensis_S0517* | 12.09 | 0 |
| *S_hylocharis_S1155* | 0.07 | 0.004 |
| *S_flava_S1153* | 3.83 | 0.0125 |
| *S_mekongensis_S1167* | 3.5 | 0.04625 |
| *S_glutinosa_S1031* | 0.97 | 0.058416667 |
| *S_nanchuanensis_S0484* | 1.57 | 0.05975 |
| *S_meiliensis_S0271* | 7.55 | 0.09175 |
| *S_cyclostegia_S1171* | 4.18 | 0.134 |
| *S_miltiorrhiza_S0146* | 2.87 | 0.149916667 |
| *S_digitaloides_S1173* | 1.24 | 0.154333333 |
| *S_prionitis_S1097* | 12.92 | 0.1895 |
| *S_yunnanensis_S1151* | 16.44 | 0.2045 |
| *S_aerea_S1170* | 8.02 | 0.209333333 |
| *S_castanea_S1169* | 34.29666667 | 0.286666667 |
| *S_honania_S0356* | 8.46 | 0.311916667 |
| *S_miltiorrhiza_S0365* | 65.64 | 0.358416667 |
| *S_przewalskii_S1161* | 25.55 | 0.864333333 |
| *S_trijuga_S1164* | 7.37 | 0.94925 |

Table S22 aBSREL testing selected branches for selection

| Branch | Rates | Max.dN/dS | TestLRT | Uncorrectedp-value |
| --- | --- | --- | --- | --- |
| Node28 | 2 | >1000(2.75%) | 16.17 | 0.0001 |
| S_NIPPONICA_S0742 | 2 | 68.49(12.41%) | 12.49 | 0.00066 |
| Node10 | 2 | 238.30(2.18%) | 11.32 | 0.0012 |
| Node29 | 2 | 15.07(20.17%) | 10.04 | 0.00228 |
| Node13 | 2 | >1000(1.29%) | 9.69 | 0.00273 |
| S_SUBSTOLONIFERA_S0477 | 2 | 8.25(16.84%) | 9.06 | 0.00376 |
| S_MEKONGENSIS_S1167 | 2 | >1000(12.84%) | 8.56 | 0.00485 |
| S_NANCHUANENSIS_S0484 | 2 | >1000(1.24%) | 7.57 | 0.00799 |
| S_YUNNANENSIS_S1151 | 2 | 340.25(0.73%) | 5.97 | 0.01807 |
| Node8 | 1 | >1000(100.00%) | 5.77 | 0.02001 |
| Node11 | 2 | >1000(0.67%) | 5.39 | 0.02429 |
| Node34 | 1 | >1000(100.00%) | 5.23 | 0.02642 |
| Node19 | 1 | >1000(100.00%) | 4.37 | 0.0411 |
| S_GLUTINOSA_S1031 | 1 | >1000(100.00%) | 4.33 | 0.04186 |
| S_KIANGSIENSIS_S1127 | 1 | >1000(100.00%) | 2.98 | 0.08452 |
| Node44 | 1 | >1000(100.00%) | 2.38 | 0.11583 |
| S_DAIGUII_S0297 | 1 | >1000(100.00%) | 2.38 | 0.11583 |
| S_CHINENSIS_S0171 | 1 | 3.76(100.00%) | 2.31 | 0.12026 |
| Node9 | 1 | >1000(100.00%) | 2.21 | 0.12709 |
| Node18 | 1 | >1000(100.00%) | 1.87 | 0.1524 |
| Node12 | 1 | >1000(100.00%) | 1.62 | 0.17447 |
| Node57 | 1 | >1000(100.00%) | 1.39 | 0.19772 |
| S_CASTANEA_S1169 | 1 | 2.95(100.00%) | 1.37 | 0.20006 |
| S_CAVALERIEI_S0761 | 1 | >1000(100.00%) | 1.3 | 0.20699 |
| Node6 | 1 | >1000(100.00%) | 1.07 | 0.23599 |
| Node5 | 1 | >1000(100.00%) | 1.06 | 0.23678 |
| Node53 | 1 | >1000(100.00%) | 1.06 | 0.23725 |
| S_DIGITALOIDES_S1173 | 1 | >1000(100.00%) | 0.98 | 0.24855 |
| S_CAVALERIEI_S0994 | 1 | >1000(100.00%) | 0.78 | 0.27825 |
| S_MEILIENSIS_S0271 | 1 | >1000(100.00%) | 0.77 | 0.27952 |
| S_AEREA_S1170 | 1 | >1000(100.00%) | 0.63 | 0.30433 |
| Node24 | 1 | 2.22(100.00%) | 0.58 | 0.31388 |
| Node49 | 1 | >1000(100.00%) | 0.53 | 0.32356 |
| S_SINICA_S0175 | 1 | >1000(100.00%) | 0.53 | 0.32428 |
| S_PLECTRANTHOIDES_S0460 | 1 | >1000(100.00%) | 0.51 | 0.32775 |
| Node7 | 1 | >1000(100.00%) | 0.44 | 0.34178 |
| Node41 | 1 | 1.90(100.00%) | 0.38 | 0.35504 |
| Node37 | 1 | 1.32(100.00%) | 0.06 | 0.45421 |
| S_TRIJUGA_S1164 | 1 | 1.15(100.00%) | 0.04 | 0.46095 |
| S_PRIONITIS_S1097 | 1 | 1.22(100.00%) | 0.03 | 0.47112 |
| Node1 | 1 | 0.00(100.00%) | 0 | 1 |
| Node45 | 1 | 0.25(100.00%) | 0 | 1 |
| S_FLAVA_S1153 | 1 | 0.60(100.00%) | 0 | 1 |
| S_CHIENII_S1094 | 1 | 0.00(100.00%) | 0 | 1 |
| S_CAVALERIEI_S0123 | 1 | 0.94(100.00%) | 0 | 1 |
| S_CYCLOSTEGIA_S1171 | 1 | 0.81(100.00%) | 0 | 1 |
| S_HYLOCHARIS_S1155 | 1 | 0.93(100.00%) | 0 | 1 |
| S_ADIANTIFOLIA_S0671 | 1 | 0.64(100.00%) | 0 | 1 |
| S_PARAMILTIORRHIZA_S0491 | 1 | 0.00(100.00%) | 0 | 1 |
| Node23 | 1 | 1.19(100.00%) | -0.01 | 0.5 |

# Table S23 Fast Unconstrained Bayesian AppRoximation

| **Site** | **Partition** | **&alpha** | **&beta** | **&beta-&alpha** | **Prob[&alpha;>&beta;]** | **Prob[&alpha;<&beta;]** | **BayesFactor[&alpha;<&beta;]** |
| --- | --- | --- | --- | --- | --- | --- | --- |
| 1 | 1 | 3.264 | 0.298 | -2.966 | 0.704 | 0.238 | 0.315 |
| 2 | 1 | 0.53 | 0.361 | -0.169 | 0.568 | 0.36 | 0.569 |
| 3 | 1 | 0.545 | 0.698 | 0.152 | 0.3 | 0.639 | 1.791 |
| 4 | 1 | 1.052 | 0.344 | -0.708 | 0.802 | 0.156 | 0.186 |
| 5 | 1 | 0.721 | 0.423 | -0.298 | 0.561 | 0.372 | 0.6 |
| 6 | 1 | 0.533 | 3.882 | 3.35 | 0.003 | 0.981 | 51.895 |
| 7 | 1 | 0.525 | 1.996 | 1.47 | 0.062 | 0.904 | 9.507 |
| 8 | 1 | 0.659 | 1.368 | 0.709 | 0.237 | 0.699 | 2.349 |
| 9 | 1 | 0.567 | 0.422 | -0.145 | 0.538 | 0.393 | 0.654 |
| 10 | 1 | 3.092 | 1.183 | -1.909 | 0.599 | 0.317 | 0.469 |
| 11 | 1 | 0.47 | 0.489 | 0.019 | 0.464 | 0.466 | 0.883 |
| 12 | 1 | 0.424 | 1.024 | 0.6 | 0.16 | 0.795 | 3.918 |
| 13 | 1 | 0.497 | 0.364 | -0.133 | 0.554 | 0.373 | 0.601 |
| 14 | 1 | 0.688 | 0.775 | 0.086 | 0.303 | 0.637 | 1.775 |
| 15 | 1 | 0.497 | 0.338 | -0.159 | 0.574 | 0.352 | 0.549 |
| 16 | 1 | 0.482 | 0.328 | -0.154 | 0.576 | 0.349 | 0.542 |
| 17 | 1 | 0.594 | 0.52 | -0.073 | 0.441 | 0.494 | 0.985 |
| 18 | 1 | 0.53 | 0.383 | -0.147 | 0.552 | 0.377 | 0.612 |
| 19 | 1 | 0.414 | 0.309 | -0.105 | 0.549 | 0.368 | 0.588 |
| 20 | 1 | 2.765 | 0.561 | -2.204 | 0.504 | 0.438 | 0.789 |
| 21 | 1 | 0.497 | 0.338 | -0.159 | 0.574 | 0.352 | 0.549 |
| 22 | 1 | 0.542 | 0.358 | -0.184 | 0.574 | 0.355 | 0.556 |
| 23 | 1 | 0.437 | 0.39 | -0.047 | 0.501 | 0.423 | 0.742 |
| 24 | 1 | 1.649 | 0.418 | -1.231 | 0.782 | 0.174 | 0.214 |
| 25 | 1 | 0.524 | 0.998 | 0.474 | 0.21 | 0.737 | 2.825 |
| 26 | 1 | 0.415 | 0.363 | -0.052 | 0.505 | 0.416 | 0.719 |
| 27 | 1 | 0.53 | 0.383 | -0.147 | 0.552 | 0.377 | 0.612 |
| 28 | 1 | 1.049 | 0.345 | -0.704 | 0.801 | 0.156 | 0.187 |
| 29 | 1 | 0.694 | 3.792 | 3.098 | 0.015 | 0.953 | 20.375 |
| 30 | 1 | 18.875 | 0.336 | -18.539 | 0.988 | 0.009 | 0.009 |
| 31 | 1 | 0.393 | 0.363 | -0.03 | 0.488 | 0.431 | 0.765 |
| 32 | 1 | 0.563 | 0.308 | -0.255 | 0.622 | 0.306 | 0.446 |
| 33 | 1 | 0.504 | 0.659 | 0.156 | 0.33 | 0.608 | 1.57 |
| 34 | 1 | 0.497 | 0.338 | -0.159 | 0.574 | 0.352 | 0.549 |
| 35 | 1 | 0.53 | 0.352 | -0.177 | 0.575 | 0.353 | 0.552 |
| 36 | 1 | 0.53 | 0.886 | 0.357 | 0.234 | 0.71 | 2.479 |
| 37 | 1 | 0.473 | 0.603 | 0.13 | 0.34 | 0.597 | 1.5 |
| 38 | 1 | 1.427 | 1.062 | -0.365 | 0.446 | 0.47 | 0.896 |
| 39 | 1 | 0.471 | 0.758 | 0.287 | 0.28 | 0.662 | 1.979 |
| 40 | 1 | 0.59 | 3.816 | 3.226 | 0.005 | 0.973 | 35.886 |
| 41 | 1 | 0.503 | 0.615 | 0.112 | 0.35 | 0.588 | 1.44 |
| 42 | 1 | 4.601 | 0.563 | -4.038 | 0.539 | 0.407 | 0.695 |
| 43 | 1 | 0.461 | 0.408 | -0.053 | 0.504 | 0.423 | 0.74 |
| 44 | 1 | 0.51 | 0.666 | 0.156 | 0.331 | 0.608 | 1.565 |
| 45 | 1 | 2.677 | 0.682 | -1.996 | 0.851 | 0.102 | 0.114 |
| 46 | 1 | 0.716 | 0.309 | -0.408 | 0.643 | 0.289 | 0.411 |
| 47 | 1 | 0.538 | 0.661 | 0.122 | 0.344 | 0.594 | 1.48 |
| 48 | 1 | 0.497 | 0.338 | -0.159 | 0.574 | 0.352 | 0.549 |
| 49 | 1 | 0.496 | 0.322 | -0.174 | 0.588 | 0.338 | 0.515 |
| 50 | 1 | 0.408 | 0.376 | -0.032 | 0.49 | 0.431 | 0.766 |
| 51 | 1 | 0.461 | 0.408 | -0.053 | 0.504 | 0.423 | 0.74 |
| 52 | 1 | 0.554 | 0.372 | -0.183 | 0.569 | 0.361 | 0.571 |
| 53 | 1 | 0.496 | 0.337 | -0.16 | 0.575 | 0.351 | 0.546 |
| 54 | 1 | 3.284 | 0.629 | -2.655 | 0.48 | 0.464 | 0.875 |
| 55 | 1 | 0.715 | 1.691 | 0.976 | 0.453 | 0.494 | 0.987 |
| 56 | 1 | 0.414 | 0.309 | -0.105 | 0.549 | 0.368 | 0.588 |
| 57 | 1 | 1.069 | 0.556 | -0.513 | 0.651 | 0.285 | 0.403 |
| 58 | 1 | 0.505 | 0.331 | -0.174 | 0.584 | 0.342 | 0.526 |
| 59 | 1 | 3.413 | 3.477 | 0.064 | 0.447 | 0.497 | 1 |
| 60 | 1 | 3.413 | 3.477 | 0.064 | 0.447 | 0.497 | 1 |
| 61 | 1 | 0.972 | 0.58 | -0.391 | 0.618 | 0.315 | 0.466 |
| 62 | 1 | 0.517 | 1.989 | 1.472 | 0.06 | 0.906 | 9.79 |
| 63 | 1 | 0.468 | 0.329 | -0.139 | 0.567 | 0.356 | 0.56 |
| 64 | 1 | 0.541 | 0.669 | 0.128 | 0.341 | 0.597 | 1.498 |
| 65 | 1 | 0.718 | 0.337 | -0.38 | 0.62 | 0.312 | 0.459 |
| 66 | 1 | 0.559 | 1.784 | 1.224 | 0.082 | 0.877 | 7.184 |
| 67 | 1 | 1.895 | 0.495 | -1.4 | 0.833 | 0.126 | 0.146 |
| 68 | 1 | 0.42 | 0.578 | 0.159 | 0.317 | 0.621 | 1.654 |
| 69 | 1 | 0.619 | 0.346 | -0.273 | 0.602 | 0.329 | 0.495 |
| 70 | 1 | 0.47 | 0.489 | 0.019 | 0.464 | 0.466 | 0.883 |
| 71 | 1 | 1.627 | 0.489 | -1.138 | 0.555 | 0.384 | 0.629 |
| 72 | 1 | 1.289 | 1.87 | 0.582 | 0.609 | 0.336 | 0.512 |
| 73 | 1 | 0.5 | 0.979 | 0.479 | 0.249 | 0.697 | 2.322 |
| 74 | 1 | 0.485 | 0.914 | 0.429 | 0.253 | 0.692 | 2.271 |
| 75 | 1 | 0.737 | 0.63 | -0.107 | 0.399 | 0.539 | 1.181 |
| 76 | 1 | 1.02 | 0.307 | -0.714 | 0.828 | 0.133 | 0.155 |
| 77 | 1 | 0.394 | 0.301 | -0.093 | 0.541 | 0.373 | 0.602 |
| 78 | 1 | 2.145 | 1.872 | -0.274 | 0.454 | 0.489 | 0.966 |
| 79 | 1 | 2.687 | 1.743 | -0.944 | 0.468 | 0.475 | 0.915 |
| 80 | 1 | 0.956 | 0.952 | -0.004 | 0.338 | 0.599 | 1.508 |
| 81 | 1 | 0.515 | 1.038 | 0.523 | 0.199 | 0.748 | 3.007 |
| 82 | 1 | 0.488 | 1.728 | 1.24 | 0.118 | 0.843 | 5.423 |
| 83 | 1 | 0.513 | 0.355 | -0.158 | 0.567 | 0.36 | 0.569 |
| 84 | 1 | 0.55 | 1.161 | 0.611 | 0.192 | 0.756 | 3.127 |
| 85 | 1 | 0.505 | 1.272 | 0.766 | 0.164 | 0.788 | 3.759 |
| 86 | 1 | 0.641 | 3.22 | 2.579 | 0.023 | 0.946 | 17.591 |
| 87 | 1 | 0.612 | 0.882 | 0.27 | 0.3 | 0.641 | 1.805 |
| 88 | 1 | 0.6 | 0.906 | 0.306 | 0.295 | 0.647 | 1.85 |
| 89 | 1 | 0.522 | 0.674 | 0.152 | 0.333 | 0.605 | 1.55 |
| 90 | 1 | 0.466 | 0.327 | -0.139 | 0.568 | 0.355 | 0.556 |
| 91 | 1 | 0.393 | 0.363 | -0.03 | 0.488 | 0.431 | 0.765 |
| 92 | 1 | 0.547 | 0.703 | 0.155 | 0.331 | 0.608 | 1.569 |
| 93 | 1 | 0.719 | 1.296 | 0.577 | 0.177 | 0.768 | 3.35 |
| 94 | 1 | 2.066 | 0.324 | -1.743 | 0.668 | 0.27 | 0.374 |
| 95 | 1 | 2.267 | 1.311 | -0.956 | 0.475 | 0.467 | 0.886 |
| 96 | 1 | 0.527 | 0.72 | 0.193 | 0.432 | 0.504 | 1.026 |
| 97 | 1 | 0.646 | 0.793 | 0.147 | 0.29 | 0.65 | 1.879 |
| 98 | 1 | 0.656 | 0.895 | 0.239 | 0.248 | 0.694 | 2.288 |
| 99 | 1 | 0.534 | 2.218 | 1.684 | 0.039 | 0.93 | 13.509 |
| 100 | 1 | 0.477 | 1.208 | 0.731 | 0.16 | 0.794 | 3.896 |
| 101 | 1 | 1.217 | 0.67 | -0.547 | 0.408 | 0.531 | 1.145 |
| 102 | 1 | 0.569 | 2.641 | 2.072 | 0.04 | 0.928 | 13.009 |
| 103 | 1 | 0.478 | 0.563 | 0.085 | 0.365 | 0.571 | 1.343 |
| 104 | 1 | 0.565 | 2.533 | 1.968 | 0.043 | 0.925 | 12.379 |
| 105 | 1 | 1.499 | 2.103 | 0.603 | 0.238 | 0.656 | 1.925 |
| 106 | 1 | 8.257 | 4.76 | -3.497 | 0.443 | 0.353 | 0.552 |
| 107 | 1 | 1.085 | 4.641 | 3.556 | 0.026 | 0.902 | 9.297 |
| 108 | 1 | 8.665 | 3.306 | -5.359 | 0.561 | 0.262 | 0.359 |
| 109 | 1 | 0.682 | 3.442 | 2.76 | 0.021 | 0.943 | 16.651 |
| 110 | 1 | 0.605 | 1.727 | 1.122 | 0.114 | 0.84 | 5.307 |
| 111 | 1 | 1.545 | 1.402 | -0.144 | 0.381 | 0.546 | 1.216 |
| 112 | 1 | 0.568 | 0.821 | 0.254 | 0.304 | 0.637 | 1.772 |
| 113 | 1 | 0.403 | 0.974 | 0.571 | 0.156 | 0.8 | 4.038 |
| 114 | 1 | 0.497 | 0.357 | -0.14 | 0.559 | 0.368 | 0.588 |
| 115 | 1 | 1.397 | 1.045 | -0.352 | 0.448 | 0.469 | 0.891 |
| 116 | 1 | 1.069 | 0.898 | -0.171 | 0.449 | 0.471 | 0.899 |
| 117 | 1 | 1.162 | 0.666 | -0.497 | 0.494 | 0.444 | 0.806 |
| 118 | 1 | 1.874 | 1.177 | -0.696 | 0.473 | 0.468 | 0.89 |
| 119 | 1 | 1.89 | 1.124 | -0.766 | 0.477 | 0.464 | 0.875 |
| 120 | 1 | 0.477 | 0.851 | 0.375 | 0.219 | 0.727 | 2.686 |
| 121 | 1 | 0.752 | 0.398 | -0.354 | 0.721 | 0.226 | 0.295 |
| 122 | 1 | 1.142 | 0.471 | -0.672 | 0.73 | 0.219 | 0.284 |
| 123 | 1 | 1.864 | 0.659 | -1.205 | 0.519 | 0.422 | 0.737 |
| 124 | 1 | 1.034 | 0.671 | -0.363 | 0.576 | 0.354 | 0.554 |
| 125 | 1 | 1.315 | 0.673 | -0.642 | 0.603 | 0.33 | 0.497 |
| 126 | 1 | 0.448 | 0.733 | 0.285 | 0.274 | 0.669 | 2.04 |
| 127 | 1 | 0.511 | 0.906 | 0.395 | 0.222 | 0.723 | 2.641 |
| 128 | 1 | 0.695 | 0.436 | -0.259 | 0.557 | 0.377 | 0.612 |
| 129 | 1 | 2.043 | 1.03 | -1.014 | 0.488 | 0.453 | 0.838 |
| 130 | 1 | 1.236 | 1.637 | 0.401 | 0.46 | 0.458 | 0.853 |
| 131 | 1 | 1.042 | 0.363 | -0.68 | 0.788 | 0.168 | 0.204 |
| 132 | 1 | 0.717 | 0.332 | -0.385 | 0.624 | 0.308 | 0.45 |
| 133 | 1 | 0.466 | 0.327 | -0.139 | 0.568 | 0.355 | 0.556 |
| 134 | 1 | 0.733 | 0.788 | 0.054 | 0.343 | 0.597 | 1.496 |
| 135 | 1 | 0.6 | 2.975 | 2.375 | 0.015 | 0.955 | 21.584 |
| 136 | 1 | 3.268 | 0.369 | -2.899 | 0.655 | 0.287 | 0.407 |
| 137 | 1 | 0.497 | 1.509 | 1.012 | 0.092 | 0.868 | 6.667 |
| 138 | 1 | 0.506 | 0.656 | 0.15 | 0.333 | 0.606 | 1.551 |
| 139 | 1 | 0.577 | 0.789 | 0.212 | 0.315 | 0.625 | 1.682 |
| 140 | 1 | 0.689 | 0.313 | -0.376 | 0.772 | 0.179 | 0.221 |
| 141 | 1 | 0.53 | 1.904 | 1.374 | 0.068 | 0.896 | 8.72 |
| 142 | 1 | 0.539 | 0.704 | 0.164 | 0.328 | 0.611 | 1.589 |
| 143 | 1 | 0.544 | 5.533 | 4.989 | 0.001 | 0.988 | 84.624 |
| 144 | 1 | 0.486 | 0.606 | 0.119 | 0.347 | 0.591 | 1.459 |
| 145 | 1 | 0.496 | 0.337 | -0.16 | 0.575 | 0.351 | 0.546 |
| 146 | 1 | 0.597 | 6.008 | 5.411 | 0.002 | 0.985 | 66.961 |
| 147 | 1 | 0.392 | 0.321 | -0.072 | 0.522 | 0.393 | 0.655 |
| 148 | 1 | 0.53 | 0.383 | -0.147 | 0.552 | 0.377 | 0.612 |
| 149 | 1 | 0.721 | 0.423 | -0.298 | 0.561 | 0.372 | 0.6 |
| 150 | 1 | 0.466 | 0.299 | -0.166 | 0.592 | 0.33 | 0.498 |
| 151 | 1 | 1.036 | 0.338 | -0.698 | 0.805 | 0.153 | 0.183 |
| 152 | 1 | 0.731 | 3.274 | 2.543 | 0.006 | 0.962 | 25.893 |
| 153 | 1 | 0.721 | 0.423 | -0.298 | 0.561 | 0.372 | 0.6 |
| 154 | 1 | 4.11 | 0.43 | -3.68 | 0.834 | 0.132 | 0.154 |
| 155 | 1 | 0.537 | 2.698 | 2.16 | 0.023 | 0.952 | 19.993 |
| 156 | 1 | 0.563 | 0.298 | -0.265 | 0.63 | 0.298 | 0.429 |
| 157 | 1 | 2.15 | 4.575 | 2.425 | 0.091 | 0.719 | 2.588 |
| 158 | 1 | 1.114 | 0.608 | -0.507 | 0.433 | 0.506 | 1.035 |
| 159 | 1 | 0.793 | 8.803 | 8.011 | 0.009 | 0.975 | 38.923 |
| 160 | 1 | 0.647 | 9.254 | 8.606 | 0.002 | 0.988 | 85.594 |
| 161 | 1 | 2.85 | 0.797 | -2.052 | 0.613 | 0.323 | 0.483 |
| 162 | 1 | 0.532 | 0.696 | 0.165 | 0.328 | 0.611 | 1.588 |
| 163 | 1 | 0.556 | 7.686 | 7.13 | 0 | 0.995 | 210.153 |
| 164 | 1 | 0.718 | 0.335 | -0.383 | 0.622 | 0.31 | 0.455 |
| 165 | 1 | 0.515 | 6.453 | 5.938 | 0.001 | 0.994 | 164.329 |
| 166 | 1 | 0.496 | 0.322 | -0.174 | 0.588 | 0.338 | 0.515 |
| 167 | 1 | 1.133 | 2.987 | 1.853 | 0.065 | 0.838 | 5.234 |
| 168 | 1 | 0.548 | 0.701 | 0.153 | 0.331 | 0.608 | 1.565 |
| 169 | 1 | 0.531 | 1.384 | 0.854 | 0.13 | 0.823 | 4.715 |
| 170 | 1 | 0.945 | 0.369 | -0.576 | 0.774 | 0.18 | 0.221 |
| 171 | 1 | 0.506 | 0.607 | 0.101 | 0.355 | 0.582 | 1.407 |
| 172 | 1 | 0.91 | 2.001 | 1.091 | 0.104 | 0.844 | 5.455 |
| 173 | 1 | 2.67 | 0.508 | -2.162 | 0.587 | 0.355 | 0.556 |
| 174 | 1 | 0.47 | 0.489 | 0.019 | 0.464 | 0.466 | 0.883 |
| 175 | 1 | 0.563 | 0.29 | -0.273 | 0.637 | 0.291 | 0.414 |
| 176 | 1 | 0.717 | 0.332 | -0.385 | 0.624 | 0.308 | 0.45 |
| 177 | 1 | 0.764 | 0.316 | -0.447 | 0.788 | 0.166 | 0.202 |
| 178 | 1 | 0.504 | 0.629 | 0.125 | 0.344 | 0.594 | 1.476 |
| 179 | 1 | 0.544 | 0.944 | 0.4 | 0.227 | 0.718 | 2.573 |
| 180 | 1 | 0.718 | 0.689 | -0.029 | 0.372 | 0.566 | 1.321 |
| 181 | 1 | 0.497 | 0.338 | -0.159 | 0.574 | 0.352 | 0.549 |
| 182 | 1 | 0.393 | 0.363 | -0.03 | 0.488 | 0.431 | 0.765 |
| 183 | 1 | 0.919 | 0.633 | -0.286 | 0.575 | 0.355 | 0.556 |
| 184 | 1 | 0.567 | 0.422 | -0.145 | 0.538 | 0.393 | 0.654 |
| 185 | 1 | 0.554 | 7.72 | 7.165 | 0 | 0.995 | 221.3 |
| 186 | 1 | 0.911 | 0.845 | -0.066 | 0.438 | 0.482 | 0.94 |
| 187 | 1 | 0.882 | 2.725 | 1.844 | 0.045 | 0.885 | 7.8 |
| 188 | 1 | 0.716 | 0.309 | -0.408 | 0.643 | 0.289 | 0.411 |
| 189 | 1 | 0.467 | 0.343 | -0.124 | 0.555 | 0.369 | 0.591 |
| 190 | 1 | 0.563 | 0.298 | -0.265 | 0.63 | 0.298 | 0.429 |

Table S24 Primers used for vectors construction and transgenetic materials identification

| **Gene** | **Sequences (5’→3’)** |
| --- | --- |
| *RolB* | F: GCTCTTGCAGTGCTAGATTT  R: GAAGGTGCAAGCTACCTCTC |
| *35S* | F: GAGGACCTAACAGAACTCGCC |
| *SmWRKY61* | F: GGGGACAAGTTTGTACAAAAAAGCAGGCTTAATGGAAAGCGAGTATCTTGAGCTGT  R: GGGGACCACTTTGTACAAGAAAGCTGGGTACTAAAGGCCTGGGCATGTATGCTGT |

Table S25 Reaction system for BP clone

| **Item** | **Amount** |
| --- | --- |
| attB-PCR product | 150 ng |
| p DONR207 vector | 150 ng |
| TE buffer(pH=8.0) | up to 9 μl |
| Gateway®BP Clonase® II Enzyme mix | 1 μl |

Table S26 Reaction system for LR clone

| **Item** | **Amount** |
| --- | --- |
| pDONR207-target gene | 150 ng |
| pK7WG2R vector | 150 ng |
| TE buffer(pH=8.0) | up to 9 μl |
| Gateway®LR Clonase® II Enzyme mix | 1 μl |

Table S27 List of RT-qPCR primers

| **Gene** | **Genbank ID** | **Sense Primer (5’ to 3’)** | **Reverse Prime (5’ to 3’)** |
| --- | --- | --- | --- |
| *4CL1* | AY237163.1 | CGCCCATGGATAGAGAACTT | ACCATAACCCTGGCCAATAA |
| *4CL10* | KF220563.1 | ACAGGGCAGCACTATCACTG | GGAGATTTCGGAATGGAGTC |
| *4CL2* | AY237164.1 | CCCTCCTCCTCAATAATCCA | AGCTTGCTCATCTTGCATTG |
| *4CL3* | KF220556.1 | CGGCGACAACCCTAATTTAT | CAGAGAAGCACCGAATTCAA |
| *4CL5* | KF220558.1 | GCATACGGGAGATCTTGGAT | TGATCAGCTCTTTGATTCGG |
| *4CL6* | KF220559.1 | CACATGATGACTCGGAGGAC | TCCCGGACGAGAAGAGTATC |
| *4CL7* | KF220560.1 | GGCGAAGCTCAATATTCCTC | GGAGATAGGACGATTTCCGA |
| *4CL9* | KF220562.1 | TTTCATTCAGGGTTATGGCA | TGACCCAGTCCACCACTTTA |
| *AACT1* | EF635969.1 | GTCGTGGCTCTTGCTAATCA | CGAGAGGATGCCCTAGAGAC |
| *AACT2* | JN831101.1 | AGCCCTTTGAAACAATTTGG | AAACTTCCCTTTGTGTTGCC |
| *AACT3* | SMil_00022001 | TTGGTGCTGGGATAGAATCA | TGGAAGAAGACAATCTTGCG |
| *AACT4* | SMil_00010450 | AGAGCTATTGAGTGCCGGAT | TTTATGAAGGCAGCGACATC |
| *AACT5* | SMil_00009587 | TCAATGTGAATGGAGGTGCT | AGCAAGGTAGCAACACAACG |
| *AACT6* | SMil_00030406 | TCAAAGAGAGGTGGCTTCAA | GGCATAATCTCACGAACCCT |
| *C4H* | DQ355979.1 | AGGTCAGGACATGGTGTTCA | CACCTTGTTGGTGAAGAACG |
| *CMK* | EF534309.1 | TGGGCAGCAAATCAGTTTAG | CGACGACTTCTCCTCTACCC |
| *CPR1* | FR693803.1 | GAGTGTTCGGTCTTGGGAAT | GCTTCTTTCCTCCTTGTTCG |
| *CPR2* | JX848592.1 | CAACATGGATGAAGAATGCC | AAATGGAGCCAAACCAGTTC |
| *CPS1* | EU003997.1 | GCGACATTGACGATACATCC | GCTTGAAATTCCTCAGCACA |
| *CPS3* | JN831115.1 | TGGAAGACTTGGATTGGTCA | GGCAGTTGATGATGGAGATG |
| *CPS4* | JN831120.1 | GAGGGTTCGTTTCTTTCGTC | CCGTTGAATTTGTTGACAGG |
| *CPS5* | JN831121.1 | CCAAATGTGTACCCTGTGGA | TATGCGACACATTCCTCGAT |
| *CPS8* | SMil_00017335 | TGGAAGACTTGGATTGGTCA | GGCAGTTGATGATGGAGATG |
| *CPS9* | SMil_00001997 | AATCCGATTTGCAGGAGAAG | GGCATCTCCAATCCAACTTT |
| *CYP76AH1* | JX422213.1 | CTCATGCTGGACTTGTTCGT | TTCAGTCGAGCCATCTTGTC |
| *CYP76AH3* | KR140168.1 | GAGAGCGATCAAGTCGTCAA | CTGGATTGTTCCAGATGGTG |
| *CYP76AK1* | KR140169.1 | GCTAATCGGAAATTCAGGGA | GGATCCAAAGGTGCAAAGAT |
| *CYP98A14* | HQ316179.1 | CTGCCCTACTTGCAGTGTGT | GATCTCGACATTGGTGTTGG |
| *DXS1* | EU670744.1 | AACCAATCCCACCAACATTT | GCCACTGTGTGTGTGTGTGT |
| *DXS2* | FJ643618.1 | ACAAGCTCGATCTCATGCAC | AATCCTTCCCTTCCCAATCT |
| *DXS3* | JN831116.1 | TGGGCTGAATTTGGTGTTTA | CTGCAGTCATTGCTCCATCT |
| *DXS4* | JN831117.1 | CGACGTAGACAGGCAGAAGA | CCATCACGATCATGTTAGGC |
| *FPPS* | EF635968.1 | GAATGGCTCCAGGCATATTT | GGATATGGTTCCGGAGAATG |
| *GGPPS1* | FJ178784.1 | CCATGTCTCTCATCCACGAC | CGAAGACCTTGTGATTGGTG |
| *GGPPS2* | JN831112.1 | TGTTCACCTTCTTTGCCTTG | AAACCAGGATTTCCTGCAAC |
| *GGPPS4* | SMil_00021009 | GCAGATGGTGGATATCTGCTC | GTGGACGAACTCCAGGTGAT |
| *GPPS* | JN831107.1 | AGATGCGCTCTATTGTGTGG | TCGACAGTTCATCAGCAACA |
| *GPPS.LSU* | JN831111.1 | AAGAGCGTGGAAGCAGATTT | ACACAGGTTGTAGCGAGTGG |
| *GPPS.LSU2* | SMil_00010060 | CAAGGACAACGACGACCTC | CCAGGACTGCAACGTCTTC |
| *GPPS.SSUI* | JN831108.1 | CGGAGCCGAAGAAGAAATAC | TCCTTGAGAGCCAAATCCTT |
| *GPPS.SSUI2* | SMil_00026169 | CGGAGCCGAAGAAGAAATAC | TCCTTGAGAGCCAAATCCTT |
| *GPPS.SSUII.1* | JN831109.1 | GAGGAGATTGAGCGGTTAGG | TCTTCCACCACTCGGTACAA |
| *GPPS.SSUII.2* | JN831110.1 | TTCCGTCATATTGTTTCCCA | GAAACTGACCAGCAGCCATA |
| *GPPS.SSUII.3* | SMil_00006505 | TTCCGTCATATTGTTTCCCA | GAAACTGACCAGCAGCCATA |
| *HCT1* | GU647199.1 | ATTGAGATCAACTGCAACGC | GGCGAAGTCTCCGTAATCAT |
| *HDR* | JX516088.1 | CCTGTCACAATTGGCGTTAC | CTTCTGCTTGCTTCATCTCG |
| *HDR1* | JN831099.1 | CCTGTCACAATTGGCGTTAC | ATGGGAAACAAGAACCAAGC |
| *HDR2* | JN831100.1 | GACTTGCATTGCTGATGCTT | TCGCAAATGGTGTTGAAACT |
| *HDR3* | SMil_00028353 | GTGATGTGGTGGTATTGCCT | ACCCATGGGCAAGTTGTATC |
| *HDR4* | SMil_00016128 | CTTACGAAGCTCGACACCAA | ATCTCTCCCAGCGTCTGATT |
| *HDS2* | KJ746807.1 | AAGCGAGCAACCCAGTTATT | CCTATTGCCGATTTCATCCT |
| *HMGR1* | GU367911.1 | TTCACCCTCTTCTTCTCCGT | GGAATTGCGGATCTTCTCAC |
| *HMGR2* | FJ747636.1 | CGCCTCCTTCATCTATCTCC | TCCTTGAGCATCAAGTCGTC |
| *HMGR3* | JN831102.1 | ATGCTAGCAACATCGTCTCG | GCAAGGCATCGTTACTGAGA |
| *HMGR4* | JN831103.1 | ACATTGTGTCTGCCGTGTTT | CATCATTGACAGCCTCCATC |
| *HMGS* | FJ785326.1 | CGGTTACTTCGCTTCTAGGG | TTTGCTCTTGTCGAGTACGG |
| *HMGS2* | SMil_00020797 | AACATCCTTTCAGCCTGTCC | ATGAGCTGCATCAGTTCGAC |
| *HPPD* | EF157837.1 | GATAGGCCGACCATCTTCAT | CCTGAAGAGCTCCGAGAAGT |
| *HPPR* | DQ099741.1 | GGTTCAAGCTCTTCCGCTAC | GAGTTGGAGTTCCCGACAAT |
| *HPPR2* | KF220565.1 | CTCTGACCACGTTGAGGAGA | ATTTCTCCACGATCCATTCC |
| *HPPR3* | KF220566.1 | CATTGTTGTGTCGTGTGCAT | AGCAAGGGCAGAGATCAACT |
| *IDI2* | JN831106.1 | AGCCTTCAGCGTGTTTCTCT | GTCCACACAAGAGGGAAGGT |
| *IDI3* | SMil_00003370 | CCAAATACAACTGCCACCTG | GTCCACACAAGAGGGAAGGT |
| *IDI4* | SMil_00003369 | CTCGATCACTTCCTTCCGTT | ACTTCATCAGGGTTCGGTTC |
| *IDI5* | SMil_00029094 | AGCCTTCAGCGTGTTTCTCT | GTCCACACAAGAGGGAAGGT |
| *KSL1* | EF635966.1 | AATTGTCCGATGTTCGCATA | GCTGGCTTCTCTTTCCATTC |
| *KSL2* | JN831119.1 | TGCTATTCCCATAACGTCCA | GCTTGAATGCTTTGTCTCCA |
| *KSL3* | SMil_00014024 | CTGTAACTGCTGTCGGAGGA | GGCTTCGTCTCGTAGGTAGC |
| *MCS* | JX233816.1 | ATGCCACATTGATTCTCCAA | TAGCCTTGAGGTTCACAACG |
| *MDC* | JN831105.1 | TTGTGTGCGCTGTAACTTCA | AAGCAAACTGCATGGAACTG |
| *MK* | JN831104.1 | TCAAGGATGTGGACTTGGAA | TGATGAAGGTGAGGAAGCAG |
| *PAL1* | EF462460.1 | GGGAGACGCTCACCATATCT | CCATAACCCAATCACTGCTG |
| *PAL2* | GQ249111.1 | TGGAATATTCGGCAACGATA | TTACTAGCATCGAGGCCCTT |
| *PAL3* | KF220569.1 | CACGGAATCAAACCACACTC | TGACGTTGTGGTTGAGGAAT |
| *PMK* | JN831095.1 | AGCACCTTGGAGAGCTCATT | AAGGAGGAAGTGAAGCCAAA |
| *RAS* | FJ906696.1 | ACTTCGGTAACGCCATCTTC | TCTAAGAAATCGAGCGACGA |
| *RAS2* | KF220570.1 | TGCTTCAGTTGACTCGGTTC | ACCACGTGTTGATGAAATGG |
| *RAS3* | KF220571.1 | TCAACGTTCAAGCTCACTCC | GTAGGTGGTGTAGCCGGATT |
| *RAS4* | KF220572.1 | TCGGCAACGTGATCTTCTAC | AGCCGACCTCAAATACTCGT |
| *RAS5* | KF220573.1 | CCGACACATCACACTCAACA | GTAGCCGGATTTGCATCTCT |
| *RAS6* | KF220574.1 | AACTTCTTCGACGTTGCCTT | GTTGCAGTTGATCTGGATGC |
| *TAT1* | DQ334606.1 | CATTTGCAGTAGACGTGCCT | GAGTGCCGTTCACAGAAAGA |
| *TAT2* | KF220575.1 | CGTGATACTCCTCCCAGGTT | GTTCGATGGCAAATGTAACG |
| *actin* | HM231319.1 | GGTGCCCTGAGGTCCTGTT | AGGAACCACCGATCCAGACA |

Table S28 List of CAS of standards for quantification by HPLC

| Chemicals | Chemical formula | CAS |
| --- | --- | --- |
| Caffeic acid | C₉H₈O₄ | 331-39-5 |
| Rosmarinic acid | C_18_H_16_O_8_ | 20283-92-5 |
| Salvianolic acid B | C_36_H_30_O_16_ | 115939-25-8 |
| Cryptotanshinone | C_19_H_20_O_3_ | 35825-57-1 |
| Tanshinone IIA | C₁₉H₁₈O₃ | 568-72-9 |
| Tanshinone I | C₁₈H₁₂O₃ | 568-73-0 |
| Dihydrotanshinone | C_18_H_14_O_3_ | 20958-18-3 |
